# Unravelling the nexus of non-coding RNAs in cancer stemness and therapeutic drug resistance

**DOI:** 10.3389/fcell.2026.1870002

**Published:** 2026-08-06

**Authors:** Song Chen, Tikam Chand Dakal, Ravi Bhushan, Dilip Kumar Arya, Bhaskar Gogoi, Abhishek Kumar, Ingo G. H. Schmidt-Wolf, Amit Sharma, Meiling Chen

**Affiliations:** 1 Department of Orthopedics, The First College of Clinical Medical Sciences, China Three Gorges University, Yichang, China; 2 Yichang Central People’s Hospital, Yichang, China; 3 Genome and Computational Biology Lab, Department of Biotechnology, Mohanlal Sukhadia University, Udaipur, India; 4 Centre for Genetic Disorders, Institute of Science, Banaras Hindu University, Varanasi, India; 5 University School of Pharmaceutical Sciences, Rayat Bahra Professional University, Punjab, India; 6 Mizoram University, Mizoram, India; 7 Institute of Bioinformatics, Bangalore, India; 8 Manipal Academy of Higher Education (MAHE), Manipal, India; 9 Department of Integrated Oncology, Center for Integrated Oncology (CIO), University Hospital of Bonn, Bonn, Germany; 10 Department of Hematology, Fujian Institute of Hematology, Fujian Provincial Key Laboratory on Hematology, Fujian Medical University Union Hospital, Fuzhou, China

**Keywords:** cancer stem cells, drug resistance, long non-coding RNAs, MicroRNAs, non-coding RNAs, signalling pathways

## Abstract

Cancer stem cells (CSCs) play a pivotal role in tumor initiation, progression, and therapy resistance. Emerging evidence suggests that non-coding RNAs (ncRNAs), including microRNAs (miRNAs) and long non-coding RNAs (lncRNAs), intricately regulate CSC properties. This article reviews current knowledge on the intricate interplay between ncRNAs, genetic and epigenetic factors having role in cancer stemness and associated phenotypes. We have also identified key stemness- and EMT-associated genes, including *OCT4*, *SOX2*, *NANOG*, *KLF4*, *CD44*, *ALDH1A1*, *BMI1*, *ZEB1*, *ZEB2*, *SNAIL*, *SLUG*, and *TWIST1* that are regulated by ncRNAs in different cancer types. Additionally, we have described how ncRNA-mediated regulation of these genes influences major signaling pathways, including Wnt/β-catenin, Notch, Hedgehog, PI3K/AKT/mTOR, JAK/STAT, NF-κB, Hippo/YAP, and TGF-β signaling. The mir-21, miR-34, miR-200, and let-7 families target self-renewal and epithelial-to-mesenchymal transition while lncRNAs like H19, HOTAIR, and MALAT1 remodel the regulatory and epigenetic landscape of cancer stem cells. Understanding the interplay between ncRNAs and CSCs offers new insights into potential targeted therapies for combating aggressive and therapy-resistant cancers. Additionally, combining ncRNA interventions with conventional modalities such as chemotherapy, epigenetic drugs along with the advance nanotechnology-based drug delivery systems could result into synergistic outcomes. However, delivery, efficacy, and safety challenges remain. Overall, the ncRNA-cancer stemness interplay warrants therapeutic advancements and clinical translation towards a promising treatment personalization.

## Introduction

Cancer stemness and epithelial–mesenchymal transition (EMT) have been identified as key drivers of tumor initiation, metastatic dissemination, disease recurrence and therapy resistance within the mechanisms that underlie these processes. It is well acknowledged that cancer stem cells (CSCs), a subpopulation of tumor cells with the potential for self-renewal, differentiation, and tumor initiation, drive continued tumor growth and play a major role in treatment failure (Allgayer et al., 2025). Simultaneously, EMT causes epithelial cancer cells to develop mesenchymal traits, which confer migratory, invasive, and survival properties. There is increasing evidence that cancer stemness and EMT are not separate phenomena but rather interconnected and highly plastic biological programs that together contribute to tumor progression and therapeutic resistance (Jimenez-Castaño and Nieto, 2026).

Stem-like and mesenchymal phenotypes are acquired and maintained through complex molecular networks including stemness-associated transcription factors (e.g., OCT4, SOX2, NANOG, KLF4, and BMI1) and EMT regulators (e.g., SNAIL, SLUG, TWIST, ZEB1, and ZEB2) (Nwokolo et al., 2025). These factors are modulated by multiple signaling pathways, including Wnt/β-catenin, Notch, Hedgehog, TGF-β, PI3K/AKT/mTOR, JAK/STAT, NF-κB and Hippo/YAP signaling. Aberrant activation of these pathways contributes to cellular plasticity, promotes adaptation to therapeutic stress and allows the emergence of drug-tolerant persister populations that ultimately drive tumor relapse and metastasis (Xue et al., 2025). Thus, understanding the regulatory mechanisms that control CSC maintenance and EMT has gained great interest in cancer research and is an important direction for the development of more effective therapeutic strategies (Liu T. M. et al., 2025).

Non-coding RNAs (ncRNAs) have emerged as critical regulators of gene expression and determination of cellular fate in the last decade. ncRNAs, once thought of as by-products of transcription, are now known to be integral components of gene regulatory networks that impact nearly all aspects of cancer biology (Chen and Kim, 2024). MicroRNAs (miRNAs), long non-coding RNAs (lncRNAs) and circular RNAs (circRNAs) are major classes of ncRNAs that regulate gene expression through distinct, but often interconnected, mechanisms (Chodurska and Kunej, 2025). miRNAs mainly act through post-transcriptional repression of target messenger RNAs (mRNAs). lncRNAs and circRNAs regulate transcriptional programs, chromatin organization, epigenetic modifications, RNA stability, and protein activity. ncRNAs, via these different mechanisms, modulate important signaling pathways and transcriptional networks that regulate stemness, EMT, metastasis, immune escape and therapy response (Hossam Abdelmonem et al., 2025).

There is accumulating evidence that ncRNAs are master regulators of cancer stemness and EMT. Many oncogenic and tumor suppressive ncRNAs regulate the expression of stemness-associated genes and EMT transcription factors, thereby affecting CSC self-renewal, differentiation, cellular plasticity and metastatic potential (Peng et al., 2025). Furthermore, ncRNAs function in complex regulatory circuits with chromatin remodelers, epigenetic modifiers, transcription factors and signaling intermediates, thus allowing their integration of extracellular cues from the TME with intracellular signaling programs (Yin and Shen, 2023). Redox homeostasis is a critical determinant of CSC maintenance, enabling CSCs to balance reactive oxygen species (ROS) production with antioxidant defenses to preserve self-renewal, metabolic flexibility, and long-term survival (Du et al., 2024). Importantly, dysregulated ncRNA expression has been implicated in resistance to chemotherapy, radiotherapy, targeted therapies and immunotherapies in a wide variety of malignancies. These findings position ncRNAs at the intersection of cancer stemness, EMT and therapy resistance underscoring their role as mechanistic drivers and potential therapeutic targets (Dakal et al., 2024).

Besides their biological importance, ncRNAs have a great translational potential. Their tissue specific expression patterns, remarkable stability in biological fluids and capability to regulate multiple oncogenic pathways simultaneously make them attractive candidates for diagnostic, prognostic and predictive biomarkers (Eldakhakhny et al., 2024). Among ncRNAs, microRNAs (miRNAs) are key post-transcriptional regulators that orchestrate cancer stemness, epithelial–mesenchymal transition (EMT), tumor progression, and therapeutic resistance by fine-tuning the expression of multiple oncogenes, tumor suppressors, and signaling pathways (Wang et al., 2024). Recent advances in RNA therapeutics, such as antisense oligonucleotides, small interfering RNAs (siRNAs), miRNA mimics, CRISPR-based genome editing, and nanoparticle-mediated delivery systems, have provided new opportunities to target ncRNA-regulated stemness networks (Singh et al., 2026). Thus, ncRNA-based therapeutic strategies are currently being explored as new approaches to deplete CSC populations, inhibit EMT-driven metastasis, and overcome treatment resistance (John et al., 2025).

Here, we discuss the nexus and emerging roles of ncRNAs in regulation of cancer stemness, EMT and therapeutic resistance (Table 1). By shedding light on this nexus, we aspire to contribute to the growing body of knowledge that may hold the key to novel and targeted therapeutic interventions, ultimately advancing the prospects of combating aggressive and therapy-resistant cancers. We also discuss the molecular mechanisms by which miRNAs, lncRNAs and circRNAs regulate transcriptional programs associated with stemness, regulators of EMT, epigenetic modifications and oncogenic signaling pathways. We also review the regulatory networks linking ncRNAs to CSC maintenance and drug resistance, summarize recent advances in ncRNA-based therapeutics and clinical translation, and highlight future opportunities to exploit ncRNA-mediated pathways to improve cancer treatment outcomes.

**TABLE 1:** List of miRNAs and lncRNAs related to different cancers.

Cancer types	miRNA	lncRNA	References
Breast cancer	miR-21, miR-125b, miR-145	DANCR, NR2F1-AS1 (NAS1), NEAT1, NEAT1, NRAD1, LINC-ROR, LINC01133, linc00617, CCAT1, RP1-5O6.5 (RP1), lncRNA-Hh	da Silva et al. (2026), Xiao et al. (2025)
Prostate cancer	miR-21, miR-221, miR-222, miR-181a, miR-181b, miR-181d, miR-155, miR-196a, miR-217, let-7i, miR-100, miR-155, miR-221, miR-301, miR-21, miR-181a, miR-125b, miR-212, miR-376a, miR-375, miR-103, miR-107, miR-23a, miR-26b, miR-342, miR-192, miR-204, miR-211, miR-21, miR-155	PCA3, LincRNA-p21, PCAT-18, MALAT1, PVT1, TRPM2	Xiao et al. (2025), Singh et al. (2024)
Pancreatic cancer	miR-21, miR-221, miR-222, miR-181a, miR-181b, miR-181d, miR-155, miR-196a, miR-217, let-7i, miR-100, miR-155, miR-221, miR-301, miR-21, miR-181a, miR-125b, miR-212, miR-376a, miR-375, miR-103, miR-107, miR-23a, miR-26b, miR-342, miR-192, miR-204, miR-211, miR-21, miR-155	HOTTIP, HOTAIR, H19, PVT1, MALAT1, DAPK1, MIR31HG, HULC, LINCRNA-ROR	Mir et al. (2025), Hasani et al. (2025)
Lung cancer	miR-21, miR-205, miR-126	MALAT1, SPRY4-IT1, ANRIL, NEAT1, UCA1	Elimam et al. (2025), Bitaraf et al. (2025)
Ovarian cancer	miR-199a, miR-140, miR-145, miR-125b1	NEAT1	Alshamrani (2020), Ravindran et al. (2021)
Uterine leiomyoma	let-7, miR-21, -23b	​	Watrowski et al. (2025)
Hepatocellular cancer	miR-21, miR-221, let-7a, miR-122a, miR-224, miR-18, miR-199a, miR-199a, miR-200a	HOTAIR, uc001ncr, AX800134, HULC, Linc00152, RP11-160H22.5, XLOC014172, LOC149086, HEIH, XLOC014172, LOC149086	Zarlashat et al. (2025), Zhang B. et al. (2026)
Thyroid cancer	miR-30d, miR-125b, miR-26a, and miR-30a-5p, miR-197, miR-346, miR-221, miR-222, miR-181b, miR-221, miR-222, miR-146b	NEAT1, HOTAIR, PTCSC2, lncRNA TNRC6C-AS1, GAS8-AS1, PTCSC3, MEG3, BANCR, PVT1, SPRY4-IT1, GAS5, H19, CASC2, and MALAT1	Saiselet et al. (2016), Li et al. (2023)
Colorectal cancer	miR-25, miR-92, miR-31, miR-96, miR-135b, miR-183, miR-133b, miR-145	HOTAIR, CCAT1, CCAT2, FER1L4, XLOC_006844, LOC152578, XLOC_000303	Włodarczyk et al. (2025), Pająk et al. (2025)
Leukaemia	miR-128a, miR-128b, let-7b, miR-223, miR-21, miR-150, miR-155, miR-92, miR-222, miR-29b, miR-181b, miR-16-1, miR-15a	MEG3, H19, UCA1, HOTAIR, CRNDE, PANDAR, PVT1, CASC15, IRAIN, RUNXOR, CCAT1, CCDC26, TUG1, MALAT, HOXA-AS2, MONC, NEAT1, ALL, BALR-6, CASC15, GAS5, HOXA-AS2, ZEB1-AS1, NEAT1, PVT1, SNHG16, NALT, LncR1, LUNAR1, XLOC_001561, ANRIL, Linc-PINT, Lnc-INSR, ARIEL, RP11-137H2.4, HOTAIR, MEG3, H19, HAND2-AS1, HULC, MALAT1, NEAT1, SNHG5, UCA1, PLIN2, FENDRR, BGL3, CLL, MALAT1, MIAT, GATA6-AS1, TRERNA1, lncRNA-p21	Rashed et al. (2019)
Renal cancer	miR-124, miR-381, miR-451, miR-375, miR-497, miR-451, miR-137, hsa-miR-101, miR-203, miR-144, miR-137	LET; PVT1; PANDAR; PTENP1; LINC00963	Kathuria-Prakash et al. (2024), Ghafouri-Fard et al. (2020)
Cervical cancer	miR-1284, miR-573, miR-433, miR-424-5p, miR-361-5p, miR-383-5p, miR-335-5p, miR-874, miR-132, miR-411, miR-96-5p, miR-337-3p, miR-199b-5p, miR-3941, miR-93, miR-545, miR-200a, miR-143, miR-107, miR-1, miR-139-3p, miR-224, miR-92a, miR-195, miR-31, miR-2861	MEG3, PVT1, H19, FAM83H-AS1, MALAT1, PAX8 AS1, CCAT2, C5orf66-AS1, SPRY4-IT1, CCAT1, GAS5, NOC2L-4.1, CCHE1, HOTAIR, EBIC, RSU1P2, LINC00675	Parvizi et al. (2025), Endale et al. (2024)

## Cancer stemness, EMT and drug resistance

Cancer progression is not solely driven by genetic mutations but also by dynamic cellular states that enable tumors to adapt, survive, and recur under therapeutic pressure. Among these, CSCs represent a highly plastic subpopulation capable of self-renewal, multilineage differentiation, and long-term tumor maintenance. EMT, cancer stemness, and drug resistance are closely interconnected processes that collectively contribute to tumor progression, metastasis, and therapeutic failure (Shang et al., 2025). During EMT, epithelial cancer cells lose cell–cell adhesion and acquire mesenchymal characteristics, resulting in enhanced migratory, invasive, and survival capabilities (Bhushan et al., 2026). A growing body of research suggests that CSCs and tumor precursor-like cells constitute a unique cell subpopulation seen across malignancies (Liang et al., 2025). In addition to causing intratumoral heterogeneity, their abilities for self-sustaining, proliferation, and biochemical modification allow for treatment evasion, which promotes cancer recurrence and dissemination (Zhang Z. et al., 2025). Through epigenetic, developmental, and microscopic reconfiguration, these stem subpopulations demonstrate intrinsic flexibility, promoting clonal diversity and allowing survival in androgen-deprived environments (Zhang X. et al., 2025). Chemoresistance is still a serious problem in cancer therapy, which accounts for most patient relapses and low survival rates despite advances in cancer treatment (Wahab and Siddique, 2025).

Recent developments have shed new light on the mechanisms behind cancer cell dormancy, particularly with regard to how dormant cells change over time and become resistant to therapies (Khan et al., 2025). Tumor start, development, metastasis, and recurrence are all driven by CSCs, a highly malleable and treatment-resistant cell subset (Lee et al., 2025). Drug resistance has been better understood since the idea of CSCs, an uncommon and naive subset with cancer-causing ability that helps generate intramuscular variability, was introduced (Tan et al., 2025). Over the past 3 decades, research on TME dynamics has influenced global perspectives on medication resistance in cancer (Duan et al., 2025). Increasing evidence suggests that CSCs lie at the core of therapeutic failure, disease relapse, and metastatic spread across diverse cancer types. Their remarkable adaptability is closely intertwined with molecular mechanisms governing drug resistance, cellular plasticity, and microenvironmental interactions.

### Cancer stem cells (CSCs): functional plasticity and implications

A defining hallmark of CSCs is their remarkable functional plasticity, which enables them to adapt to diverse microenvironmental conditions and therapeutic pressures. Rather than representing a static cell population, CSCs exist in dynamic and reversible states that facilitate tumor initiation, progression, metastasis, and treatment resistance. This adaptive behavior is driven by complex interactions among genetic, epigenetic, transcriptional, and metabolic regulatory networks that allow CSCs to modify their cellular identity, phenotype, and metabolic programs in response to environmental cues (Loh and Ma, 2024). Consequently, the plastic nature of CSCs contributes significantly to intratumoral heterogeneity, tumor recurrence, and the emergence of therapy-resistant cell populations. Among the various dimensions of CSC adaptability, cellular, phenotypic, and metabolic plasticity have emerged as key mechanisms underlying cancer progression and therapeutic failure (Lee et al., 2025).

Cancer stem cells were first identified based on their ability to initiate tumors in immunocompromised hosts and to recapitulate tumor heterogeneity. Unlike bulk tumor cells, CSCs exhibit long-term self-renewal capacity and differentiation potential reminiscent of normal stem cells, albeit in a dysregulated manner. These cells are often characterized by the expression of specific surface markers such as CD44, CD133, ALDH1, and EpCAM, although marker expression varies across cancer types and microenvironmental contexts (Haddadin and Sun, 2025). Cancer stem cells are responsible for the start, dissemination, and resurgence of cancer cells, a highly aggressive behaviour responsible for higher-grade malignancies. A complicated molecular interactions and physiological processes are involved in the formation of cancer stemness, which ultimately lead to a malignant tumor with a high morbidity and fatality rate (Lou et al., 2025).

#### CSCs and phenotypic plasticity

A defining feature of CSCs is their remarkable phenotypic plasticity, which enables dynamic transitions between stem-like and differentiated cellular states in response to microenvironmental signals, therapeutic pressure, and metabolic stress (Keoh et al., 2025). Phenotypic plasticity describes the capacity of CSCs to reversibly alter their functional and molecular characteristics without permanent genetic changes. This adaptive capability allows tumors to maintain cellular heterogeneity and regenerate CSC populations even after apparent therapeutic elimination. EMT represents a key manifestation of phenotypic plasticity, enabling tumor cells to acquire stem-like properties, enhanced motility, and increased resistance to apoptosis. This adaptability allows CSCs to survive fluctuating environmental conditions and therapeutic challenges (Bhat et al., 2024). One major obstacle to successful treatment results is susceptibility to specific medications for cancer. Because of their innate adaptability, malignant cells can withstand and even defy medication treatments (Ghorbian, 2025). Rather than existing as a fixed population, CSCs dynamically interconvert with non-stem cancer cells in response to environmental cues, therapeutic stress, and metabolic changes. This plasticity enables tumors to maintain cellular diversity and resilience, allowing non-stem cells to reacquire stem-like traits when conditions become unfavorable. Such reversibility challenges the traditional hierarchical model of cancer and highlights the importance of regulatory networks that sustain stemness. By activating stemness-associated transcription factors, epigenetic regulators, and survival signaling pathways, CSC plasticity promotes tumor evolution, metastatic progression, and the emergence of drug-resistant cell populations, thereby representing a major challenge for effective and durable cancer treatment (Chu et al., 2024). At the molecular level, stemness is maintained through tightly regulated transcriptional programs governed by key transcription factors such as OCT4, SOX2, NANOG, and KLF4. These factors interact with epigenetic modifiers and signalling pathways to preserve an undifferentiated state while enabling rapid adaptation to stress. Importantly, phenotypic plasticity facilitates immune evasion, metastatic colonization, and the emergence of drug-tolerant persister cells, making it a critical determinant of long-term therapeutic resistance.

#### CSCs and cellular plasticity

Cellular plasticity refers to the ability of cancer cells to dynamically transition between stem-like and non-stem cellular states in response to intrinsic and extrinsic cues. Unlike the traditional hierarchical model of tumor organization, accumulating evidence suggests that differentiated cancer cells can reacquire stem cell-like properties through processes driven by microenvironmental signals, therapeutic stress, inflammation, and EMT (Bhat et al., 2024). EMT represents a crucial biological process linking cancer stemness with therapeutic resistance. During EMT, epithelial cells lose polarity and cell–cell adhesion while acquiring mesenchymal features that enhance motility, invasiveness, and survival. Importantly, EMT is not a binary process but occurs along a spectrum, generating hybrid epithelial–mesenchymal states that are particularly enriched in stem-like properties. This bidirectional interconversion enables continuous replenishment of the CSC pool, thereby maintaining tumor heterogeneity and regenerative potential. Cellular plasticity is increasingly recognized as a major contributor to treatment failure, as surviving non-CSC populations can regenerate therapy-resistant CSCs following chemotherapy, radiotherapy, or targeted therapy, ultimately promoting tumor recurrence and metastatic progression (Al-khreisat et al., 2026).

#### CSCs and metabolic plasticity

Cells undergoing partial EMT exhibit increased resistance to apoptosis, enhanced DNA damage repair, and metabolic plasticity, all of which contribute to reduced drug sensitivity. EMT-associated transcription factors such as SNAIL, SLUG, ZEB1/2, and TWIST not only regulate cellular plasticity but also modulate drug transporter expression and anti-apoptotic signalling. Moreover, EMT facilitates immune evasion by reducing antigen presentation and altering cytokine signalling, further supporting tumor persistence during therapy. These features make EMT a central biological bridge connecting stemness, immune modulation, and resistance mechanisms.

Metabolic plasticity enables CSCs to reprogram their energy production and biosynthetic pathways according to nutrient availability, oxygen tension, and therapeutic pressure. Unlike differentiated tumor cells that often rely predominantly on aerobic glycolysis, CSCs can flexibly switch between glycolysis, oxidative phosphorylation (OXPHOS), fatty acid oxidation, glutamine metabolism, and other metabolic pathways to sustain survival and self-renewal (Masoudi et al., 2024). This metabolic adaptability allows CSCs to thrive in hypoxic and nutrient-deprived tumor microenvironments while resisting metabolic stress induced by anticancer therapies. Furthermore, metabolic plasticity contributes to drug resistance by enhancing antioxidant defenses, reducing reactive oxygen species (ROS)-mediated damage, promoting DNA repair, and maintaining cellular quiescence. Consequently, targeting metabolic vulnerabilities of CSCs has emerged as a promising strategy to overcome therapeutic resistance and improve treatment efficacy (Palm, 2021).

### Molecular pathways linking stemness and drug resistance

Cancer stemness and drug resistance are deeply interconnected processes that share common regulatory pathways. Several developmental signalling cascades, normally active during embryogenesis, are aberrantly reactivated in CSCs, driving both self-renewal and therapeutic tolerance. The Wnt/β-catenin pathway plays a central role in maintaining stemness by regulating cell fate decisions and proliferation. Aberrant activation of Wnt signalling promotes resistance to chemotherapy and radiotherapy by enhancing DNA repair mechanisms and reducing apoptotic sensitivity. Similarly, the Notch signalling pathway contributes to cell fate determination and supports CSC survival under cytotoxic stress, particularly in solid tumors such as breast, colorectal, and pancreatic cancers. The Hedgehog pathway is another critical regulator of CSC maintenance. Its persistent activation enhances tumor-initiating capacity and contributes to resistance against chemotherapeutic agents by sustaining quiescent cell populations that evade drug-induced cytotoxicity. Additionally, pathways such as PI3K/AKT/mTOR, TGF-β, and JAK/STAT integrate extracellular signals to modulate survival, metabolism, and immune evasion. Collectively, these signalling networks do not function in isolation. Instead, they form interconnected feedback loops that reinforce stem-like traits and promote adaptive resistance under therapeutic pressure.

### Tumor microenvironment and adaptive resistance

The TME plays a decisive role in sustaining cancer stemness and therapeutic resistance. Hypoxia, nutrient deprivation, acidic pH, and inflammatory cytokines collectively shape CSC behavior. Hypoxic niches, in particular, stabilize hypoxia-inducible factors (HIFs), which activate stemness-associated genes and promote resistance to radiation and chemotherapy. Tumorigenesis, development, and immune evasion are all significantly influenced by metabolic-immune interaction in the TME (Li et al., 2025c). One of the primary obstacles to successful cancer treatment is tumor heterogeneity, which is the occurrence of physiologically, epigenetically, and biologically diverse cell populations both inside and between tumors (Ibekwe et al., 2025). The development of cancer and the response to treatment are significantly influenced by the TME. It is made up of a complicated web of blood vessels, extracellular matrix, immune cells, and stromal cells that all communicate with malignant cells and affect tumor behavior (Desai et al., 2025). Patients with cancer have poor outcomes as a result of drug resistance. A possible tactic to combat drug resistance is adaptive therapy, which takes advantage of competitive interactions between resistant and susceptible sub-clones (Chen et al., 2025). Interactions with stromal cells, cancer-associated fibroblasts, immune cells, and endothelial cells further reinforce drug resistance through paracrine signalling. These interactions promote the secretion of growth factors, cytokines, and extracellular vesicles that reprogram cancer cells toward a stem-like phenotype. Such microenvironment-driven adaptations enable tumors to survive even aggressive therapeutic regimens.

Emerging evidence indicates that ncRNAs play a pivotal role in mediating bidirectional communication between CSCs and the TME. HIF-1α- and HIF-2α dependent signaling induces hypoxia-responsive miRNAs, lncRNAs, and circRNAs, which then regulate stemness-associated pathways including Wnt/β-catenin, Notch, Hedgehog, PI3K/AKT, and TGF-β signaling (Wang et al., 2025c). These hypoxia-regulated ncRNAs upregulate the expression of pluripotency-associated transcription factors including OCT4, SOX2, NANOG and BMI1, and promote EMT, metabolic reprogramming, angiogenesis and immune evasion (Guo et al., 2025). Moreover, cancer-associated fibroblasts (CAFs) remodel the extracellular matrix and secrete cytokines, chemokines and growth factors including TGF-β, IL-6, CXCL12 and hepatocyte growth factor, which activate ncRNA-mediated signaling networks promoting CSC maintenance, metastatic dissemination and resistance to chemotherapy, radiotherapy, targeted therapy and immunotherapy (Guo and Xu, 2024).

Exosome-mediated intercellular communication further enhances these adaptive responses in the TME. Extracellular vesicles, rich in miRNAs, lncRNAs, circRNAs, and to a lesser degree piRNAs, are released by tumor cells, CSCs, CAFs, mesenchymal stromal cells, immune cells, and endothelial cells and can be transferred to neighboring or distant recipient cells (Mahamed et al., 2025). These exosomal ncRNAs rewire gene expression by modulating apoptosis, autophagy, DNA damage repair, immune checkpoint signaling and metabolic adaptation, thus promoting the acquisition of stem-like phenotypes and therapy-resistant characteristics (Liu C. et al., 2025). Conversely, stromal cell exosomal ncRNAs can reprogram the behavior of tumor cells by activating pro-survival signaling pathways and inhibiting antitumor immune responses, creating a dynamic feedback loop between CSCs and TME (Li et al., 2025b). Therefore, targeting exosome-mediated ncRNA signaling or the reciprocal interactions between CSCs and their microenvironment, is emerging as a promising strategy to overcome adaptive resistance, inhibit tumor recurrence, and improve long-term clinical outcomes.

### Epigenetic regulation of stemness and drug resistance

Epigenetic plasticity is a fundamental driver of cancer stemness. Through reversible modifications such as DNA methylation, histone acetylation and methylation, chromatin remodeling, and non-coding RNA-mediated regulation, cancer cells can dynamically alter gene expression programs without changing the underlying DNA sequence. These reversible changes allow cancer cells to rapidly adapt to therapeutic stress (Galassi et al., 2025). The quantity and state of activation of transcription factors and their associated regulators, as well as other reversible changes to DNA and histones known as epigenetic marks, all play a crucial role in regulating gene expression (Galassi et al., 2025). The methylation of DNA, modification of histone, and non-coding RNAs are examples of epigenetic alterations that are essential for controlling gene expression without changing the fundamental genetic code (Yu et al., 2024). These epigenetic mechanisms govern the expression of key stemness-associated transcription factors, including OCT4, SOX2, NANOG, KLF4, and BMI1, as well as EMT regulators such as SNAIL, TWIST, and ZEB family proteins. Aberrant epigenetic reprogramming has been reported in numerous cancers, including breast, colorectal, lung, pancreatic, prostate, ovarian, glioblastoma, and hematological malignancies, where it contributes to the maintenance of CSCs populations, cellular plasticity, metastatic potential, and resistance to chemotherapy, radiotherapy, targeted therapies, and immunotherapy (Bhushan et al., 2025). Importantly, the reversible nature of epigenetic alterations enables tumor cells to rapidly adapt to therapeutic stress and environmental changes, facilitating the emergence of drug-tolerant persister cells and disease recurrence. To this end, epigenetic regulators often cooperate with transcription factors to maintain stem cell identity while suppressing differentiation programs. Importantly, epigenetic dysregulation also contributes to the persistence of drug-tolerant cell populations that can later repopulate the tumor following treatment cessation. Consequently, targeting epigenetic regulators and their associated ncRNA networks has emerged as a promising strategy for disrupting CSC maintenance and overcoming treatment resistance in cancer (Ng et al., 2025).

### Convergence of stemness and drug resistance networks

Cancer stemness and drug resistance are not independent phenomena but rather interconnected biological states driven by shared molecular networks. Signaling pathways, transcriptional regulators, metabolic rewiring, and epigenetic mechanisms collectively shape a resilient cellular phenotype capable of surviving therapeutic pressure.

NcRNAs coordinate stemness-associated transcription factors, EMT regulators, and various oncogenic signaling pathways via regulating gene expression at transcriptional, post-transcriptional, and epigenetic levels. Many miRNAs, lncRNAs, and circRNAs modulate stemness genes like OCT4, SOX2, NANOG, KLF4, BMI1, CD44, and ALDH1 while affecting drug-resistance mechanisms like apoptosis evasion, DNA damage repair, autophagy, cellular quiescence, and multidrug efflux transporter expression. Hence, ncRNAs regulate stemness, cellular plasticity, and treatment adaption as key hubs (Reis and Bassères, 2026).

The convergence of these networks is enhanced by ncRNA-mediated control of key signaling pathways, such as Wnt/β-catenin, Notch, Hedgehog, PI3K/AKT/mTOR, JAK/STAT, TGF-β, NF-κB, and Hippo/YAP Oncogenic lncRNAs including HOTAIR, MALAT1, H19, and NEAT1, as well as miRNAs like miR-21 and miR-155, activate pro-survival pathways and EMT-associated programs to maintain CSCs and resist chemotherapy, radiation, and targeted treatments (Zhong et al., 2025). However, tumor-suppressive miRNAs like miR-34, miR-200, miR-145, and the let-7 family suppress EMT and CSC-related signaling networks to reduce stemness and restore treatment sensitivity (Zhong et al., 2025). Moreover, ncRNAs form self-reinforcing feedback loops with epigenetic modifiers, chromatin remodelers, and transcriptional regulators to maintain stem-like states under treatment stress. Therefore, CSCs can survive treatment, undergo phenotypic changes, and reproduce diverse tumor populations, causing disease relapse and metastasis (Bure and Nemtsova, 2023).

Emerging research reveals that targeting individual resistance pathways may not be enough because highly integrated ncRNA-centered regulatory circuits sustain stemness and drug resistance. Therapeutic techniques that alter ncRNA-mediated networks can impair CSC maintenance, reverse EMT, overcome treatment resistance, and reduce tumor recurrence (Soragni et al., 2025). Thus, developing next-generation medicines that can sustain clinical responses in aggressive and refractory malignancies requires understanding the molecular architecture of these ncRNA-regulated networks. Understanding this convergence provides critical insight into why conventional therapies frequently fail to eradicate tumors completely. It also underscores the need for strategies that target both bulk tumor cells and the stem-like subpopulations that fuel recurrence and metastasis (Li et al., 2025a).

## A broad landscape of ncRNAs in cancer stemness, EMT and drug resistance

The most common cancer in males, prostate cancer (PC) is still a leading cause of cancer-related death globally. NcRNAs have become a major area of study in molecular biology in recent years since they are essential to the onset and progression of PC (Zhou et al., 2025). The incidence and mortality rates of acute myeloid leukemia (AML), a very variable illness, are considerably greater among the elderly. A significant percentage of nonconserved regions that are translated into long noncoding RNAs (lncRNAs) and housed in regions frequently linked to cancer can be found in the human cancer genome (Leucci, 2026). A core fraction of tumor tissues with stem cell characteristics is known as CSCs. The human genome is pervasively transcribed, yet only a small fraction encodes proteins. The vast majority of transcripts belong to the category of ncRNAs, once considered transcriptional noise but now recognized as powerful regulators of gene expression. In cancer biology, ncRNAs form a complex regulatory layer that shapes cellular identity, phenotypic plasticity, and therapeutic response. Their ability to fine-tune transcriptional programs, epigenetic states, and signaling pathways positions them at the center of cancer stemness and drug resistance (Wang H. et al., 2025). Unlike protein-coding genes, ncRNAs exert their functions through diverse molecular interactions rather than enzymatic activity. Their regulatory flexibility allows them to respond rapidly to environmental cues, cellular stress, and therapeutic interventions, making them particularly relevant in the context of tumor adaptation and survival.

### Role of miRNAs in regulating gene expression of stemness gene and EMT regulator for drug resistance

As widely known, miRNAs represent a class of small, non-coding RNA molecules that exert profound regulatory effects on gene expression (Burgos et al., 2021). In the context of cancer, miRNAs have emerged as pivotal players in modulating the behavior of CSCs, a subset of cells within tumors endowed with self-renewal and differentiation capabilities (Ying et al., 2008). MiRNAs fine-tune the delicate balance between self-renewal and differentiation in CSCs, thereby influencing their phenotypic plasticity (Kreso and Dick, 2014). By binding to the 3′untranslated region (UTR) of target messenger RNAs, miRNAs can either suppress translation or facilitate degradation of specific genes, ultimately impacting CSC behavior. Moreover, miRNAs exert their influence on key signaling pathways essential for maintaining stemness, such as the Wnt/β-Catenin, Notch, and Hedgehog pathways. Dysregulation of miRNAs has been implicated in various cancer types, underscoring their clinical relevance (Table 1). Harnessing the regulatory potential of miRNAs in cancer stemness holds promise for the development of targeted therapies aimed at disrupting the foundational mechanisms driving tumor initiation, progression, and resistance to treatment (Castro-Oropeza et al., 2018).

Cancer is a highly heterogeneous and complex disease characterized by uncontrolled cell growth and dissemination. Within tumors, a specialized subset of cells, known as CSCs, have garnered significant attention for their critical role in tumor initiation, progression, and resistance to therapy (Ratti et al., 2020). These cells, reminiscent of normal stem cells, possess the unique capacity for self-renewal and differentiation. In recent years, miRNAs, a class of small non-coding RNA molecules, have emerged as key players in the regulation of gene expression. MiRNAs are instrumental in post-transcriptional gene silencing, exerting control over a myriad of cellular processes. Their involvement in modulating CSCs behavior and characteristics has become an area of intense research. This article delves into the intricate interplay between miRNAs and cancer stemness, shedding light on the molecular mechanisms that underlie this critical aspect of tumorigenesis (Yu et al., 2012). MiRNAs are small RNA molecules, typically consisting of approximately 22 nucleotides, that play a crucial role in regulating gene expression. They operate primarily at the post-transcriptional level by binding to the 3′untranslated region (UTR) of target mRNAs. This binding either leads to mRNA degradation or translational repression, ultimately reducing the expression of the target gene. By virtue of this mechanism, miRNAs fine-tune gene expression networks, exerting control over a wide range of cellular processes, including cell proliferation, differentiation, apoptosis, and immune response. The dysregulation of miRNA expression has been implicated in various diseases, including cancer (O’Brien et al., 2018).

**FIGURE 1:**
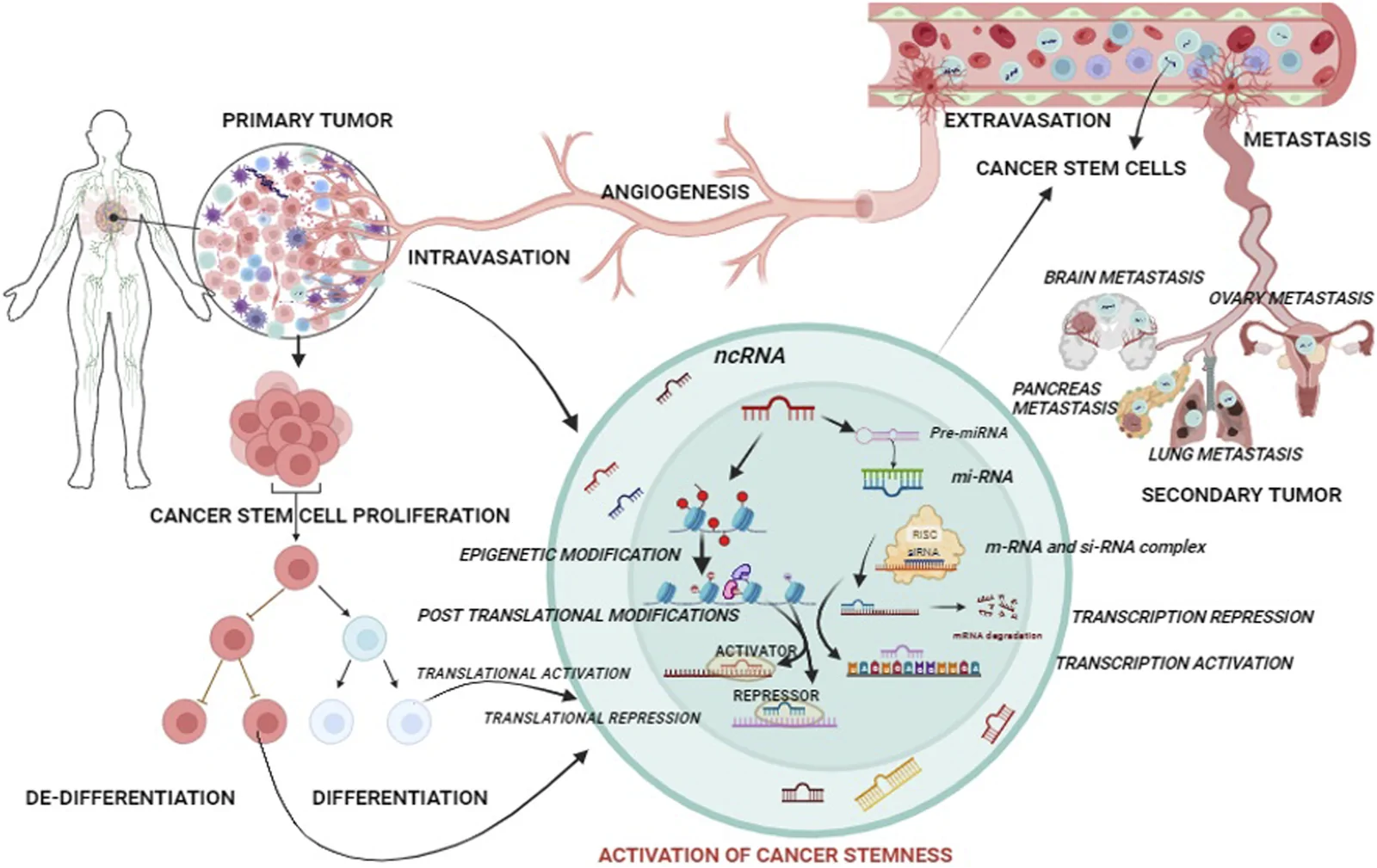
Role of non-coding RNA in cancer stemness. Non-coding RNAs modulates the behaviour of CSCs via influencing the target genes and signalling pathways. miRNAs orchestrate the balance between self-renewal and differentiation of CSCs by regulating transcription repression or activation. Non-coding RNA may influence the activation of cancer stemness at several level via epigenetic modification, post-translational modification, translational activation or repression.

Cancer stem cells represent a small subpopulation within tumors that exhibit stem cell-like properties. These cells are endowed with the ability to self-renew, generating identical copies of themselves, and differentiate, giving rise to the diverse cellular populations that constitute a tumor (O’Brien et al., 2018). This unique behavior allows CSCs to maintain the growth and perpetuation of the tumor. Importantly, CSCs have been implicated in tumor initiation, progression, metastasis, and resistance to conventional therapies. Their presence within a tumor can lead to recurrence and pose a significant challenge for successful cancer treatment (Mai et al., 2023). The role of ncRNAs, in particular, miRNAs in cancer stemness has garnered increasing attention due to their potential to modulate critical aspects of CSC behaviour (Figure 1). Through their interactions with specific target genes and signaling pathways, miRNAs influence the balance between self-renewal and differentiation in CSCs. MiRNAs have been identified that promote or inhibit self-renewal in CSCs. For instance, miR-34a, a well-studied tumor suppressor miRNA, has been shown to inhibit self-renewal and induce differentiation in CSCs by targeting key stemness-related genes. MiRNAs play a pivotal role in regulating the differentiation potential of CSCs (Bhaskaran and Mohan, 2014). MiRNAs like let-7 and miR-200 family members have been implicated in promoting differentiation and inhibiting the stem cell-like phenotype in various cancer types. EMT is a process by which cells acquire migratory and invasive properties, often associated with CSC behavior (Valinezhad Orang et al., 2014). MiRNAs like miR-200 family members have been shown to inhibit EMT, thereby suppressing CSC properties.

Understanding the role of miRNAs in regulating cancer stemness holds immense therapeutic potential. Profiling miRNA expression in patient samples can provide valuable prognostic information and guide treatment decisions. Additionally, strategies aimed at modulating specific miRNAs or targeting key signaling pathways in CSCs hold promise for the development of innovative and targeted therapeutic interventions. The interplay between miRNAs and cancer stemness represents a dynamic and promising area of research within the field of oncology. By unraveling the intricate molecular dialogues that underlie CSC behavior, researchers are poised to uncover novel therapeutic targets and strategies that may revolutionize cancer treatment, ultimately offering renewed hope for patients grappling with these formidable diseases.

### Structural and functional implications of lncRNAs in cancer stemness, EMT and therapeutic drug resistance

LncRNAs represent one of the most functionally diverse classes of regulatory RNA molecules, typically exceeding 200 nucleotides in length. Unlike protein-coding transcripts, lncRNAs exert their biological effects primarily through structural flexibility and molecular interactions rather than enzymatic activity. Their ability to interact with DNA, RNA, and proteins allows them to function as central regulators of gene expression programs involved in development, differentiation, and disease progression (Mattick et al., 2023). In cancer, lncRNAs have emerged as key determinants of cellular identity, stemness, and therapeutic responsiveness. One of the defining characteristics of lncRNAs is their context-specific expression. Many lncRNAs exhibit tissue-, developmental-, or disease-specific patterns, making them critical modulators of tumor heterogeneity. Functionally, lncRNAs operate through diverse mechanisms, including chromatin remodeling, transcriptional regulation, post-transcriptional modulation, and signal transduction. In the nucleus, lncRNAs often act as molecular scaffolds, bringing together chromatin-modifying enzymes and transcription factors to regulate gene expression. For example, certain lncRNAs recruit histone methyltransferases or deacetylases to specific genomic loci, thereby altering chromatin accessibility and shaping transcriptional landscapes that favor oncogenic transformation or stem-like phenotypes (Gao et al., 2020).

Beyond chromatin-level regulation, lncRNAs play a critical role in transcriptional control by interacting directly with transcription factors or RNA polymerase II. Through these interactions, they can either enhance or suppress transcriptional output in a context-dependent manner. This regulatory flexibility enables cancer cells to dynamically adjust gene expression in response to environmental cues such as hypoxia, nutrient limitation, or therapeutic stress. At the post-transcriptional level, lncRNAs influence mRNA stability, splicing, and translation (Statello et al., 2021). Many lncRNAs act as competing endogenous RNAs (ceRNAs), sequestering miRNAs and preventing them from repressing their target transcripts. This mechanism allows lncRNAs to indirectly regulate entire gene networks involved in cell survival, proliferation, and resistance to therapy. In cancer stem cells, such ceRNA networks are particularly important for maintaining stemness-associated gene expression programs. LncRNAs also play a central role in EMT and cellular plasticity. By modulating EMT-associated transcription factors and signaling pathways, lncRNAs facilitate phenotypic switching between epithelial and mesenchymal states. This plasticity enables tumor cells to acquire invasive traits, resist therapeutic pressure, and seed distant metastases. Importantly, lncRNA-mediated EMT is often reversible, allowing cancer cells to adapt dynamically to changing microenvironmental conditions (Singh M. et al., 2025).

In the context of drug resistance, lncRNAs regulate multiple adaptive mechanisms. These include modulation of drug efflux transporters, alteration of apoptotic signaling pathways, and activation of survival-promoting cascades such as PI3K–AKT and NF-κB. Some lncRNAs also influence DNA damage repair processes, enabling cancer cells to survive genotoxic stress induced by chemotherapy or radiotherapy. The persistence of these lncRNA-driven adaptive states contributes significantly to treatment failure and tumor recurrence. Collectively, lncRNAs serve as integrative regulatory hubs that connect epigenetic regulation, transcriptional control, and post-transcriptional modulation. Their functional versatility and disease-specific expression profiles make them promising candidates for diagnostic, prognostic, and therapeutic applications in cancer biology (Li et al., 2025a).

### Versatile role of circular RNAs

CircRNAs constitute a unique class of endogenous non-coding RNAs characterized by a covalently closed-loop structure formed through back-splicing events. This circular configuration renders circRNAs resistant to exonuclease-mediated degradation, resulting in exceptional molecular stability compared to linear RNAs. Initially regarded as transcriptional byproducts, circRNAs are now recognized as biologically active molecules with important regulatory roles in cellular homeostasis and disease progression (Wang et al., 2017). One of the most extensively studied functions of circRNAs is their ability to act as molecular sponges for miRNAs. By harboring multiple miRNA-binding sites, circRNAs can sequester miRNAs and prevent them from repressing their target mRNAs. This regulatory mechanism allows circRNAs to fine-tune gene expression networks involved in cell proliferation, differentiation, and survival. In cancer, dysregulated circRNA–miRNA interactions often lead to aberrant activation of oncogenic pathways or suppression of tumor suppressor genes (Ma et al., 2023).

Beyond miRNA sequestration, circRNAs also interact directly with RNA-binding proteins, influencing protein localization, stability, and function. Through these interactions, circRNAs can regulate key cellular processes such as transcription, RNA splicing, and signal transduction. Some circRNAs act as scaffolds that facilitate the assembly of protein complexes, thereby modulating intracellular signaling cascades critical for cancer cell survival. Emerging evidence suggests that certain circRNAs possess translational potential despite lacking canonical cap and poly(A) structures (Huang et al., 2020). Under specific conditions, such as cellular stress, circRNAs can be translated into functional peptides that contribute to tumorigenesis or stress adaptation. Although this field is still evolving, these findings highlight the multifunctional nature of circRNAs. In the context of cancer stemness, circRNAs play a pivotal role in maintaining self-renewal capacity and cellular plasticity. By regulating pathways such as Wnt/β-catenin, Notch, and Hippo, circRNAs support stem-like traits and enhance resistance to differentiation signals. Their high stability allows them to persist in harsh tumor microenvironments, contributing to long-term maintenance of malignant phenotypes (Fischer and Leung, 2017).

Recent studies have further demonstrated that circRNAs function as integral components of competing endogenous RNA (ceRNA) networks that coordinate cancer stemness, EMT, and therapeutic resistance (Pisignano et al., 2023). Acting as molecular decoys for tumor-suppressive miRNAs, circRNAs regulate the expression of stemness-associated transcription factors including OCT4, SOX2, NANOG, KLF4, BMI1, and EMT regulators such as SNAIL, TWIST, ZEB1, and ZEB2. CircRNAs including circHIPK3, circCDR1as (ciRS-7), circRNA_100290, circFOXO3, and circSMARCA5 have been implicated in modulating Wnt/β-catenin, PI3K/AKT, TGF-β, and Notch signaling pathways through diverse circRNA–miRNA–mRNA regulatory axes (Yang et al., 2026). These ceRNA networks establish self-reinforcing feedback loops that sustain CSC self-renewal, cellular plasticity, metastatic dissemination, and resistance to chemotherapy and radiotherapy. Importantly, the cancer- and tissue-specific expression patterns of circRNAs highlight their functional heterogeneity and suggest that individual circRNAs may serve as context-dependent regulators of tumor progression (Nademi and Ozfiliz-Kilbas, 2026).

In addition to their intracellular regulatory functions, circRNAs are increasingly recognized as important mediators of intercellular communication within the tumor microenvironment. CircRNAs are selectively packaged into exosomes and other extracellular vesicles, enabling their transfer between cancer cells, cancer-associated fibroblasts, immune cells, and endothelial cells (Zhang N. et al., 2025). Exosomal circRNAs can remodel the tumor microenvironment by promoting angiogenesis, immune evasion, metabolic reprogramming, and the acquisition of stem-like phenotypes in recipient cells, thereby facilitating tumor progression and drug resistance. Owing to their remarkable stability, resistance to RNase degradation, and detectability in body fluids, circulating circRNAs have emerged as promising minimally invasive biomarkers for cancer diagnosis, prognosis, and therapeutic monitoring (Liu et al., 2025b). Furthermore, advances in RNA-targeting technologies, including antisense oligonucleotides, CRISPR/Cas-based RNA editing, and nanoparticle-assisted delivery systems, have accelerated efforts to therapeutically target oncogenic circRNAs or restore tumor-suppressive circRNAs, underscoring their considerable potential in precision oncology (Singh et al., 2026).

CircRNAs also participate actively in therapeutic resistance. They modulate drug metabolism, apoptosis, and autophagy, enabling cancer cells to survive pharmacological stress. Additionally, circRNAs are frequently enriched in extracellular vesicles, facilitating intercellular communication within the tumor microenvironment. Through this mechanism, resistant cells can transmit adaptive signals to neighboring cells, amplifying resistance across the tumor mass. Given their stability, abundance, and disease-specific expression, circRNAs are increasingly explored as non-invasive biomarkers and therapeutic targets. Their functional versatility positions them as key regulators in cancer progression and treatment resistance (Hua et al., 2025).

### Functional role of piRNAs in genome stability

Piwi-interacting RNAs (piRNAs) are a class of small non-coding RNAs typically 24–32 nucleotides in length, originally characterized for their role in maintaining genomic stability in germline cells. However, growing evidence indicates that piRNAs also play significant roles in somatic tissues, including cancer. Unlike miRNAs, piRNAs associate with PIWI proteins to form complexes that regulate gene expression at both transcriptional and post-transcriptional levels. One of the primary functions of piRNAs is the suppression of transposable elements, thereby preserving genome integrity (Siomi et al., 2011). In cancer cells, dysregulation of piRNA pathways can lead to genomic instability, a hallmark of tumor progression. Altered piRNA expression has been associated with increased mutation rates, chromosomal rearrangements, and aberrant gene expression patterns that favor oncogenesis. Beyond transposon silencing, piRNAs participate in epigenetic regulation by guiding chromatin-modifying complexes to specific genomic regions. Through DNA methylation and histone modification, piRNAs influence transcriptional programs associated with cell proliferation, differentiation, and survival. These epigenetic effects are particularly relevant in cancer stem cells, where long-term maintenance of stemness requires stable yet adaptable gene expression states (Zhang et al., 2023).

Recent studies have also highlighted the involvement of piRNAs in regulating signaling pathways linked to tumor growth and metastasis. By interacting with mRNAs or regulatory proteins, piRNAs can modulate pathways such as PI3K/AKT, MAPK, and TGF-β, thereby influencing cellular behavior under stress conditions. Their contribution to drug resistance has also been increasingly recognized, particularly in relation to DNA damage repair and apoptosis avoidance. In addition, piRNAs have been implicated in shaping the tumor microenvironment. Through intercellular communication mechanisms, including extracellular vesicles, piRNAs may influence immune cell behavior and inflammatory responses, indirectly affecting tumor progression and treatment outcomes. Although the study of piRNAs in cancer is still emerging, accumulating evidence suggests that they represent an underexplored regulatory layer with significant functional relevance. Their stability, specificity, and regulatory capacity make them promising candidates for future diagnostic and therapeutic strategies (Yuan et al., 2016).

Recent investigations have identified piRNAs as important regulators of CSC maintenance and cellular plasticity through their interactions with stemness-associated transcription factors and epigenetic regulators. Aberrant expression of specific piRNAs has been associated with the regulation of pluripotency genes, including *OCT4*, *SOX2*, *NANOG*, and *KLF4*, thereby influencing CSC self-renewal, differentiation, and tumor-initiating capacity (Zhang J. et al., 2025). Furthermore, piRNA–PIWI complexes regulate chromatin accessibility and transcriptional activity by recruiting DNA methyltransferases and histone-modifying enzymes to target loci, resulting in stable epigenetic alterations that sustain stem-like phenotypes. These findings suggest that piRNAs serve as an additional regulatory layer linking genome stability, epigenetic reprogramming, and cancer stemness (Zhang C. et al., 2026).

Emerging evidence also indicates that piRNAs participate in complex regulatory networks involving other classes of non-coding RNAs. Crosstalk between piRNAs, microRNAs (miRNAs), long non-coding RNAs (lncRNAs), and circular RNAs (circRNAs) contributes to the fine-tuning of gene expression networks governing epithelial–mesenchymal transition (EMT), metastatic progression, and therapeutic resistance (Cai et al., 2026). Through modulation of key signaling pathways such as PI3K/AKT/mTOR, Wnt/β-catenin, STAT3, TGF-β, and NF-κB, piRNAs influence cell proliferation, apoptosis, DNA damage repair, and metabolic adaptation under therapeutic stress (Jiang et al., 2025). These interconnected regulatory circuits enable CSCs to survive chemotherapy, radiotherapy, targeted therapies, and immunotherapy, thereby contributing to tumor relapse and disease progression (Lee et al., 2025).

The remarkable stability of piRNAs in tissues and body fluids has also attracted considerable interest for their translational potential. Aberrant piRNA expression signatures have been reported in multiple malignancies, including breast, gastric, colorectal, lung, liver, and prostate cancers, where they correlate with tumor stage, metastatic potential, patient prognosis, and treatment response (Zhang C. et al., 2026). Consequently, circulating piRNAs are being actively investigated as minimally invasive biomarkers for early cancer detection, disease monitoring, and prediction of therapeutic outcomes (Zafar et al., 2025). In addition, advances in RNA-based therapeutics and targeted delivery platforms have created new opportunities to modulate oncogenic or tumor-suppressive piRNAs using antisense oligonucleotides, synthetic piRNA mimics, and nanoparticle-assisted delivery systems (Singh M. et al., 2025). Although clinical translation remains at an early stage, continued characterization of piRNA-mediated regulatory networks is expected to facilitate the development of novel diagnostic and therapeutic strategies targeting cancer stemness and drug resistance.

### Cancer-specific ceRNA axes

Competing endogenous RNA (ceRNA) networks have been identified as a key regulatory circuit by which lncRNAs and circRNAs regulate cancer stemness, EMT, and therapeutic resistance. These ncRNAs act as molecular sponges to sequester tumor suppressive miRNAs, thereby relieving repression of downstream mRNA targets involved in stem cell maintenance, proliferation and survival (El-Ashmawy et al., 2025). These ceRNA interactions generate multi-level regulatory networks that incorporate transcriptional, posttranscriptional and epigenetic mechanisms to ensure plasticity of CSCs. An increasing body of evidence suggests that dysregulation of ceRNA axes promotes tumor progression by simultaneously activating stemness-associated transcription factors, EMT regulators, and oncogenic signaling pathways (Aria et al., 2024).

Among the well-characterized ceRNA circuits that are important for maintaining CSC characteristics is the HOTAIR/miR-34/OCT4 axis. The oncogenic lncRNA HOTAIR acts as a molecular sponge of miR-34, relieving the repression of *OCT4*, one of the master transcription factors for pluripotency and self-renewal (Nademi and Ozfiliz-Kilbas, 2026). This axis upregulation promotes CSC maintenance, tumor-initiating capacity, and resistance to chemo- and radiotherapy. Similarly, the MALAT1/miR-200/ZEB1 regulatory loop promotes EMT by sponging miR-200 family members, leading to upregulation of *ZEB1* and inhibition of epithelial differentiation (Bitaraf et al., 2025). Activation of this pathway promotes cellular plasticity, metastatic spread and acquisition of stem-like phenotypes, ultimately contributing to disease progression and therapeutic resistance (Al-khreisat et al., 2026).

Additional ceRNA networks further demonstrate the complexity of ncRNA-mediated regulation in cancer. The H19/let-7/HMGA2 axis keeps CSCs stemness and induces their proliferation via opposing the tumor-suppressive let-7 family, resulting in the upregulation of the chromatin-associated protein HMGA2, a key regulator of stemness and EMT (Xia et al., 2024). Likewise, NEAT1 facilitates EMT and metastatic progression through the NEAT1/miR-204/ZEB1 axis, and the circHIPK3/miR-124/STAT3 loop triggers STAT3 signaling to promote CSC self-renewal, inflammatory signaling, and drug resistance (Alshahrani et al., 2024). Other representative examples are circCDR1as (ciRS-7) that acts as a potent sponge of miR-7 leading to upregulation of oncogenic targets such as *EGFR*, *CCNE1* and *PIK3CD* resulting in increased proliferation and resistance to targeted therapies (Paasch et al., 2025).

**FIGURE 2:**
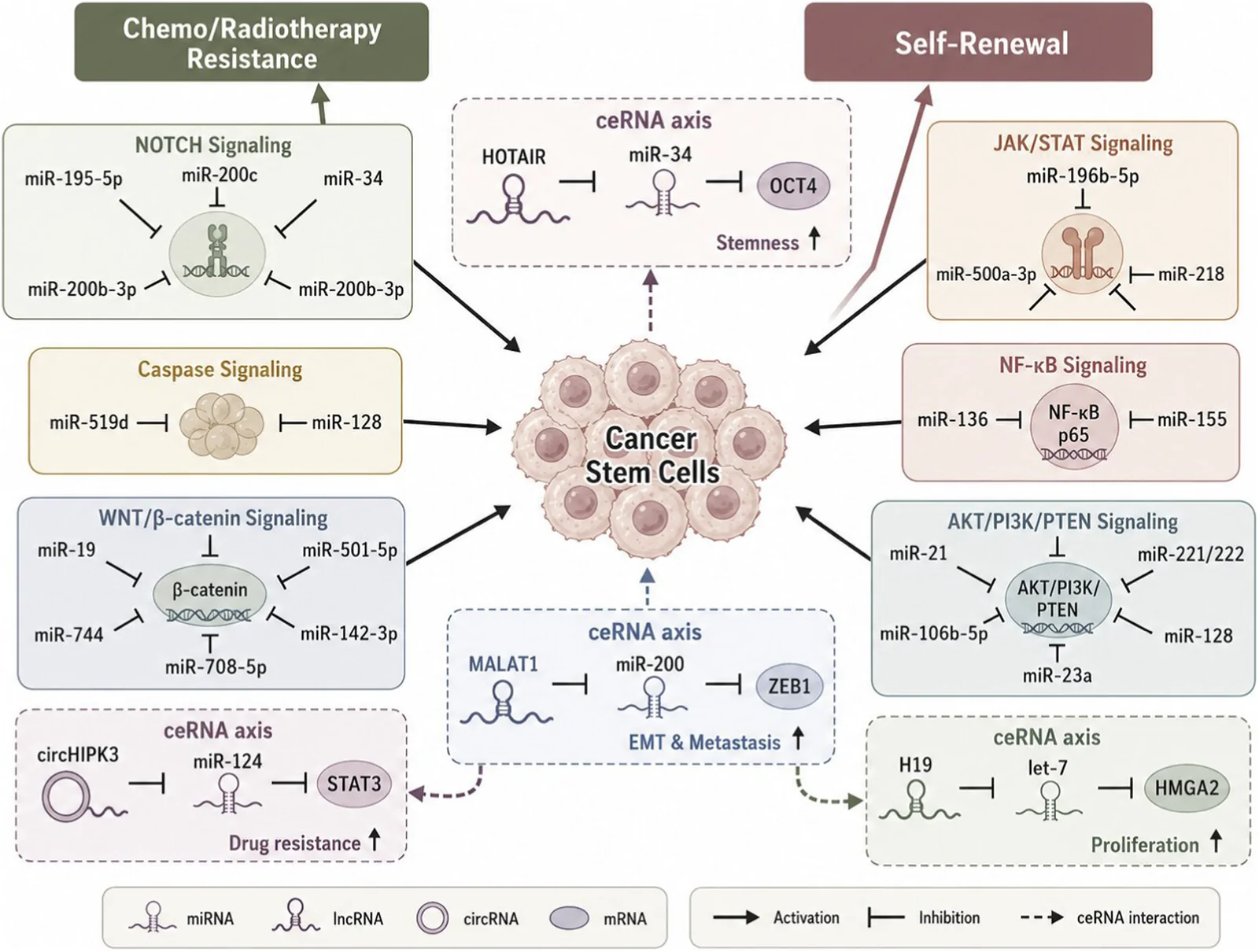
An integrated network of different ncRNAs, stemness genes and EMT regulators in cancer drug resistance and other outcomes. This schematic illustrates the complex regulatory network through which non-coding RNAs (ncRNAs), including long non-coding RNAs (lncRNAs), microRNAs (miRNAs), and circular RNAs (circRNAs), orchestrate cancer stemness, epithelial–mesenchymal transition (EMT), and therapy resistance. Representative ncRNAs such as HOTAIR, MALAT1, H19, NEAT1, UCA1, NORAD, SNHG16, ANRIL, miR-34a, miR-200c, miR-145, miR-29b, let-7 family, miR-21, miR-27a, miR-125b, circCDR1as, circHIPK3, and circSMARCA5 are shown regulating key stemness-associated transcription factors and markers, including OCT4, SOX2, NANOG, KLF4, BMI1, CD44, and ALDH1A1. These stemness regulators interact with EMT-inducing transcription factors such as SNAIL, SLUG, TWIST1, ZEB1, and ZEB2, thereby promoting cellular plasticity, self-renewal capacity, invasion, and metastatic dissemination.

Collectively, these representative ceRNA regulatory circuits exemplify the co-regulation of interconnected molecular networks by lncRNAs and circRNAs that integrate stemness-associated TFs, EMT regulators, and key oncogenic signaling pathways such as Wnt/β-catenin, PI3K/AKT, STAT3, Notch, TGF-β, and NF-κB (Alkan and Cansaran-Duman, 2025) (Figure 2). These ceRNA networks are not isolated but form highly interconnected regulatory hubs, allowing CSCs to dynamically adapt to therapeutic stress, evade apoptosis, and sustain long-term tumor growth (Lee et al., 2025). Thus, targeting crucial ceRNA interactions represents a promising therapeutic strategy to simultaneously target cancer stemness, metastatic progression and treatment resistance, opening new avenues for the development of ncRNA-based precision oncology (Pandey and Yadav, 2025).

### Functional role of other small non-coding RNAs

Beyond miRNAs and piRNAs, several other classes of small non-coding RNAs contribute to the regulation of cellular homeostasis and cancer progression. These include small nucleolar RNAs (snoRNAs), transfer RNA–derived fragments (tRFs), small nuclear RNAs (snRNAs), and enhancer RNAs (eRNAs). Although historically considered housekeeping molecules, many of these RNAs have now been recognized as active participants in gene regulatory networks. Small nucleolar RNAs primarily guide chemical modifications of ribosomal RNAs, such as methylation and pseudouridylation. However, dysregulation of snoRNAs has been linked to altered ribosome biogenesis, translational fidelity, and cellular stress responses (Gou et al., 2023). In cancer, aberrant snoRNA expression can promote uncontrolled protein synthesis and support rapid tumor growth. Transfer RNA–derived fragments have emerged as versatile regulators of gene expression. Generated through precise cleavage of mature or precursor tRNAs, tRFs can regulate translation, RNA stability, and stress responses. In cancer cells, tRFs often accumulate under conditions of oxidative stress or nutrient deprivation, enabling adaptive translational reprogramming. Some tRFs also interact with Argonaute proteins, functioning in a miRNA-like manner to regulate gene expression (Zhang D. et al., 2019).

Small nuclear RNAs play essential roles in pre-mRNA splicing. Alterations in snRNA expression or function can lead to aberrant splicing events, generating oncogenic isoforms or disrupting tumor suppressor gene expression. Splicing dysregulation is increasingly recognized as a driver of cancer progression and therapeutic resistance. Enhancer RNAs, transcribed from active enhancer regions, contribute to transcriptional activation by stabilizing enhancer–promoter interactions (Wang et al., 2025e). In cancer, aberrant enhancer activity driven by eRNAs can amplify oncogene expression and reinforce malignant transcriptional programs. Collectively, these small non-coding RNAs form an intricate regulatory network that fine-tunes gene expression at multiple levels. Their coordinated activity supports cancer cell adaptability, survival, and resistance to therapy. Understanding the functional interplay among these RNA species offers valuable insights into tumor biology and opens new avenues for targeted therapeutic intervention (Wang Q. et al., 2026).

## Regulatory networks of ncRNAs in cancer stemness, EMT and drug resistance

Different classes of ncRNAs such as microRNAs, lncRNAs and circRNAs exert diverse regulatory effects on genome stability and gene expression at transcriptional, post-transcriptional and post-translational levels. NcRNAs modulate the expression and activity of crucial stemness-related genes, EMT regulators, and signaling pathways involved in cancer progression and therapy resistance through complex regulatory networks.

### MiRNA network as the major player in regulating genes implicated in stemness, EMT and drug resistance

Several miRNAs have been identified as key regulators of CSC properties, influencing self-renewal, differentiation, and therapy resistance. Regulatory networks in CSCs constitute a complex interplay of molecular interactions that dictate the unique behavior and characteristics of these specialized cells within tumors (Visvader and Lindeman, 2012). These networks are orchestrated by a multitude of signaling pathways, transcription factors, and non-coding RNAs, working in concert to maintain the stemness phenotype. Central to these networks are key signaling pathways like Wnt, Notch, and Hedgehog, which play critical roles in governing self-renewal, differentiation, and survival of CSCs. Dysregulation or aberrant activation of these pathways can lead to an imbalance in CSC behavior, potentially driving tumor progression and therapy resistance (Paul et al., 2022). In addition to signaling pathways, transcription factors also play a pivotal role in CSC regulatory networks. These master regulators control the expression of genes involved in self-renewal and differentiation processes (Ali Hosseini Rad et al., 2013). Transcription factors like OCT4, SOX2, and NANOG, which are typically associated with embryonic stem cells, have been identified in CSCs across various cancer types. Their aberrant expression or activity contributes to the maintenance of stemness and is often associated with aggressive tumor phenotypes (Li et al., 2021). Moreover, non-coding RNAs, particularly miRNAs and long non-coding RNAs, intricately weave into these networks, fine-tuning gene expression post-transcriptionally and epigenetically. Together, these regulatory elements form a sophisticated network that governs the behavior and fate of CSCs, ultimately influencing tumor progression and clinical outcomes.

### Non-coding RNA regulation of stemness-associated gene networks and signaling pathways

Recent advancements have elucidated how lncRNAs and microRNAs coordinate the activation of core developmental cascades, including the WNT/β-catenin, PI3K/AKT/mTOR, and JAK/STAT3 pathways, to preserve the self-renewal capacity and tumorigenic potential of cancer stem cells (Chu et al., 2024). These regulatory circuits frequently operate by sequestering miRNAs or serving as scaffolding platforms for RNA-binding proteins that modulate the stability of key transcripts (Zeng et al., 2023). Furthermore, circular RNAs function as endogenous competitive sponges to fine-tune these signaling axes by sequestering specific miRNAs, thereby preventing the inhibition of downstream stemness-associated effectors. Beyond these buffering roles, specific lncRNAs such as HOTTIP serve as epigenetic scaffolds that activate transcription factors like HOXA9, promoting the stabilization of stemness profiles through targeted gene expression changes. In addition to epigenetic modulation, these transcripts exert influence through the titration of microRNAs, such as the mechanism by which DLX6-AS1 modulates the availability of miR-129-5p to upregulate DLK1 and foster a pluripotent state. Furthermore, clinical investigations into hematological malignancies have identified lncRNA DANCR as a critical facilitator of stem cell renewal, where its silencing directly induces cellular quiescence in leukemic stem cell populations. Furthermore, the emergence of the ceRNA framework reveals that long non-coding RNAs can act as molecular decoys, neutralizing inhibitory miRNAs that otherwise suppress crucial pluripotency factors like OCT4, NANOG, and SOX2. These interactions often culminate in the assembly of reciprocal feedback loops, as exemplified by the recruitment of EZH2 by specific lncRNAs to refine the epigenetic landscape and further augment the tumor-initiating capacity. These regulatory networks also encompass the modulation of protein stability, such as the interaction between lncRNA PVT1 and KLF5, which facilitates β-catenin signaling and drives the development of aggressive tumor phenotypes. Concurrently, the silencing of lncRNA MT1JP has been shown to disrupt the miRNA-214/RUNX3 axis, effectively impairing the expression of pro-apoptotic genes like Bim and enhancing the overall survival of these malignant cell populations. An integrated network of ncRNAs (miRNAs, lncRNAs, circRNAs) together with cancer stemness genes (*OCT4*, *SOX2*, *NANOG*, *KLF4*, *BMI1*, *CD44*, *ALDH1*) and EMT regulators (SNAIL, SLUG, TWIST, ZEB1/2) and their relationship with different signaling pathways (Wnt, Notch, Hedgehog, PI3K/AKT, JAK/STAT, Hippo) (Ouyang et al., 2020) and therapy resistance outcomes (chemoresistance, radioresistance, immune evasion, recurrence) have been presented in Figure 2.

## Main miRNA-mediated signaling pathways

**FIGURE 3:**
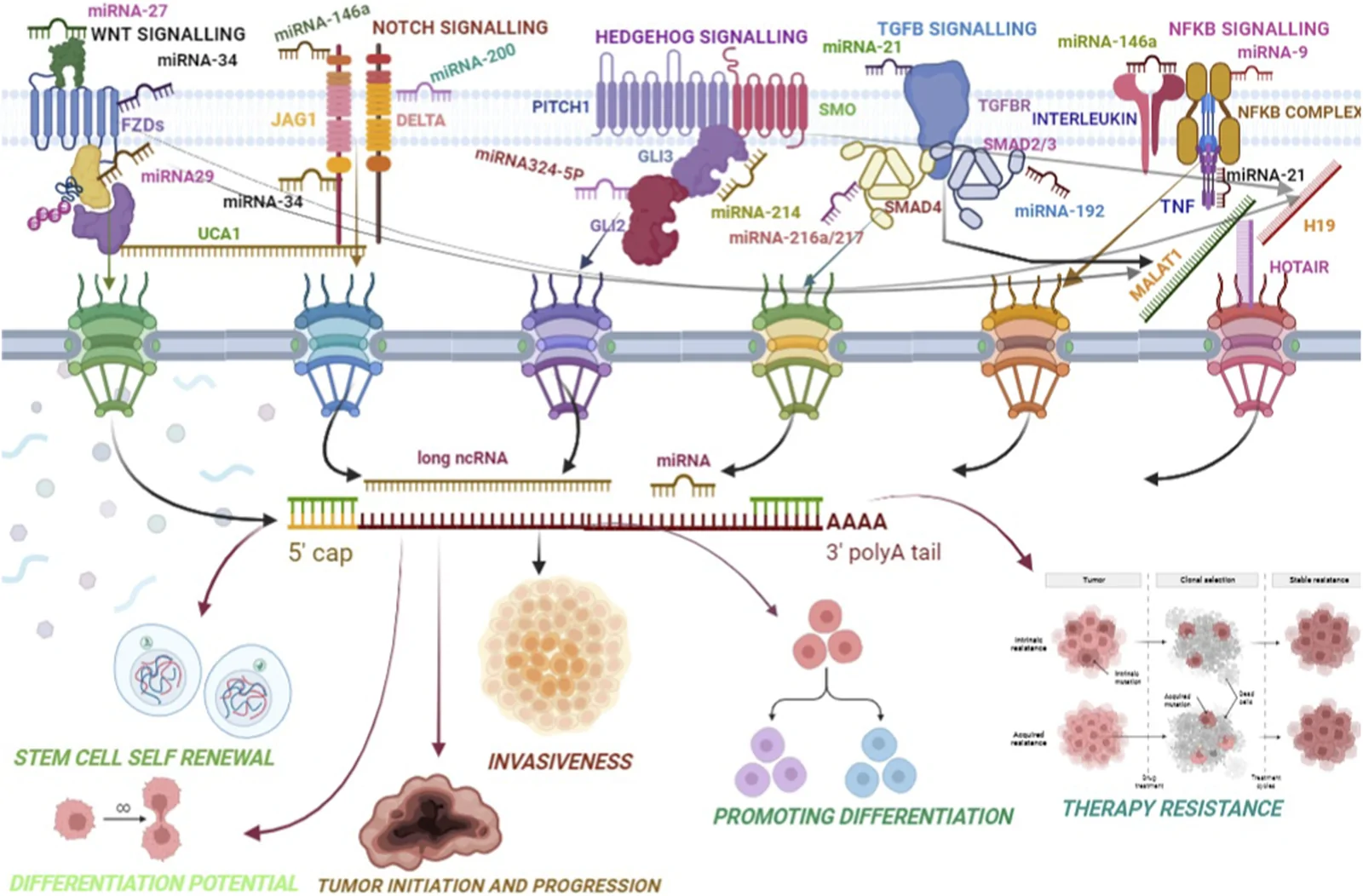
miRNAs as a key player in governing cancer stem cell behaviour. Several miRNAs have been reported to modulate the critical signalling pathways that govern the CSCs behaviour including chemo/radiotherapy resistance, self-renewal, metastasis as well as cell-proliferation and differentiation. miRNAs involved in regulating different signalling pathways are depicted in the figure.

To start, miRNAs modulate critical signaling pathways involved in stemness, including Wnt, Notch, and Hedgehog, thereby influencing CSC behavior. miRNAs are small, non-coding RNA molecules that play a crucial role in post-transcriptional regulation of gene expression. Within the context of CSCs, miRNAs have emerged as key players in modulating critical signaling pathways that govern CSC behaviour (Figure 3). MiRNAs are small, non-coding RNA molecules that play a pivotal role in post-transcriptional gene regulation. By binding to mRNAs, miRNAs modulate gene expression, influencing various cellular processes. Importantly, miRNAs have been found to be integral components of signaling pathways, orchestrating a wide array of cellular responses (Yu et al., 2012).

### Wnt/β-catenin pathway

The Wnt/β-catenin pathway is a pivotal regulator of stem cell maintenance and self-renewal. MiRNAs can directly target components of this pathway, including Wnt ligands, receptors, and downstream effectors. Dysregulation of miRNAs involved in Wnt signaling can lead to uncontrolled self-renewal, a hallmark of CSC behavior. The Wnt/β-Catenin pathway is fundamental for cellular development, tissue homeostasis, and stem cell regulation. Dysregulation of this pathway is associated with numerous diseases, including cancer. miRNAs such as miR-34a and miR-200 have been identified as potent regulators of Wnt signaling, exerting their influence by targeting key components within the pathway (Peter, 2009; Lamouille et al., 2014). The Wnt/β-Catenin signaling pathway is a critical cellular cascade involved in development, tissue homeostasis, and stem cell regulation. Dysregulation of this pathway is a hallmark of cancer, contributing to tumor initiation, progression, and therapy resistance. Recent research has illuminated the significant role of miRNAs in modulating the Wnt/β-Catenin pathway in the context of cancer stemness.

#### miR-34 family: the master regulator

The miR-34 family (miR-34a, miR-34b, and miR-34c) is a well-known tumor suppressor miRNA group that targets various components of the Wnt/β-Catenin pathway. The miR-34 family acts as a master tumor-suppressive regulator of CSCs by inhibiting self-renewal and stemness-associated signaling pathways, particularly Notch, Wnt/β-catenin, and CD44-mediated signaling (Sadeghi et al., 2025). These miRNAs directly inhibit Wnt ligands, Frizzled receptors, and downstream effectors like β-Catenin, thus negatively regulating pathway activation. In colorectal cancer stem cells, miR-34a has been shown to suppress self-renewal and promote differentiation by inhibiting Wnt signaling (Zhang L. et al., 2019). Furthermore, miR-34 family members suppress EMT and enhance therapeutic sensitivity, thereby reducing tumor recurrence and drug resistance.

#### miR-29 family: inhibits ECM remodeling, EMT and stemness

The miR-29 family functions as a tumor-suppressive regulator of cancer stemness by inhibiting self-renewal and EMT through targeting genes involved in stemness, extracellular matrix remodeling, and oncogenic signaling pathways (Shinde et al., 2024). The miR-29 family plays a crucial role in modulating Wnt/β-Catenin signaling by targeting multiple components, including Wnt ligands and downstream effectors like TCF7L2 (Zeng et al., 2023). In hepatocellular carcinoma stem cells, miR-29a/b/c have been implicated in inhibiting self-renewal and promoting differentiation through suppression of Wnt signaling (Hadjimichael et al., 2015). Additionally, miR-29 family members enhance therapeutic sensitivity by suppressing CSC maintenance and reversing drug-resistant phenotypes.

#### miR-27a/b as an important promoter of CSC self-renewal

The miR-27a/b has been identified as key regulators of Wnt/β-Catenin signaling by targeting the Frizzled receptor family members. By downregulating these receptors, miR-27a/b negatively impact Wnt pathway activation (Mamun et al., 2020). In breast cancer stem cells, miR-27a has been found to inhibit self-renewal and induce differentiation by suppressing Wnt signalling (Castro et al., 2013). The regulation of the Wnt/β-Catenin pathway by miRNAs has profound implications for cancer stemness. Dysregulated Wnt signaling is associated with the acquisition and maintenance of stem cell-like properties in cancer cells, including self-renewal, differentiation potential, and resistance to therapy. By targeting key components of this pathway, miRNAs exert a profound influence on these stemness characteristics, ultimately impacting the behavior of CSCs. The regulatory role of miRNAs in the Wnt/β-Catenin pathway represents a critical aspect of the molecular landscape governing cancer stemness. Through their precise targeting of key components within this pathway, miRNAs exert a profound influence on the behavior of CSCs. Understanding this intricate interplay offers valuable insights into the underlying mechanisms driving tumor initiation, progression, and therapy resistance, ultimately paving the way for innovative therapeutic strategies targeting CSCs (Wu et al., 2025).

### Notch signaling

Notch signaling is crucial for maintaining stemness and promoting the survival and proliferation of CSCs. MiRNAs exert their influence on this pathway by targeting key components such as Notch receptors, ligands, and downstream effectors. Dysregulation of miRNAs involved in Notch signaling can lead to aberrant stem cell behavior and contribute to tumor aggressiveness. The Notch pathway is critical for cell fate determination, differentiation, and tissue patterning. Dysregulated Notch signaling has been implicated in various cancers. miRNAs like miR-34 and miR-200 have been found to target components of the Notch pathway, influencing cellular differentiation and proliferation. The Notch signaling pathway is a pivotal cellular cascade involved in cell fate determination, differentiation, and tissue homeostasis. Dysregulation of this pathway is a hallmark of cancer, contributing to tumor initiation, progression, and therapy resistance. Recent research has illuminated the significant role of miRNAs in modulating the Notch pathway in the context of cancer stemness (Howe et al., 2011).

#### miR-34 family potent tumor-suppressive regulator of stemness

The miR-34 family, known for its tumor suppressor role, has been identified as a regulator of Notch signaling. miR-34 directly targets the 3′-UTR of Notch receptors and downstream effectors, leading to pathway inhibition. In glioblastoma stem-like cells, miR-34a has been shown to inhibit self-renewal and promote differentiation by suppressing Notch signalling (Shi et al., 2015). The miR-34 family functions as a potent tumor-suppressive regulator of cancer stemness by inhibiting key stemness-associated genes and signaling pathways, including NOTCH, CD44, BCL-2, and Wnt/β-catenin. Through suppression of CSC self-renewal, EMT, and survival signaling, miR-34 family members enhance therapeutic sensitivity and limit tumor recurrence (Sadeghi et al., 2025).

#### miR-146a suppresses CSC self-renewal

The miR-146a is known for its anti-inflammatory properties, but it also plays a role in regulating Notch signaling. It targets Notch1 and Notch2 receptors, thereby modulating pathway activity. In breast cancer stem cells, miR-146a has been implicated in inhibiting self-renewal and promoting differentiation through suppression of Notch signalling (Xie et al., 2014). Furthermore, miR-146a has been demonstrated to suppress Notch signaling in breast cancer stem cells, thus inhibiting CSC maintenance and stimulating cellular differentiation (Xie et al., 2014). This reduced Notch activity results in downregulation of stemness-related genes and perturbed signaling networks that promote tumor initiation and progression. In addition to its function in stemness, miR-146a has been shown to be involved in the regulation of EMT, inflammatory signaling, and treatment sensitivity. Since Notch signaling is closely associated with CSC maintenance and drug resistance, restoration of miR-146a expression may be a promising therapeutic strategy to suppress the stemness-associated phenotypes, enhance the treatment responsiveness and reduce the tumor recurrence (Shi et al., 2024). Collectively, these observations underscore miR-146a as an important tumor suppressive miRNA that connects inflammation, regulation of stemness, and therapeutic resistance in cancer.

#### miR-200 family as potent suppressor of EMT and stemness

The miR-200 family members, including miR-200a, miR-200b, miR-200c, miR-141, and miR-429, have been identified as regulators of Notch signaling. These miRNAs target the 3′UTR of Jagged1 and Delta-like ligands, key Notch ligands. In pancreatic cancer stem cells, miR-200 family members have been found to inhibit self-renewal and promote differentiation by suppressing Notch signalling (Li et al., 2010). The regulation of Notch signaling by miRNAs has profound implications for cancer stemness. Dysregulated Notch signaling is associated with the acquisition and maintenance of stem cell-like properties in cancer cells, including self-renewal, differentiation potential, and resistance to therapy. By targeting key components of this pathway, miRNAs exert a profound influence on these stemness characteristics, ultimately impacting the behavior of CSCs. The regulatory role of miRNAs in the Notch signaling pathway represents a critical aspect of the molecular landscape governing cancer stemness. Through their precise targeting of key components within this pathway, miRNAs exert a profound influence on the behavior of CSCs. Understanding this intricate interplay offers valuable insights into the underlying mechanisms driving tumor initiation, progression, and therapy resistance, ultimately paving the way for innovative therapeutic strategies targeting CSCs (Ghafouri-Fard et al., 2021).

### Hedgehog signaling

The Hedgehog pathway is implicated in CSC self-renewal and tumor progression. MiRNAs can modulate the activity of key components within this pathway, influencing the stemness properties of CSCs. The Hedgehog pathway plays a crucial role in embryonic development, tissue repair, and cancer. Aberrant activation of this pathway is associated with numerous malignancies. miRNAs such as miR-324-5p and miR-214 have been identified as key regulators of Hedgehog signaling, impacting processes like cell survival and tissue regeneration (Ji et al., 2009). The Hedgehog signaling pathway plays a crucial role in embryonic development, tissue regeneration, and stem cell maintenance. Dysregulation of this pathway has been implicated in various cancers, including those with a stem cell component. Recent research has shed light on the role of miRNAs in modulating Hedgehog signaling in the context of cancer stemness. This note explores the intricate interplay between miRNAs and the Hedgehog pathway, highlighting their collective influence on the behavior of CSCs (Guessous et al., 2013).

#### miR-324-5p: tumor-suppressive miRNA and regulator on stemness through Hedgehog signaling

The miR-324-5p has been identified as a potent regulator of Hedgehog signaling by directly targeting components of the pathway. It inhibits the expression of Gli2, a crucial transcription factor downstream of Hedgehog signaling. In pancreatic cancer stem cells, miR-324-5p has been found to suppress self-renewal and induce differentiation by inhibiting Hedgehog signalling (Guessous et al., 2013). Downregulation of Hedgehog pathway activity reduces the expression of stemness-associated genes and disrupts the signaling networks required for CSC maintenance and tumor propagation. Furthermore, aberrant activation of Hedgehog signaling has been linked to EMT, chemoresistance, and metastatic progression in several cancer types (Cong et al., 2025). By antagonizing this pathway, miR-324-5p may contribute to the reversal of stem-like phenotypes and enhance therapeutic sensitivity. These findings suggest that miR-324-5p functions as a tumor-suppressive miRNA and represents a potential therapeutic target for disrupting Hedgehog-driven CSC populations and overcoming treatment resistance in aggressive cancers (Parkins et al., 2023).

#### miR-214 promotes CSC self-renewal and therapy resistance

The miR-214 is another miRNA implicated in regulating Hedgehog signaling. It targets Gli3, a transcriptional repressor in the Hedgehog pathway. In colorectal cancer stem cells, miR-214 has been shown to inhibit self-renewal and promote differentiation through suppression of Hedgehog signalling (Lu et al., 2010). The regulation of Hedgehog signaling by miRNAs has significant implications for cancer stemness. Dysregulated Hedgehog signaling is associated with the acquisition and maintenance of stem cell-like properties in cancer cells, including self-renewal, differentiation potential, and resistance to therapy. By targeting key components of this pathway, miRNAs exert a profound influence on these stemness characteristics, ultimately impacting the behaviour of CSCs. The regulatory role of miRNAs in the Hedgehog signaling pathway represents a critical aspect of the molecular landscape governing cancer stemness. Through their precise targeting of key components within this pathway, miRNAs exert a profound influence on the behavior of CSCs. Understanding this intricate interplay offers valuable insights into the underlying mechanisms driving tumor initiation, progression, and therapy resistance, ultimately paving the way for innovative therapeutic strategies targeting CSCs (Jing et al., 2023).

### TGF-β signaling

Transforming Growth Factor-beta (TGF-β) signaling pathway is implicated in CSC maintenance and tumor progression. MiRNAs can target components of the TGF-β pathway, influencing CSC behavior and contributing to tumor aggressiveness. Transforming Growth Factor-beta (TGF-β) signaling is pivotal for cellular differentiation, immune response, and tissue homeostasis. Dysregulated TGF-β signaling is implicated in various diseases, including fibrosis and cancer. miRNAs such as miR-21 and miR-192 have been shown to modulate TGF-β signaling, influencing processes like EMT and fibrotic responses (Saba et al., 2012). The TGF-β signaling pathway is a critical cellular cascade involved in various processes, including cell growth, differentiation, and immune regulation. Dysregulation of this pathway is a hallmark of cancer, contributing to tumor initiation, progression, and therapy resistance. Recent research has highlighted the significant role of miRNAs in modulating TGF-β signaling in the context of cancer stemness (Deng et al., 2024).

#### miR-21: widely known oncomiR that acts through TGF- β signaling

miR-21 is one of the most extensively studied oncogenic microRNAs (oncomiRs) and is frequently overexpressed in a wide range of cancers. It contributes to tumor progression by regulating multiple signaling pathways involved in proliferation, survival, invasion, and therapeutic resistance. miR-21 has been shown to modulate TGF-β signaling through targeting TGF-β receptor II (TGFBR2), thereby influencing downstream pathways associated with cancer stemness and EMT (Wang C. et al., 2025). In breast cancer stem cells, elevated miR-21 expression promotes self-renewal, survival, and therapy resistance, partly through dysregulation of TGF-β signaling (Deng et al., 2024). These findings highlight miR-21 as a key regulator of CSC maintenance and a potential therapeutic target for overcoming treatment resistance.

#### miR-192: tumor suppression through TGF-β/Smad3 signaling

miR-192 functions as a tumor-suppressive microRNA and plays an important role in regulating the TGF-β signaling pathway, which is closely associated with cancer stemness, epithelial–mesenchymal transition (EMT), and therapeutic resistance. It exerts its effects by targeting Smad3, a key downstream mediator of TGF-β signaling, thereby attenuating the transcriptional programs involved in stem cell maintenance and tumor progression (Yang et al., 2025). In liver cancer stem cells, miR-192 has been shown to inhibit self-renewal and promote cellular differentiation through suppression of TGF-β/Smad3 signaling (Cao et al., 2015). By limiting TGF-β pathway activity, miR-192 reduces stemness-associated traits and may contribute to increased sensitivity to anticancer therapies, highlighting its potential as a therapeutic regulator of cancer stem cell plasticity and drug resistance.

#### miR-216a/217: tumor-suppressive miRNAs

The miR-216a/217 has been identified as regulators of TGF-β signaling by targeting Smad7, a negative regulator of the pathway. In glioblastoma stem-like cells, miR-216a/217 have been shown to inhibit self-renewal and promote differentiation by suppressing TGF-β signaling. The regulation of TGF-β signaling by miRNAs has profound implications for cancer stemness. Dysregulated TGF-β signaling is associated with the acquisition and maintenance of stem cell-like properties in cancer cells, including self-renewal, differentiation potential, and resistance to therapy. By targeting key components of this pathway, miRNAs exert a profound influence on these stemness characteristics, ultimately impacting the behavior of CSCs. The regulatory role of miRNAs in the TGF-β signaling pathway represents a critical aspect of the molecular landscape governing cancer stemness. Through their precise targeting of key components within this pathway, miRNAs exert a profound influence on the behavior of CSCs. Understanding this intricate interplay offers valuable insights into the underlying mechanisms driving tumor initiation, progression, and therapy resistance, ultimately paving the way for innovative therapeutic strategies targeting CSCs (Zhang et al., 2012).

### NF-κB pathway: the central regulator of CSC maintenance, self-renewal, survival, and stemness-associated gene expression

Recent studies have identified miRNAs as important modulators of NF-κB signaling in the regulation of cancer stemness. Several oncogenic miRNAs, including miR-21, miR-155, and miR-221/222, enhance NF-κB pathway activity by suppressing negative regulators of the signaling cascade, thereby promoting CSC self-renewal, EMT, and drug resistance (Guo et al., 2024). Conversely, tumor-suppressive miRNAs such as miR-146a and miR-34a inhibit NF-κB activation and attenuate stemness-associated phenotypes. Through these regulatory interactions, miRNAs establish intricate feedback loops with NF-κB signaling, influencing the expression of stemness-related genes and cellular responses to therapeutic stress. Consequently, targeting miRNA–NF-κB regulatory networks represents a promising strategy for disrupting CSC maintenance and overcoming therapy resistance in cancer (Wang et al., 2024).

#### miR-146a: a tumor-suppressive regulator of cancer stemness

The miR-146a is a well-studied miRNA known for its regulatory role in the NF-κB pathway. It acts as a negative feedback regulator, targeting key components like interleukin-1 receptor-associated kinase 1 (IRAK1) and TNF receptor-associated factor 6 (TRAF6). In the context of cancer stemness, miR-146a has been shown to inhibit the NF-κB pathway in breast cancer stem cells, suppressing their self-renewal capacity and invasiveness (Dai et al., 2017). Reduced NF-κB activity subsequently decreases the expression of genes involved in CSC maintenance, inflammation, EMT, and resistance to apoptosis. Furthermore, loss or downregulation of miR-146a has been associated with enhanced tumor aggressiveness and treatment resistance in several cancer types. These findings highlight miR-146a as a key regulator linking inflammatory signaling to cancer stemness and suggest that restoration of miR-146a expression may represent a promising strategy for targeting CSC populations and overcoming therapy resistance (Guo et al., 2024).

#### miR-9: role in CSC self-renewal and survival through suppressing PTEN

The miR-9 is another miRNA known for its involvement in regulating NF-κB signaling. It targets NF-κB1, an essential subunit of the NF-κB complex. In glioblastoma stem-like cells, miR-9 has been found to downregulate NF-κB1 expression, consequently inhibiting the self-renewal and tumorigenic potential of these cells (Yamakuchi et al., 2008). Reduced NF-κB activity limits the expression of genes involved in CSC maintenance, proliferation, and survival, thereby impairing the ability of stem-like cells to sustain tumor growth. Furthermore, suppression of NF-κB signaling by miR-9 may contribute to increased sensitivity to anticancer therapies by reducing pro-survival and anti-apoptotic signaling pathways (Rinkenbaugh and Baldwin, 2016). These findings suggest that miR-9 functions as a tumor-suppressive miRNA and plays an important role in controlling NF-κB-driven stemness and therapeutic resistance in aggressive cancers such as glioblastoma. The most significant role of miR-19 is to promote CSC self-renewal and survival by suppressing the tumor suppressor PTEN, leading to activation of the PI3K/AKT signaling pathway (Carrà et al., 2022).

#### miR-21: the main player of maintenance of self-renewal of CSCs

**FIGURE 4:**
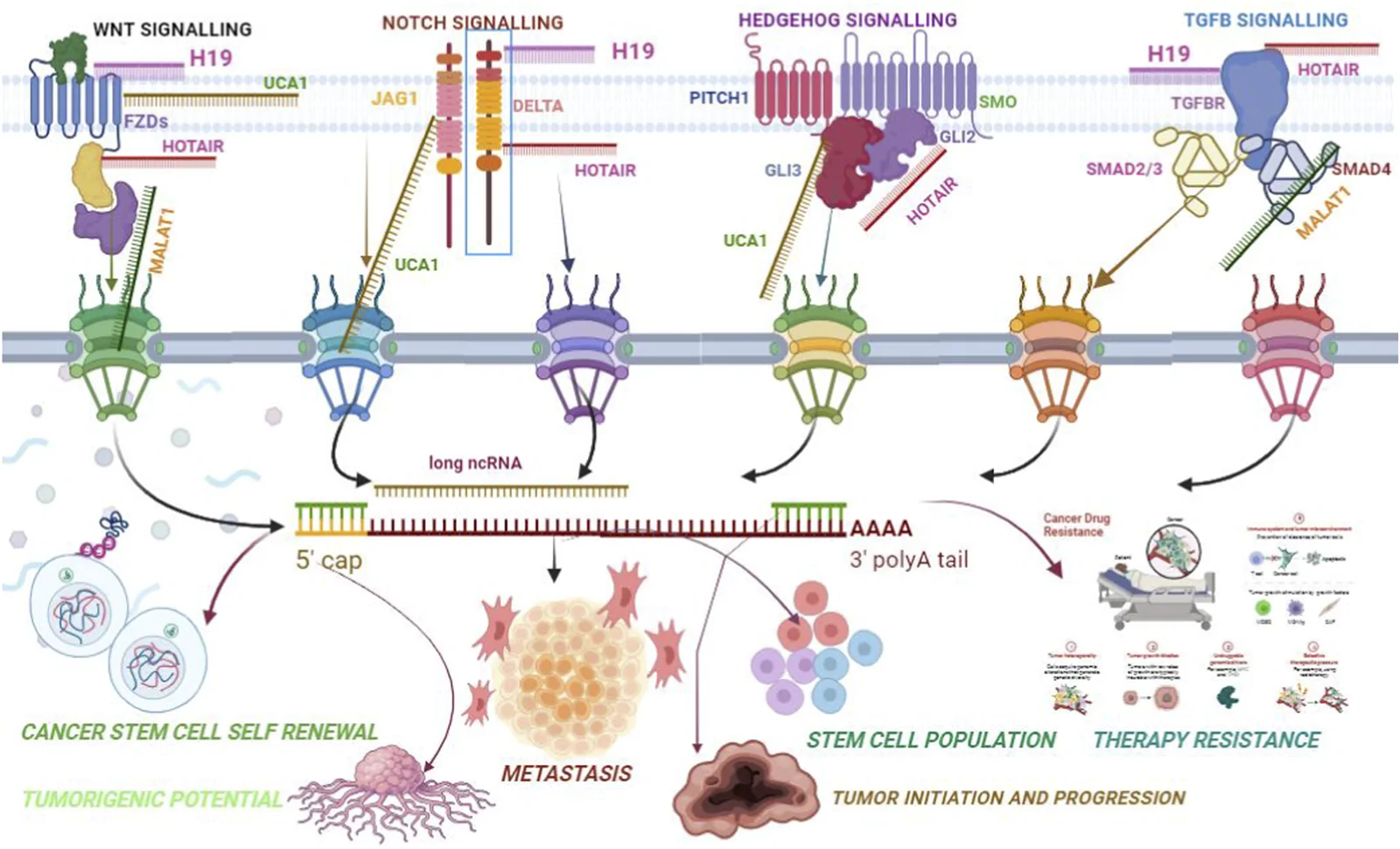
Different signaling pathways and associated miRNAs in regulating cancer stemness. Various signalling pathways like Wnt signalling, NOTCH signalling, Hedgehog Signalling, TGFB signalling, and NF-KB signalling are involved in regulating the cancer stemness behaviour.

The miR-21 is a well-documented oncomiR that has been implicated in various aspects of cancer progression. It targets multiple negative regulators of the NF-κB pathway, leading to its activation. In colorectal cancer stem cells, miR-21 has been shown to promote self-renewal and resistance to therapy through activation of the NF-κB pathway. The interplay between miRNAs and the NF-κB pathway has significant implications for cancer stemness. Dysregulated NF-κB signaling is associated with the acquisition and maintenance of stem cell-like properties in cancer cells, including self-renewal, differentiation potential, and resistance to therapy. By targeting key components of the NF-κB pathway, miRNAs exert a profound influence on these stemness characteristics, ultimately impacting the behavior of CSCs. The regulatory role of miRNAs in the NF-κB pathway represents a critical aspect of the molecular landscape governing cancer stemness. Through their precise targeting of key components within this pathway, miRNAs exert a profound influence on the behavior of CSCs. Understanding this intricate interplay offers valuable insights into the underlying mechanisms driving tumor initiation, progression, and therapy resistance, ultimately paving the way for innovative therapeutic strategies targeting CSCs. Figure 4 summarise the role of different miRNA in regulating the signalling pathway involved in the cancer stemness (Farasati Far et al., 2023).

## Long non-coding RNAs, cancer stemness and drug resistance

LncRNAs are a diverse group of non-protein coding transcripts with a length exceeding 200 nucleotides. Over the past decade, research has unveiled the critical roles of lncRNAs in various biological processes, including cancer progression. Recently, lncRNAs have emerged as key regulators in cancer stemness, a phenomenon associated with tumor initiation, progression, and resistance to therapy. This note delves into the intricate interplay between lncRNAs and cancer stemness, highlighting their potential as novel therapeutic targets in cancer treatment (Gao et al., 2020).

### LncRNA H19 and its role in cancer stemness and drug resistance

The H19, a maternally imprinted gene, is an extensively studied lncRNA in the context of cancer stemness. It has been implicated in various malignancies, including breast cancer and colorectal cancer. H19 promotes stem-like properties by modulating pathways such as Notch, Wnt/β-catenin, and TGF-β signalling. H19, a paternally imprinted lncRNA, has garnered significant attention due to its multifaceted roles in various biological processes, including cancer progression. Emerging evidence suggests that H19 plays a pivotal role in regulating cancer stemness, a phenomenon central to tumor initiation, progression, and resistance to therapy (Xia et al., 2024). H19 is an evolutionarily conserved lncRNA that plays crucial roles in normal development, imprinting regulation, and cellular homeostasis. Aberrant expression of H19 is associated with several malignancies, and its involvement in tumor progression is increasingly recognized. In breast cancer, H19 has been implicated in regulating the population of CSCs. Studies have shown that H19 promotes self-renewal and tumor-initiating capabilities of breast cancer cells, contributing to tumor aggressiveness. H19 is upregulated in colorectal cancer and has been associated with the maintenance of stem-like properties in cancer cells. It influences signaling pathways such as Wnt/β-catenin, enhancing the self-renewal capacity of colorectal CSCs. In Hepatocellular Carcinoma (HCC), H19 plays a critical role in promoting stemness characteristics. It interacts with various regulatory molecules and signaling pathways, including miR-675, leading to the expansion of the CSC population and enhanced tumorigenic potential (Liao et al., 2023).

The regulatory role of H19 in cancer stemness holds profound implications for tumor progression. By influencing key signaling pathways and molecular regulators, H19 contributes to the acquisition and maintenance of stem cell-like properties in cancer cells. This ultimately drives increased tumor-initiating potential, metastasis, and resistance to therapy. H19 emerges as a pivotal player in the intricate network governing cancer stemness. Its multifaceted roles in regulating CSC populations across various cancer types highlight its potential as a promising therapeutic target. Understanding the interplay between H19 and cancer stemness offers valuable insights into the underlying mechanisms driving tumor initiation, progression, and therapy resistance (Xia et al., 2024).

### HOTAIR and its involvement in different cancer types

The HOTAIR is a well-known lncRNA that has been linked to cancer stemness in multiple cancer types, including breast, lung, and pancreatic cancer. It exerts its influence by epigenetically silencing tumor-suppressor genes and modulating signaling pathways like Wnt and Hedgehog. HOTAIR (HOX Transcript Antisense Intergenic RNA) is a well-known lncRNA with pivotal roles in various biological processes, including cancer progression. Emerging evidence suggests that HOTAIR plays a crucial role in regulating cancer stemness, a phenomenon central to tumor initiation, progression, and resistance to therapy. HOTAIR is a lncRNA transcribed from the HOXC gene cluster on chromosome 12. It is a key player in gene regulation, acting as a scaffold for chromatin-modifying complexes, such as polycomb repressive complex 2 (PRC2) and LSD1/CoREST/REST, to modulate gene expression. HOTAIR is upregulated in breast cancer and has been linked to the maintenance and expansion of the CSC population. Studies suggest that HOTAIR promotes self-renewal and tumorigenicity of breast cancer stem cells (Liu et al., 2015). In colorectal cancer, HOTAIR plays a critical role in enhancing the stemness properties of cancer cells. It does so by modulating pathways such as Wnt/β-catenin and Notch, contributing to increased CSC populations. In gastric cancer, HOTAIR is associated with the promotion of stem cell-like properties. It influences signaling pathways such as Hedgehog and TGF-β, leading to the expansion of the CSC population and enhanced tumorigenic potential (Yang et al., 2013).

The regulatory role of HOTAIR in cancer stemness holds profound implications for tumor progression. By influencing key signaling pathways and molecular regulators, HOTAIR contributes to the acquisition and maintenance of stem cell-like properties in cancer cells. This ultimately drives increased tumor-initiating potential, metastasis, and resistance to therapy. HOTAIR emerges as a pivotal player in the intricate network governing cancer stemness. Its multifaceted roles in regulating CSC populations across various cancer types highlight its potential as a promising therapeutic target. Understanding the interplay between HOTAIR and cancer stemness offers valuable insights into the underlying mechanisms driving tumor initiation, progression, and therapy resistance (Nazari et al., 2024).

### MALAT1 and its role in promotion of self-renewal

The MALAT1, also known as NEAT2, is a widely studied lncRNA associated with cancer stemness. It plays a role in promoting self-renewal, proliferation, and metastasis of cancer stem cells by influencing pathways such as TGF-β, STAT3, and Wnt/β-catenin signalling. MALAT1 (Metastasis-Associated Lung Adenocarcinoma Transcript 1) is a lncRNA that has gained prominence due to its diverse roles in various biological processes, including cancer progression. Recent studies have unveiled its significant role in regulating cancer stemness, a phenomenon central to tumor initiation, progression, and resistance to therapy. MALAT1, also known as NEAT2, is a highly conserved lncRNA with an essential role in regulating gene expression and cellular processes. Originally identified in lung cancer, it has since been implicated in multiple cancers. In lung cancer, MALAT1 has been associated with the maintenance of stem-like properties in cancer cells. It regulates signaling pathways such as Wnt/β-catenin and TGF-β, contributing to the expansion of the CSCs population (Gupta et al., 2010). MALAT1 plays a crucial role in promoting stemness characteristics in HCC. It interacts with various regulatory molecules and signaling pathways, including the miR-204-3p/SOX4 axis, leading to the expansion of the CSC population and enhanced tumorigenic potential. In breast cancer, MALAT1 is implicated in the regulation of CSCs populations. It influences signaling pathways such as STAT3, enhancing the self-renewal capacity of breast cancer stem cells (Lu et al., 2022).

The regulatory role of MALAT1 in cancer stemness holds profound implications for tumor progression. By influencing key signaling pathways and molecular regulators, MALAT1 contributes to the acquisition and maintenance of stem cell-like properties in cancer cells. This ultimately drives increased tumor-initiating potential, metastasis, and resistance to therapy. MALAT1 emerges as a pivotal player in the intricate network governing cancer stemness. Its multifaceted roles in regulating CSC populations across various cancer types highlight its potential as a promising therapeutic target. Understanding the interplay between MALAT1 and cancer stemness offers valuable insights into the underlying mechanisms driving tumor initiation, progression, and therapy resistance (Tripathi et al., 2013; Ji et al., 2014).

### UCA1’s role in cancer stemness

Urothelial carcinoma-associated 1 (UCA1) is a lncRNA that has been implicated in promoting stemness in various cancers, including bladder, breast, and gastric cancer. It exerts its influence through multiple mechanisms, including the activation of Wnt/β-catenin and Notch signaling pathways. UCA1 has garnered significant attention due to its diverse roles in various biological processes, including cancer progression (Ghafouri-Fard and Taheri, 2019). Emerging evidence suggests that UCA1 plays a crucial role in regulating cancer stemness, a phenomenon central to tumor initiation, progression, and resistance to therapy. UCA1 is a lncRNA initially identified in bladder cancer, but its involvement has since been demonstrated in various other malignancies. It exerts its regulatory effects through diverse mechanisms, including miRNA sponging and modulation of signaling pathways. UCA1 is upregulated in bladder cancer and has been associated with the promotion of stem cell-like properties. It influences signaling pathways such as Wnt/β-catenin and Notch, contributing to increased CSC populations (Lu et al., 2022). In breast cancer, UCA1 has been implicated in the regulation of CSC populations. It influences signaling pathways such as Hedgehog and Wnt/β-catenin, enhancing the self-renewal capacity of breast cancer stem cells. UCA1 is associated with the promotion of stem cell-like properties in gastric cancer. It interacts with various regulatory molecules and signaling pathways, including the miR-193a-3p/SOX2 axis, leading to the expansion of the CSC population and enhanced tumorigenic potential (Wang et al., 2015).

The regulatory role of UCA1 in cancer stemness holds profound implications for tumor progression. By influencing key signaling pathways and molecular regulators, UCA1 contributes to the acquisition and maintenance of stem cell-like properties in cancer cells. This ultimately drives increased tumor-initiating potential, metastasis, and resistance to therapy. UCA1 emerges as a pivotal player in the intricate network governing cancer stemness (Anil et al., 2022). Its multifaceted roles in regulating CSC populations across various cancer types highlight its potential as a promising therapeutic target. Understanding the interplay between UCA1 and cancer stemness offers valuable insights into the underlying mechanisms driving tumor initiation, progression, and therapy resistance. The regulatory role of lncRNAs in cancer stemness has profound implications for tumor progression. Dysregulated stem cell-like properties are associated with increased tumor initiation, metastasis, and resistance to therapy. By modulating key signaling pathways and epigenetic regulators, lncRNAs influence the acquisition and maintenance of stemness characteristics in cancer cells. lncRNAs represent a new Frontier in our understanding of cancer stemness regulation. Their diverse mechanisms of action, including modulation of signaling pathways and epigenetic regulation, make them promising targets for novel therapeutic strategies. Understanding the intricate interplay between lncRNAs and cancer stemness offers valuable insights into the underlying mechanisms driving tumor initiation, progression, and therapy resistance.

### Interactions with transcription factors

The intricate interplay between ncRNAs and transcription factors (TFs) has emerged as a critical regulatory axis in cancer stemness. NcRNAs, including miRNAs and lncRNAs, orchestrate gene expression by interacting with TFs, thus exerting profound effects on the acquisition and maintenance of stem cell-like properties in cancer cells. This section delves into the dynamic interactions between ncRNAs and TFs, shedding light on their collective influence on cancer stemness. MiRNAs often target TFs, thereby influencing the expression of genes critical for stemness. For instance, miR-200 family members target ZEB1 and ZEB2, repressing EMT and enhancing the stemness properties in breast cancer cells (Godlewski et al., 2010). Similarly, miR-34a modulates the TF c-Myc, impacting self-renewal and differentiation in cancer stem cells. Complex regulatory loops exist, where miRNAs and TFs reciprocally regulate each other. In glioma stem cells, miR-128 targets BMI1, a TF essential for self-renewal, while BMI1, in turn, suppresses miR-128 expression, establishing a feedback loop (Mariner et al., 2008).

LncRNAs can act as scaffolds, guiding TFs to specific genomic loci. For example, lncRNA HOTAIR recruits PRC2 complex to silence tumor suppressor genes through interaction with the TF SUZ12. This epigenetic regulation contributes to the maintenance of stemness properties. LncRNAs can form complexes with TFs, modulating their activity. In prostate cancer, lncRNA PCAT-1 interacts with the TF c-Myc to promote proliferation and stemness (Bhan and Mandal, 2015). This exemplifies how lncRNAs dynamically influence TF-mediated gene regulation. The interplay between ncRNAs and TFs in cancer stemness has profound implications for tumor progression. Dysregulation of these interactions leads to aberrant gene expression patterns, culminating in the acquisition and maintenance of stem cell-like properties in cancer cells. This, in turn, drives increased tumor-initiating potential, metastasis, and resistance to therapy. The interactions between ncRNAs and TFs represent a critical aspect of the molecular landscape governing cancer stemness. Through their precise targeting of TFs and dynamic modulation of gene expression, ncRNAs exert a profound influence on the behavior of cancer stem cells. Understanding this intricate interplay offers valuable insights into the underlying mechanisms driving tumor initiation, progression, and therapy resistance, ultimately paving the way for innovative therapeutic strategies targeting cancer stemness. lncRNAs can act as scaffolds or decoys, interacting with transcription factors to fine-tune the transcriptional landscape of CSCs (Krützfeldt et al., 2007).

## Epigenetic modifications and ncRNAs-mediated regulation of cancer stemness, EMT and drug resistance

**FIGURE 5:**
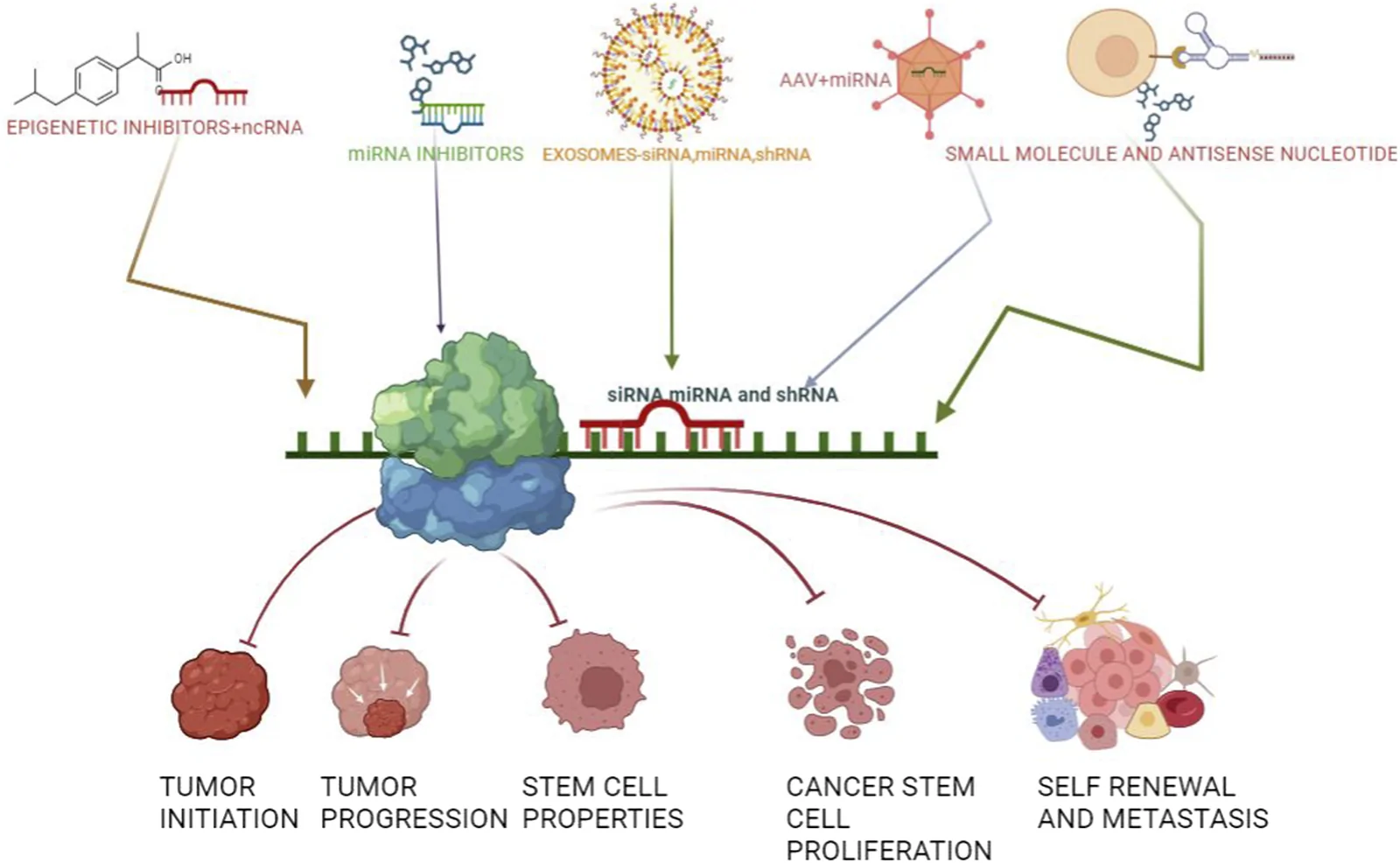
Epigenetic modifiers and non-coding RNAs in regulation of cancer stemness. Several epigenetic modifiers and non-coding RNA play an important role in gene regulation and thus regulate cancer stemness via regulating gene expression.

Epigenetic modifications and ncRNAs represent two fundamental layers of gene regulation in cells. The interplay between these regulatory mechanisms has gained immense significance in understanding cellular function and its dysregulation in various diseases, particularly cancer. In this section we will explores the complex relationship between epigenetic modifications and ncRNA-mediated regulation, shedding light on their combined impact on gene expression and cancer stemness (Figure 5). Epigenetic modifications involve heritable changes in gene expression without alterations in the underlying DNA sequence. Key mechanisms include DNA methylation, histone modifications, and chromatin remodeling. These modifications play pivotal roles in cellular differentiation, development, and disease. Non-coding RNAs, including miRNAs and lncRNAs, constitute a diverse class of RNA molecules that do code for proteins. They act as post-transcriptional regulators by targeting mRNAs for degradation or translational inhibition (miRNAs) or by influencing chromatin structure and gene expression (lncRNAs) (Tachiwana and Saitoh, 2021).

### Epigenetic modifiers: ncRNAs as architects

Epigenetic modifications play a pivotal role in gene regulation, influencing cellular development, differentiation, and disease. Within this intricate landscape, ncRNAs emerge as key players in orchestrating epigenetic changes. This section delves into the fascinating realm of epigenetic modifiers, highlighting how ncRNAs act as architects, guiding and shaping the epigenetic landscape. Epigenetic modifications involve heritable alterations in gene expression without changes to the underlying DNA sequence. Key mechanisms include DNA methylation, histone modifications, and chromatin remodeling. These modifications are critical for regulating gene accessibility and expression. Non-coding RNAs, a diverse class of RNA molecules, do not code for proteins but are crucial regulators of gene expression. They include miRNAs, lncRNAs, and others. NcRNAs exert their influence at various levels, from chromatin structure to post-transcriptional regulation. MiRNAs play a significant role in modulating DNA methylation patterns. For example, miR-29 family members target DNA methyltransferases (DNMTs), influencing DNA methylation dynamics. LncRNAs like HOTAIR have been shown to interact with DNMTs, guiding them to specific genomic loci, ultimately impacting DNA methylation patterns (Iorio et al., 2005).

### DNA promoter methylation and stemness regulation

DNA methylation is a key epigenetic mechanism that regulates gene expression through the addition of methyl groups to cytosine residues within CpG-rich promoter regions. Aberrant promoter methylation contributes to cancer stemness by silencing tumor suppressor genes and activating stemness-associated pathways (Dhar et al., 2021). Hypermethylation-mediated suppression of differentiation-associated genes and hypomethylation-induced activation of pluripotency factors such as OCT4, SOX2, and NANOG promote CSC maintenance, self-renewal, and therapeutic resistance. Altered DNA methylation patterns have also been implicated in EMT induction and the acquisition of aggressive phenotypes across multiple cancer types (Sergeeva et al., 2023).

Non-coding RNAs, in particular miRNAs, can target histone-modifying enzymes, influencing histone acetylation and methylation states. This dynamic regulation contributes to alterations in chromatin structure. LncRNAs such as XIST are involved in recruiting chromatin-modifying complexes, thereby orchestrating histone modifications and gene silencing events. DNA methylation patterns can significantly influence the expression of ncRNAs. For instance, hypermethylation of miRNA promoter regions can lead to their downregulation in various diseases, including cancer.

### Histone modifications and chromatin remodeling

Histone modifications, including acetylation, methylation, phosphorylation, and ubiquitination, play critical roles in regulating chromatin accessibility and transcriptional activity. Dysregulation of histone-modifying enzymes such as histone deacetylases (HDACs), histone acetyltransferases (HATs), and histone methyltransferases contributes to CSC maintenance and EMT progression (Carlberg, 2024).

NcRNAs, acting as architectural guides, direct epigenetic machinery to specific genomic locations, ultimately shaping gene expression patterns. Histone modifications, through their impact on chromatin accessibility, play a crucial role in regulating the expression of ncRNAs. Acetylation of histones in the promoter regions of lncRNAs can enhance their transcription. The interplay between epigenetic modifiers and ncRNAs is an attractive area of research in the field of molecular biology. Chromatin remodeling complexes further influence the expression of stemness-associated genes and signaling pathways, including Wnt/β-catenin, Notch, and Hedgehog. These epigenetic alterations facilitate cellular plasticity, tumor adaptation, and resistance to chemotherapy and radiotherapy (Rouault et al., 2025). Understanding this intricate interplay holds significant promise for unraveling the molecular mechanisms underlying development, disease, and potential therapeutic interventions.

### Epigenetic changes in ncRNA expression and non-coding RNA-mediated epigenetic regulation of cancer stemness

This is widely acknowledged that ncRNAs, including miRNAs, lncRNAs, and circRNAs, function as important epigenetic regulators by interacting with DNA methyltransferases, histone-modifying enzymes, chromatin remodeling complexes, and transcription factors (Mangraviti and Castelli, 2025). Through these interactions, ncRNAs can modulate promoter methylation, alter histone marks, and regulate the expression of stemness- and EMT-related genes. Furthermore, ncRNAs participate in complex feedback loops with epigenetic regulators to control key signaling pathways involved in CSC maintenance, metastasis, and drug resistance. These findings highlight ncRNAs as central mediators linking epigenetic regulation to cancer stemness and therapeutic response (Singh M. et al., 2025).

Epigenetic modifications, which involve heritable changes in gene expression without alterations in the underlying DNA sequence, play a pivotal role in cellular regulation. Within this dynamic landscape, ncRNAs emerge as influential regulators of gene expression of stemness genes and EMT regulators. This section explores the intriguing realm of epigenetic changes in ncRNA expression, shedding light on their collective impact on cellular function and disease. Epigenetic modifications encompass various molecular alterations, including DNA methylation, histone modifications, and chromatin remodeling. These modifications are crucial for regulating gene accessibility, transcription, and ultimately, cellular behavior. Non-coding RNAs, a diverse class of RNA molecules, do not code for proteins but exert profound regulatory effects on gene expression. This class includes miRNAs, lncRNAs, and others. NcRNAs function at multiple levels, from chromatin structure to post-transcriptional regulation. Understanding this intricate interplay holds great promise for unraveling the molecular mechanisms underlying health and disease, paving the way for innovative therapeutic interventions (Murakami et al., 2006). Their combined influence on gene expression offers a deeper understanding of the molecular mechanisms underlying health and disease (Meng et al., 2007). Deciphering this intricate interplay holds great promise for the development of innovative therapeutic strategies targeting epigenetic and ncRNA-based pathways.

## Therapeutic and clinical implications

The cancer- and pathway-specific expression patterns of a number of ncRNAs provide opportunities for their diagnostic and prognostic biomarker and therapeutic target use. Thus, extensive efforts are being made to develop ncRNA-based strategies to interfere with the maintenance of cCSCs and to overcome treatment resistance. Understanding the therapeutic and clinical implications of ncRNA-regulated stemness networks may pave the way for the development of more effective and durable treatment strategies for aggressive and refractory cancers (Dai et al., 2020).

### Therapeutic targeting of ncRNAs regulated stemness and EMT networks

There is significant interest in developing ncRNA-based therapeutic interventions due to the pivotal role of ncRNAs in regulating cancer stemness and therapeutic resistance. Several approaches have arisen for direct modulation of oncogenic or tumor suppressive ncRNAs involved in the maintenance of CSCs. Antisense oligonucleotides (ASOs) and siRNAs have emerged as promising strategies to suppress oncogenic lncRNAs like HOTAIR, MALAT1, H19, and UCA1, which are known to promote stemness-associated transcription factors including OCT4, SOX2, NANOG, and BMI1(Nemeth et al., 2024). Similarly, restoration of tumor-suppressive microRNAs by miRNA mimics, particularly members of the miR-34, miR-200, and let-7 families, has shown the ability to inhibit self-renewal, reverse EMT, and enhance chemosensitivity in multiple cancer models. Recent developments in CRISPR/Cas-mediated genome and epigenome editing technologies have further extended opportunities for selective targeting of ncRNA loci and stemness-associated regulatory circuits. At the same time, nanoparticle-mediated delivery systems, lipid nanoparticles, and exosome-based carriers are being developed to increase the stability, specificity, and tumor-targeted delivery of ncRNA therapeutics while reducing systemic toxicity (Pandey and Yadav, 2025).

In addition to directly targeting ncRNAs, therapeutic strategies that target downstream stemness-associated signaling pathways have also shown significant promise. Aberrant activation of Wnt/β-catenin, Notch, Hedgehog, PI3K/AKT/mTOR, JAK/STAT, NF-κB, and Hippo/YAP signaling pathways are frequently driven by ncRNA-mediated regulatory networks and contribute to CSC maintenance, tumor recurrence, and treatment failure (Hossam Abdelmonem et al., 2025). As a consequence, a number of small-molecule inhibitors and monoclonal antibodies that target these pathways are now being evaluated preclinically and clinically. Despite promising results, major obstacles exist before ncRNA-directed therapies can be widely adopted in clinical practice. These are tumor heterogeneity, off-target effects, limited delivery efficiency, immune-related adverse events, and the dynamic nature of the ncRNA-mediated regulatory networks. Future therapeutic strategies will likely incorporate combinatorial approaches combining ncRNA-targeting agents with conventional chemotherapy, targeted therapies, immunotherapy, or radiotherapy in an effort to achieve durable suppression of CSC populations and overcome therapy resistance. These strategies have great potential to enhance therapy response and prevent cancer recurrence in many types of cancers (Kaya et al., 2024).

### Epigenetic modifications as emerging avenue for therapeutic interventions

The intricate interplay between ncRNAs and epigenetic modifications has opened new avenues for therapeutic interventions. Understanding the regulatory roles of ncRNAs in modulating gene expression through epigenetic mechanisms holds significant promise for the development of targeted therapies in various diseases. This section explores the therapeutic implications of ncRNA-mediated epigenetic regulation, highlighting its potential in clinical applications. Utilizing miRNA mimics or inhibitors to restore or inhibit specific miRNAs that are dysregulated in cancer can potentially reestablish normal cellular functions. For instance, miR-34-based therapies are being explored for their tumor-suppressive effects in various malignancies. Small molecules or antisense oligonucleotides designed to target specific lncRNAs, such as MALAT1 or HOTAIR, are being investigated as potential therapeutic agents to inhibit tumor progression and metastasis (Pallante et al., 2006). Drugs targeting DNMTs and HDACs can reverse aberrant epigenetic modifications, potentially restoring normal ncRNA expression profile. This approach is particularly relevant in cancers with global DNA hypomethylation and histone deacetylation. Small molecules targeting proteins involved in epigenetic modifications, such as EZH2 or LSD1, are being developed to modulate the expression of specific ncRNAs. This approach holds promise for precise epigenetic modulation in various diseases. Utilizing nanoparticle-based delivery systems allows for targeted and controlled release of miRNA or lncRNA therapeutics. These nanoparticles can protect the ncRNAs from degradation and enhance their cellular uptake, potentially improving therapeutic efficacy. Profiling patient-specific ncRNA expression patterns can guide the selection of tailored therapeutic strategies. This approach holds potential for optimizing treatment outcomes by targeting the specific ncRNA dysregulations driving disease progression (Calin et al., 2005).

While the therapeutic potential of ncRNA-mediated epigenetic regulation is promising, challenges such as delivery efficiency, off-target effects, and understanding the complex regulatory networks remain. The intersection of ncRNAs and epigenetic regulation offers a rich landscape for therapeutic innovation. Targeting specific ncRNAs or modulating epigenetic modifiers holds great potential for developing precise, personalized therapies across various diseases. Continued research and clinical validation will be essential in realizing the full therapeutic impact of ncRNA-mediated epigenetic regulation. Synthetic miRNA mimics are designed to restore the function of downregulated miRNAs. These mimics can inhibit target genes, potentially reverting aberrant cellular processes. For example, miR-34a mimics are being explored for their tumor-suppressive effects in various cancers. AMOs are short synthetic RNA sequences designed to specifically inhibit miRNAs. These AMOs can effectively silence overexpressed miRNAs, thereby halting their oncogenic or disease-promoting effects. Utilizing siRNAs or ASOs to specifically target and degrade disease-associated lncRNAs shows promise in mitigating their pathological effects. AAVs have gained attention as efficient vehicles for ncRNA delivery. Engineered AAVs can carry miRNAs or small hairpin RNAs (shRNAs) to target specific cellular processes, showing potential in various genetic disorders. Exosomes, naturally occurring vesicles, can be engineered to carry ncRNAs. These exosomes can serve as vehicles for targeted delivery, potentially enhancing therapeutic efficacy and reducing off-target effects (Ou et al., 2019).

While ncRNA-based therapies hold great promise, challenges such as efficient delivery, off-target effects, and understanding complex regulatory networks remain. Future research efforts should focus on refining delivery methods, elucidating ncRNA functions, and conducting rigorous clinical trials to validate the efficacy and safety of these therapeutic approaches. The ncRNA-based therapies represent a revolutionary approach in precision medicine. Targeting specific ncRNAs holds great potential for developing precise, personalized therapies across a spectrum of diseases. Continued research and clinical validation will be essential in realizing the full therapeutic impact of ncRNA-based interventions (Winkle et al., 2021).

### Combination therapies and similar approaches

The integration of various therapeutic modalities represents a powerful strategy in addressing complex diseases. This note explores the concept of combined approaches, where ncRNA-based therapies are synergistically employed with other treatment modalities, offering a multifaceted approach to disease management. Combining miRNA mimics or inhibitors with mRNA-targeted therapies can yield synergistic effects. This approach enables the simultaneous modulation of upstream regulatory miRNAs and their downstream targets, amplifying therapeutic outcomes. Integrating ncRNA-based strategies with conventional treatments, such as chemotherapy or radiation therapy, can enhance treatment responses. For instance, miRNA-based therapies can sensitize cancer cells to chemotherapy, potentially overcoming drug resistance. Combining epigenetic modifiers, like DNMT or HDAC inhibitors, with ncRNA-based therapies can achieve comprehensive epigenetic reprogramming. This dual strategy targets both global and specific epigenetic alterations, offering a powerful approach in disease management. Tailoring combined therapies based on patient-specific ncRNA expression profiles allows for a highly personalized approach. By understanding the unique regulatory networks at play, treatments can be optimized to address the specific molecular landscape of each patient’s disease. Combining ncRNA-based therapies that target different pathways with complementary conventional treatments can circumvent resistance mechanisms. While combined approaches show great promise, challenges such as dosing optimization, potential off-target effects, and individual patient response variations need to be carefully addressed. This multi-pronged approach disrupts multiple points of cellular regulation, potentially achieving more durable responses (Winkle et al., 2021). Finally, we believe that, experimental research, preclinical studies and clinical trials (in different phases) are essential for validating the full therapeutic potential of ncRNA interventions with conventional therapies or epigenetic modifiers, towards revolutionizing the landscape of precision medicine (Table 2).

**TABLE 2:** Clinical trials and translational development of ncRNA-based therapeutics targeting cancer stemness, EMT, and therapy resistance.

Therapeutic agent	ncRNA target/Type	Cancer type	Mechanism related to Stemness/EMT/Drug resistance	Clinical status	Major findings	References
MRX34	miR-34a mimic	Advanced solid tumors, hepatocellular carcinoma, melanoma and others	Restores tumor-suppressive miR-34a; suppresses CSC-associated genes including NOTCH1, BCL2, CD44, MET and stemness signaling pathways	Phase I (terminated)	First miRNA mimic tested in cancer patients; demonstrated biological activity but trial was terminated due to immune-related adverse events	Beg et al. (2017), Hong et al. (2020)
Cobomarsen (MRG-106)	Anti-miR-155 oligonucleotide	Cutaneous T-cell lymphoma (CTCL) and hematological malignancies	Inhibits oncogenic miR-155 involved in proliferation, stem-like phenotypes, inflammation and treatment resistance	Phase I/II	Demonstrated acceptable safety profile and preliminary clinical responses	Querfeld et al. (2018)
TargomiRs	miR-16 mimic delivered by bacterial minicells	Malignant pleural mesothelioma and NSCLC	Restores tumor-suppressive miR-16, suppressing cell-cycle progression and survival pathways associated with CSC maintenance	Phase I completed	Demonstrated feasibility of targeted miRNA delivery and evidence of antitumor activity	Viteri and Rosell (2018)
MesomiR-1 (TargomiR platform)	miR-16 mimic	Recurrent malignant pleural mesothelioma	Downregulates genes involved in survival, proliferation and therapy resistance	Phase I completed	Established proof-of-concept for systemic miRNA replacement therapy	van Zandwijk et al. (2015)
STP705	siRNA targeting TGF-β1 and COX-2	Advanced solid tumors and skin cancers	Inhibits TGF-β-mediated EMT, fibrosis, invasion and CSC-associated signaling	Phase I/II ongoing	Demonstrated favorable safety profile and preliminary antitumor efficacy	Nestor et al. (2024)
Atu027	siRNA targeting PKN3	Advanced solid tumors	Targets PI3K/PKN3 signaling involved in migration, metastasis, EMT and vascular remodeling	Phase I completed	Well tolerated with evidence of disease stabilization in some patients	Schultheis et al. (2020)
EPHARNA (DOPC-EphA2-siRNA)	EphA2-targeted siRNA	Recurrent ovarian and other solid tumors	Suppresses EphA2 signaling associated with stemness, EMT and metastatic progression	Phase I completed	Demonstrated successful nanoparticle-mediated siRNA delivery	Reddy et al. (2025)
Custirsen (OGX-011)	Antisense oligonucleotide against Clusterin	Metastatic castration-resistant prostate cancer	Targets therapy resistance mechanisms and survival pathways associated with treatment-refractory CSC populations	Phase II/III completed	Improved biomarker responses but failed to significantly improve overall survival in phase III studies	Beer et al. (2017)
Danvatirsen (AZD9150)	Antisense oligonucleotide against STAT3	Lymphoma and advanced solid tumors	Inhibits STAT3 signaling, a key regulator of CSC maintenance, EMT and immune evasion	Phase I/II completed and ongoing combinations	Demonstrated pathway inhibition and encouraging activity in combination regimens	Proia et al. (2020)
BP1001	Liposomal Grb2 antisense oligonucleotide	Leukemia and solid tumors	Targets GRB2-mediated signaling involved in stemness, proliferation and resistance	Early-phase clinical trials	Demonstrated safety and potential therapeutic activity in hematologic malignancies	Ohanian et al. (2018)

CSC, cancer stem cell; EMT, epithelial–mesenchymal transition; CTCL, cutaneous T-cell lymphoma; NSCLC, non-small cell lung cancer; siRNA, small interfering RNA; TGF-β, transforming growth factor-beta; STAT3, signal transducer and activator of transcription 3.

## Clinical translation challenges and emerging therapeutic strategies for ncRNA-based cancer therapy

There has been remarkable progress in understanding the roles of ncRNAs in regulating cancer stemness, EMT and therapeutic resistance, but the translation of these findings into clinically effective therapeutics remains challenging (John et al., 2025). Many preclinical studies have shown the efficacy of miRNA mimics, antisense oligonucleotides, siRNAs, lncRNA inhibitors and circRNA-targeting strategies; however, only a few ncRNA-based therapeutics have reached clinical testing (Yan et al., 2025a). Significant hurdles include low *in vivo* stability, fast degradation by nucleases, poor intracellular delivery, lack of tumor specificity, immune stimulation, off-target gene regulation and manufacturing issues. Moreover, the broad crosstalk of ncRNAs with various signaling pathways makes the prediction of therapeutic responses and possible side effects challenging (Sultana et al., 2026). Thus, overcoming these barriers in translation is critical for successful clinical application of precision oncology guided by ncRNAs.

### Barriers to delivery and tumor-specific targeting

Efficient delivery remains one of the major challenges for ncRNA therapeutics. Endogenous nucleases rapidly degrade naked RNA molecules in circulation and the large size, negative charge and susceptibility to renal clearance of naked RNA molecules results in poor cellular uptake (Song, 2026). In addition, physiological barriers such as vascular endothelium, extracellular matrix, stromal fibrosis, hypoxic regions, and high interstitial pressure further limit penetration into solid tumors. Another major limitation is that therapeutic RNAs once taken up must escape the endosomal degradation pathway before reaching their intracellular targets (Sorokin, 2026). Delivery issues are especially prominent in tumors with an abundance of CSCs where protective niches within the tumor microenvironment limit access to therapies. Hence, the development of targeted delivery systems that are able to selectively deliver ncRNAs to CSCs while minimizing systemic exposure has become a major research priority (Imtiaz et al., 2025).

### Delivery systems based on nanocarriers

Nanotechnology has emerged as one of the most promising strategies to overcome delivery limitations associated to ncRNA therapeutics. Lipid nanoparticles (LNPs), polymeric nanoparticles, lipid-polymer hybrid nanoparticles, dendrimers, micelles and inorganic nanomaterials can protect therapeutic RNAs from nuclease degradation, and can increase circulation time, intracellular uptake and controlled release (Pandey et al., 2026). Surface modification of NPs with tumor targeting ligands, antibodies, aptamers or peptides also further enhances selective accumulation in CSC-enriched tumors via passive and active targeting. Recent studies have demonstrated that nanoparticle-mediated delivery of miRNA mimics, siRNAs, antisense oligonucleotides and CRISPR/Cas components effectively inhibits stemness-associated signaling pathways, including Wnt/β-catenin, Notch, Hedgehog, PI3K/AKT and STAT3 signaling (Gomerdinger et al., 2025). In addition, active development of stimulus-responsive nanoparticles that release therapeutic cargo in response to acidic pH, hypoxia, redox potential or enzymatic activity within the tumor microenvironment is ongoing to improve therapeutic precision while reducing systemic toxicity (Ahmad et al., 2026).

### Exosome-based delivery of ncRNAs

Natural nanocarriers such as exosomes have attracted great attention due to their inherent biocompatibility, low immunogenicity and ability to efficiently transport biological cargo across physiological barriers. These extracellular vesicles naturally encapsulate miRNAs, lncRNAs, circRNAs, proteins, and lipids, protecting them from enzymatic degradation during their systemic circulation (Zhang T. et al., 2025). Engineered exosomes from mesenchymal stem cells, dendritic cells or immune cells have been studied as vehicles for delivery of tumor-suppressive miRNAs, siRNAs, CRISPR/Cas constructs and antisense oligonucleotides directly to cancer cells and CSC populations (Qazi et al., 2026). The ability of exosomes to cross biological barriers, including the blood-brain barrier, makes them particularly attractive for the treatment of metastatic and brain malignancies. However, various challenges such as large-scale production, purification, cargo loading efficiency, storage stability, biodistribution and quality control need to be overcome before large-scale clinical application can be realized (Kim et al., 2024).

### Viral vector platforms: AAV-based delivery

Viral vectors are still very efficient platforms for the stable delivery of genes. Among these, adeno-associated virus (AAV) vectors have attracted much attention due to their relatively low immunogenicity, good safety profile and ability to achieve long-term expression of therapeutic RNAs (Singh K. et al., 2025). AAV-mediated delivery has been investigated for expression of tumor-suppressive miRNAs, inhibitory RNAs, CRISPR/Cas genome editing components, and RNA-guided transcriptional regulators capable of silencing oncogenic ncRNAs (Gil et al., 2026). These vectors provide prolonged therapeutic expression with reduced need for repeated administration. Important limitations remain, however, including limited packaging capacity, pre-existing neutralizing antibodies, vector immunogenicity, dose-dependent hepatotoxicity, and challenges with repeat dosing (Pan et al., 2025). Future clinical application could benefit from improved targeting specificity and safety through further capsid engineering, tissue-specific promoters and next-generation AAV serotypes.

### Safety considerations and side effects

Since individual ncRNAs often regulate hundreds of downstream genes and are part of large regulatory networks, off-target effects on non-target genes remain a major safety concern. Off-target interactions can alter normal cell function, disrupt immune homeostasis, or cause unexpected toxicities in healthy tissues (Chiu et al., 2025). Furthermore, some RNA formulations and delivery platforms have been shown to activate innate immune receptors, induce cytokine release, activate complement and cause hepatotoxicity and renal toxicity. Long-term inhibition of broadly acting oncogenic ncRNAs may also interfere with normal tissue regeneration and stem cell function (Nguyen et al., 2025). To minimize such risks, current optimization strategies include chemical modification of oligonucleotides, sequence optimization, tissue-specific promoters, ligand-directed delivery systems, inducible expression constructs, and advanced computational algorithms to improve target prediction and reduce off-target interactions (Wang et al., 2025e).

### Strategies of combination therapy

There is growing evidence that ncRNA therapeutics are likely to be most effective in clinical use when combined with conventional anticancer treatments rather than as monotherapies (Yan et al., 2025). Restoration of tumor-suppressive miRNAs or inhibition of oncogenic ncRNAs can sensitize CSCs to chemotherapy, radiotherapy, targeted therapy and immune checkpoint inhibitors, through simultaneous suppression of stemness-associated signaling pathways and reversal of EMT (Yan et al., 2025). Combination strategies of ncRNA therapeutics with DNA methyltransferase inhibitors, histone deacetylase inhibitors, bromodomain inhibitors and other epigenetic drugs have demonstrated synergistic antitumor activity by reprogramming aberrant epigenetic landscapes and restoring normal gene expression profiles. Similarly, co-delivery of ncRNA therapeutics and chemotherapeutic agents via multifunctional nanocarriers has resulted in increased intracellular drug accumulation, decreased multidrug resistance, and increased apoptosis in preclinical models (Jiang et al., 2026). Such integrated therapeutic approaches may provide more durable responses by simultaneously targeting bulk tumor cells, cancer stem cells and the supportive tumor microenvironment.

## Conclusion

NcRNAs have emerged as master regulators of cancer stemness, EMT, and therapy resistance, orchestrating complex molecular networks that drive tumor progression, metastasis, recurrence, and treatment failure. Through their ability to regulate stemness-associated transcription factors, EMT regulators, epigenetic machinery, and key signaling pathways including Wnt/β-catenin, Notch, Hedgehog, PI3K/AKT/mTOR, JAK/STAT, NF-κB, and Hippo/YAP, ncRNAs play a central role in maintaining CSC plasticity and survival under therapeutic stress (Kadian et al., 2024). Accumulating evidence indicates that dysregulated miRNAs, lncRNAs, and circRNAs not only contribute to the acquisition of stem-like traits and drug resistance but also serve as promising biomarkers for cancer diagnosis, prognosis, treatment response, and disease monitoring (Wang et al., 2025d).

The growing understanding of ncRNA-mediated regulatory networks has opened new opportunities for therapeutic intervention. Strategies based on antisense oligonucleotides, siRNAs, miRNA mimics, CRISPR-mediated genome editing, and nanoparticle-assisted delivery systems are showing encouraging results in preclinical and early clinical studies. However, challenges related to tumor heterogeneity, delivery efficiency, off-target effects, and the dynamic nature of ncRNA signaling networks remain major obstacles to clinical translation.

The future of ncRNA-based precision oncology lies in integrating multi-omics profiling, artificial intelligence-assisted biomarker discovery, and personalized delivery platforms to identify patient-specific therapeutic targets (Liu et al., 2026). Advances in RNA engineering, programmable nanoparticles, engineered exosomes, CRISPR-based RNA editing, and synthetic biology are expected to substantially improve delivery efficiency, specificity, and long-term safety (Luo et al., 2025). Moreover, combining ncRNA therapeutics with immunotherapy, epigenetic modulators, targeted therapies, and conventional chemotherapy offers a promising strategy for simultaneously eliminating cancer stem cells and overcoming adaptive drug resistance. Continued multidisciplinary collaboration, rigorous preclinical validation, and well-designed clinical trials will be essential to translate these innovative approaches into effective therapies for patients with refractory and metastatic cancers (Song et al., 2025). Such efforts will facilitate the identification of robust biomarkers and novel therapeutic targets, ultimately enabling the development of more precise and durable treatment strategies aimed at eliminating CSC populations, preventing tumor relapse, and improving patient outcomes across diverse cancer types (Wang Y. et al., 2026).

## References

[B1] Ahmad Q. Mehdi S. Shaukat B. Siddique R. Asif M. T. Malik A. (2026). Multi-stimuli responsive nanoparticles: next-generation platforms for smart drug delivery. OpenNano 29, 100296. 10.1016/j.onano.2026.100296

[B2] Al-khreisat M. J. Abdulsahib W. K. Jasim I. K. Malathi H. Nayak P. P. Anand D. A. (2026). Targeting cancer stem cell plasticity and tumor microenvironment crosstalk: a comprehensive review. Discov. Onc. 17, 145. 10.1007/s12672-025-04297-y PMC12834896 41422192

[B3] Ali Hosseini Rad S. M. Bavarsad M. S. Arefian E. Jaseb K. Shahjahani M. Saki N. (2013). The role of microRNAs in stemness of cancer stem cells. Oncol. Rev. 7, e8. 10.4081/oncol.2013.e8 25992229 PMC4419617

[B4] Alkan A. H. Cansaran-Duman D. (2025). Multifaceted interactions between lncRNA-associated ceRNA networks and small molecules in triple-negative breast cancer. Biomed. Pharmacother. 189, 118241. 10.1016/j.biopha.2025.118241 40527037

[B5] Allgayer H. Mahapatra S. Mishra B. Swain B. Saha S. Khanra S. (2025). Epithelial-to-mesenchymal transition (EMT) and cancer metastasis: the status quo of methods and experimental models 2025. Mol. Cancer 24, 167. 10.1186/s12943-025-02338-2 40483504 PMC12144846

[B6] Alshahrani M. Y. Saleh R. O. Hjazi A. Bansal P. Kaur H. Deorari M. (2024). Molecular mechanisms of tumorgenesis and metastasis of long non-coding RNA (lncRNA) NEAT1 in human solid tumors; an update. Cell Biochem. Biophys. 82, 593–607. 10.1007/s12013-024-01287-9 38750383

[B7] Alshamrani A. A. (2020). Roles of microRNAs in ovarian cancer tumorigenesis: two decades later, what have we learned? Front. Oncol. 10, 1084. 10.3389/fonc.2020.01084 32850313 PMC7396563

[B8] Anil P. Ghosh Dastidar S. Banerjee S. (2022). Unravelling the role of long non-coding RNAs in prostate carcinoma. Adv. Cancer Biol. Metastasis 6, 100067. 10.1016/j.adcanc.2022.100067

[B9] Aria H. Azizi M. Nazem S. Mansoori B. Darbeheshti F. Niazmand A. (2024). Competing endogenous RNAs regulatory crosstalk networks: the messages from the RNA world to signaling pathways directing cancer stem cell development. Heliyon 10, e35208. 10.1016/j.heliyon.2024.e35208 39170516 PMC11337742

[B10] Beer T. M. Hotte S. J. Saad F. Alekseev B. Matveev V. Fléchon A. (2017). Custirsen (OGX-011) combined with cabazitaxel and prednisone versus cabazitaxel and prednisone alone in patients with metastatic castration-resistant prostate cancer previously treated with docetaxel (AFFINITY): a randomised, open-label, international, phase 3 trial. Lancet Oncol. 18, 1532–1542. 10.1016/S1470-2045(17)30605-8 29033099

[B11] Beg M. S. Brenner A. J. Sachdev J. Borad M. Kang Y.-K. Stoudemire J. (2017). Phase I study of MRX34, a liposomal miR-34a mimic, administered twice weekly in patients with advanced solid tumors. Invest New Drugs 35, 180–188. 10.1007/s10637-016-0407-y 27917453 PMC5893501

[B12] Bhan A. Mandal S. S. (2015). LncRNA HOTAIR: a master regulator of chromatin dynamics and cancer. Biochim. Biophys. Acta 1856, 151–164. 10.1016/j.bbcan.2015.07.001 26208723 PMC4544839

[B13] Bhaskaran M. Mohan M. (2014). MicroRNAs: history, biogenesis, and their evolving role in animal development and disease. Vet. Pathol. 51, 759–774. 10.1177/0300985813502820 24045890 PMC4013251

[B14] Bhat G. R. Sethi I. Sadida H. Q. Rah B. Mir R. Algehainy N. (2024). Cancer cell plasticity: from cellular, molecular, and genetic mechanisms to tumor heterogeneity and drug resistance. Cancer Metastasis Rev. 43, 197–228. 10.1007/s10555-024-10172-z 38329598 PMC11016008

[B15] Bhushan R. Rai S. Jangra P. Sonker P. Singh N. Lakshami R. (2025). The role of artificial intelligence in predicting cancer immunotherapy response. Oral Oncol. Rep. 16, 100767. 10.1016/j.oor.2025.100767

[B16] Bhushan R. Sonker P. Singh P. Singh N. Rai S. Philip R. R. (2026). Nanobots in medicine and beyond: from targeted drug delivery to intelligent environmental remediation. Next Mater. 11, 101791. 10.1016/j.nxmate.2026.101791

[B17] Bitaraf A. Zafarani A. Jahandideh P. Hakak-Zargar B. Haghi A. Asgaritarghi G. (2025). MALAT1 as a molecular driver of tumor progression, immune evasion, and resistance to therapy. Mol. Cancer 24, 245. 10.1186/s12943-025-02415-6 41053822 PMC12502584

[B18] Bure I. V. Nemtsova M. V. (2023). Mutual regulation of ncRNAs and chromatin remodeling complexes in normal and pathological conditions. Int. J. Mol. Sci. 24, 7848. 10.3390/ijms24097848 37175555 PMC10178202

[B19] Burgos M. Hurtado A. Jiménez R. Barrionuevo F. J. (2021). Non-coding RNAs: lncRNAs, miRNAs, and piRNAs in sexual development. Sex. Dev. 15, 335–350. 10.1159/000519237 34614501

[B20] Cai Y. Huang S. Dong Y. Li S. Jin X. (2026). PIWI-interacting RNAs in brain health and disease: biogenesis, mechanisms, and therapeutic horizons. Psychopharmacology 243, 1377–1396. 10.1007/s00213-025-06958-w 41182353

[B21] Calin G. A. Ferracin M. Cimmino A. Di Leva G. Shimizu M. Wojcik S. E. (2005). A MicroRNA signature associated with prognosis and progression in chronic lymphocytic leukemia. N. Engl. J. Med. 353, 1793–1801. 10.1056/NEJMoa050995 16251535

[B22] Cao L. Xie B. Yang X. Liang H. Jiang X. Zhang D. (2015). MiR-324-5p suppresses hepatocellular carcinoma cell invasion by counteracting ECM degradation through post-transcriptionally downregulating ETS1 and SP1. PLoS One 10, e0133074. 10.1371/journal.pone.0133074 26177288 PMC4503725

[B23] Carlberg C. (2024). “Histone modifications,” in Gene Regulation and Epigenetics: How Science Works. Editor Carlberg C. (Cham: Springer Nature Switzerland), 103–117. 10.1007/978-3-031-68730-3_8

[B24] Carrà G. Avalle L. Seclì L. Brancaccio M. Morotti A. (2022). Shedding light on NF-κB functions in cellular organelles. Front. Cell Dev. Biol. 10, 841646. 10.3389/fcell.2022.841646 35620053 PMC9127296

[B25] Castro R. E. Ferreira D. M. S. Afonso M. B. Borralho P. M. Machado M. V. Cortez-Pinto H. (2013). miR-34a/SIRT1/p53 is suppressed by ursodeoxycholic acid in the rat liver and activated by disease severity in human non-alcoholic fatty liver disease. J. Hepatol. 58, 119–125. 10.1016/j.jhep.2012.08.008 22902550

[B26] Castro-Oropeza R. Melendez-Zajgla J. Maldonado V. Vazquez-Santillan K. (2018). The emerging role of lncRNAs in the regulation of cancer stem cells. Cell Oncol. 41, 585–603. 10.1007/s13402-018-0406-4 30218296 PMC12995221

[B27] Chen L.-L. Kim V. N. (2024). Small and long non-coding RNAs: past, present, and future. Cell 187, 6451–6485. 10.1016/j.cell.2024.10.024 39547208

[B28] Chen F. Fu Y. Bai G. Qiu J. Hua K. (2025). Multi-omics dissection of tumor microenvironment-mediated drug resistance: mechanisms and therapeutic reprogramming. Front. Pharmacol. 16, 1634413. 10.3389/fphar.2025.1634413 40693266 PMC12277294

[B29] Chiu H.-S. Somvanshi S. Chang C.-T. de Bony de Lavergne E. J. Wei Z. Hsieh C.-H. (2025). Coordinated regulation by lncRNAs results in tight lncRNA-target couplings. Cell Genomics 5, 100927. 10.1016/j.xgen.2025.100927 40628267 PMC12367320

[B30] Chodurska B. Kunej T. (2025). Long non-coding RNAs in humans: classification, genomic organization and function. Non-coding RNA Res. 11, 313–327. 10.1016/j.ncrna.2025.01.004 PMC11833636 39967600

[B31] Chu X. Tian W. Ning J. Xiao G. Zhou Y. Wang Z. (2024). Cancer stem cells: advances in knowledge and implications for cancer therapy. Sig Transduct. Target Ther. 9, 170. 10.1038/s41392-024-01851-y PMC11224386 38965243

[B32] Cong G. Zhu X. Chen X. R. Chen H. Chong W. (2025). Mechanisms and therapeutic potential of the hedgehog signaling pathway in cancer. Cell Death Discov. 11, 40. 10.1038/s41420-025-02327-w 39900571 PMC11791101

[B33] da Silva L. F. C. M. Carneiro F. P. Motoyama A. B. (2026). MicroRNA expression in breast cancer patients, an integrative review. J. Surg. Oncol. 133, 31–38. 10.1002/jso.70132 41241871 PMC12747699

[B34] Dai X. Fang M. Li S. Yan Y. Zhong Y. Du B. (2017). miR-21 is involved in transforming growth factor β1-induced chemoresistance and invasion by targeting PTEN in breast cancer. Oncol. Lett. 14, 6929–6936. 10.3892/ol.2017.7007 29151919 PMC5678423

[B35] Dai J. Su Y. Zhong S. Cong L. Liu B. Yang J. (2020). Exosomes: key players in cancer and potential therapeutic strategy. Sig Transduct. Target Ther. 5, 145. 10.1038/s41392-020-00261-0 32759948 PMC7406508

[B36] Dakal T. C. Bhushan R. Xu C. Gadi B. R. Cameotra S. S. Yadav V. (2024). Intricate relationship between cancer stemness, metastasis, and drug resistance. MedComm 5, e710. 10.1002/mco2.710 39309691 PMC11416093

[B37] Deng Z. Fan T. Xiao C. Tian H. Zheng Y. Li C. (2024). TGF-β signaling in health, disease and therapeutics. Sig Transduct. Target Ther. 9, 61. 10.1038/s41392-024-01764-w PMC10958066 38514615

[B38] Desai S. A. Patel V. P. Bhosle K. P. Nagare S. D. Thombare K. C. (2025). The tumor microenvironment: shaping cancer progression and treatment response. J. Chemother. 37, 15–44. 10.1080/1120009X.2023.2300224 38179655

[B39] Dhar G. A. Saha S. Mitra P. Nag Chaudhuri R. (2021). DNA methylation and regulation of gene expression: guardian of our health. Nucleus 64, 259–270. 10.1007/s13237-021-00367-y PMC8366481 34421129

[B40] Du M. Zhang J. Wicha M. S. Luo M. (2024). Redox regulation of cancer stem cells: biology and therapeutic implications. MedComm Oncol. 3, e70005. 10.1002/mog2.70005

[B41] Duan Z. Zhou Y. Wu Y. (2025). Mapping thirty years of tumour-microenvironment-driven drug resistance in breast cancer: a global bibliometric analysis. Discov. Oncol. 16, 1489. 10.1007/s12672-025-03324-2 40772981 PMC12332156

[B42] El-Ashmawy N. E. Khedr E. G. Darwish R. T. Ibrahim A. O. (2025). Competing endogenous RNAs network and therapeutic implications: new horizons in disease research. Biochimica Biophysica Acta Gene Regul. Mech. 1868, 195073. 10.1016/j.bbagrm.2024.195073 39631541

[B43] Eldakhakhny B. Sutaih A. M. Siddiqui M. A. Aqeeli Y. M. Awan A. Z. Alsayegh M. Y. (2024). Exploring the role of noncoding RNAs in cancer diagnosis, prognosis, and precision medicine. Noncoding RNA Res. 9, 1315–1323. 10.1016/j.ncrna.2024.06.015 39764154 PMC11703603

[B44] Elimam H. Radwan A. F. El Said N. H. Elfar N. Abd-Elmawla M. A. Aborehab N. M. (2025). Long non-coding RNAs and signaling networks in non-small cell lung cancer: mechanistic insights into tumor pathogenesis. Cancer Gene Ther. 32, 1145–1165. 10.1038/s41417-025-00950-4 40954328

[B45] Endale H. T. Mariye Y. F. Negash H. K. Hassen F. S. Asrat W. B. Mengstie T. A. (2024). MiRNA in cervical cancer: diagnosis to therapy: systematic review. Heliyon 10, e24398. 10.1016/j.heliyon.2024.e24398 38317930 PMC10839805

[B46] Farasati Far B. Vakili K. Fathi M. Yaghoobpoor S. Bhia M. Naimi- Jamal M. R. (2023). The role of microRNA-21 (miR-21) in pathogenesis, diagnosis, and prognosis of gastrointestinal cancers: a review. Life Sci. 316, 121340. 10.1016/j.lfs.2022.121340 36586571

[B47] Fischer J. W. Leung A. K. L. (2017). CircRNAs: a regulator of cellular stress. Crit. Rev. Biochem. Mol. Biol. 52, 220–233. 10.1080/10409238.2016.1276882 28095716 PMC5526226

[B48] Galassi C. Manic G. Esteller M. Galluzzi L. Vitale I. (2025). Epigenetic regulation of cancer stemness. Sig Transduct. Target Ther. 10, 243. 10.1038/s41392-025-02340-6 PMC12314033 40744921

[B49] Gao N. Li Y. Li J. Gao Z. Yang Z. Li Y. (2020). Long non-coding RNAs: the regulatory mechanisms, research strategies, and future directions in cancers. Front. Oncol. 10, 598817. 10.3389/fonc.2020.598817 33392092 PMC7775490

[B50] Ghafouri-Fard S. Taheri M. (2019). *UCA1* long non-coding RNA: an update on its roles in malignant behavior of cancers. Biomed. Pharmacother. 120, 109459. 10.1016/j.biopha.2019.109459 31585301

[B51] Ghafouri-Fard S. Shirvani-Farsani Z. Branicki W. Taheri M. (2020). MicroRNA signature in renal cell carcinoma. Front. Oncol. 10, 596359. 10.3389/fonc.2020.596359 33330087 PMC7734191

[B52] Ghafouri-Fard S. Glassy M. C. Abak A. Hussen B. M. Niazi V. Taheri M. (2021). The interaction between miRNAs/lncRNAs and Notch pathway in human disorders. Biomed. Pharmacother. 138, 111496. 10.1016/j.biopha.2021.111496 33743335

[B53] Ghorbian S. (2025). Cancer cell plasticity and therapeutic resistance: mechanisms, crosstalk, and translational perspectives. Hereditas 162, 188. 10.1186/s41065-025-00564-8 41013839 PMC12465352

[B54] Gil J.-S. Lee S. Koo T. (2026). Therapeutic in vivo genome editing: innovations and challenges in rAAV vector-based CRISPR delivery. Gene Ther. 33, 97–106. 10.1038/s41434-025-00573-2 41224955 PMC12932096

[B55] Godlewski J. Nowicki M. O. Bronisz A. Nuovo G. Palatini J. De Lay M. (2010). MicroRNA-451 regulates LKB1/AMPK signaling and allows adaptation to metabolic stress in glioma cells. Mol. Cell 37, 620–632. 10.1016/j.molcel.2010.02.018 20227367 PMC3125113

[B56] Gomerdinger V. F. Nabar N. Hammond P. T. (2025). Advancing engineering design strategies for targeted cancer nanomedicine. Nat. Rev. Cancer 25, 657–683. 10.1038/s41568-025-00847-2 40751005

[B57] Gou L.-T. Zhu Q. Liu M.-F. (2023). Small RNAs: an expanding world with therapeutic promises. Fundam. Res. 3, 676–682. 10.1016/j.fmre.2023.03.003 38933305 PMC11197668

[B58] Guessous F. Alvarado-Velez M. Marcinkiewicz L. Zhang Y. Kim J. Heister S. (2013). Oncogenic effects of miR-10b in glioblastoma stem cells. J. Neurooncol 112, 153–163. 10.1007/s11060-013-1047-0 23307328 PMC3609924

[B59] Guo T. Xu J. (2024). Cancer-associated fibroblasts: a versatile mediator in tumor progression, metastasis, and targeted therapy. Cancer Metastasis Rev. 43, 1095–1116. 10.1007/s10555-024-10186-7 38602594 PMC11300527

[B60] Guo Q. Jin Y. Chen X. Ye X. Shen X. Lin M. (2024). NF-κB in biology and targeted therapy: new insights and translational implications. Sig Transduct. Target Ther. 9, 53. 10.1038/s41392-024-01757-9 PMC10910037 38433280

[B61] Guo L. Lin J. Ren Q. Sun H. Wu Y. Ge H. (2025). Enhanced activities of OCT4 and SOX2 promote epigenetic reprogramming by shortening G1 phase. Adv. Sci. 12, e15528. 10.1002/advs.202415528 PMC12407333 40448624

[B62] Gupta R. A. Shah N. Wang K. C. Kim J. Horlings H. M. Wong D. J. (2010). Long non-coding RNA HOTAIR reprograms chromatin state to promote cancer metastasis. Nature 464, 1071–1076. 10.1038/nature08975 20393566 PMC3049919

[B63] Haddadin L. Sun X. (2025). Stem cells in cancer: from mechanisms to therapeutic strategies. Cells 14, 538. 10.3390/cells14070538 40214491 PMC11988674

[B64] Hadjimichael C. Chanoumidou K. Papadopoulou N. Arampatzi P. Papamatheakis J. Kretsovali A. (2015). Common stemness regulators of embryonic and cancer stem cells. World J. Stem Cells 7, 1150–1184. 10.4252/wjsc.v7.i9.1150 26516408 PMC4620423

[B65] Hasani F. Masrour M. Khamaki S. Jazi K. Hosseini S. Heidarpour H. (2025). Diagnostic and prognostic accuracy of MiRNAs in pancreatic cancer: a systematic review and meta-analysis. J. Cell. Mol. Med. 29, e70337. 10.1111/jcmm.70337 39855897 PMC11761000

[B66] Hong D. S. Kang Y.-K. Borad M. Sachdev J. Ejadi S. Lim H. Y. (2020). Phase 1 study of MRX34, a liposomal miR-34a mimic, in patients with advanced solid tumours. Br. J. Cancer 122, 1630–1637. 10.1038/s41416-020-0802-1 32238921 PMC7251107

[B67] Hossam Abdelmonem B. Kamal L. T. Wardy L. W. Ragheb M. Hanna M. M. M. Elsharkawy M. (2025). Non-coding RNAs: emerging biomarkers and therapeutic targets in cancer and inflammatory diseases. Front. Oncol. 15, 1534862. 10.3389/fonc.2025.1534862 40129920 PMC11931079

[B68] Howe E. N. Cochrane D. R. Richer J. K. (2011). Targets of miR-200c mediate suppression of cell motility and anoikis resistance. Breast Cancer Res. 13, R45. 10.1186/bcr2867 21501518 PMC3219208

[B69] Hua J. Wang Z. Cheng X. Dai J. Zhao P. (2025). Circular RNAs modulate cancer drug resistance: advances and challenges. Cancer Drug Resist 8, 17. 10.20517/cdr.2024.195 40201313 PMC11977347

[B70] Huang A. Zheng H. Wu Z. Chen M. Huang Y. (2020). Circular RNA-protein interactions: functions, mechanisms, and identification. Theranostics 10, 3503–3517. 10.7150/thno.42174 32206104 PMC7069073

[B71] Ibekwe P.-M. R. Akintayo E. A. Okuku C. N. Muhammed I. Jeje F. M. Okun O. (2025). Decoding tumor heterogeneity through multi omics: insights into cancer evolution, microenvironment and therapy resistance. J. Cancer Tumor Int. 15, 91–112. 10.9734/jcti/2025/v15i3305

[B72] Imtiaz S. Ferdous U. T. Nizela A. Hasan A. Shakoor A. Zia A. W. (2025). Mechanistic study of cancer drug delivery: current techniques, limitations, and future prospects. Eur. J. Med. Chem. 290, 117535. 10.1016/j.ejmech.2025.117535 40132495

[B73] Iorio M. V. Ferracin M. Liu C.-G. Veronese A. Spizzo R. Sabbioni S. (2005). MicroRNA gene expression deregulation in human breast cancer. Cancer Res. 65, 7065–7070. 10.1158/0008-5472.CAN-05-1783 16103053

[B74] Ji Q. Hao X. Zhang M. Tang W. Yang M. Li L. (2009). MicroRNA miR-34 inhibits human pancreatic cancer tumor-initiating cells. PLoS One 4, e6816. 10.1371/journal.pone.0006816 19714243 PMC2729376

[B75] Ji Q. Zhang L. Liu X. Zhou L. Wang W. Han Z. (2014). Long non-coding RNA MALAT1 promotes tumour growth and metastasis in colorectal cancer through binding to SFPQ and releasing oncogene PTBP2 from SFPQ/PTBP2 complex. Br. J. Cancer 111, 736–748. 10.1038/bjc.2014.383 25025966 PMC4134507

[B76] Jiang M. Zhang K. Zhang Z. Zeng X. Huang Z. Qin P. (2025). PI3K/AKT/mTOR axis in cancer: from pathogenesis to treatment. MedComm 6, e70295. 10.1002/mco2.70295 40740483 PMC12308072

[B77] Jiang L. Yuan Y. Zhang D. Wang J. Jiang X. Shi Y. (2026). Epigenetic regulation in cancer chemoresistance and combined therapeutic strategies. Front. Cell Dev. Biol. 14, 1814084. 10.3389/fcell.2026.1814084 42052571 PMC13111467

[B78] Jimenez-Castaño R. Nieto M. A. (2026). Epithelial-to-mesenchymal transition as a central driver of tumor cell plasticity. Nat. Cancer 7, 567–582. 10.1038/s43018-026-01154-x 42032343

[B79] Jing J. Wu Z. Wang J. Luo G. Lin H. Fan Y. (2023). Hedgehog signaling in tissue homeostasis, cancers and targeted therapies. Sig Transduct. Target Ther. 8, 315. 10.1038/s41392-023-01559-5 37596267 PMC10439210

[B80] John A. Almulla N. Elboughdiri N. Gacem A. Yadav K. K. Abass A. M. (2025). Non-coding RNAs in cancer: mechanistic insights and therapeutic implications. Pathology - Res. Pract. 266, 155745. 10.1016/j.prp.2024.155745 39637712

[B81] Kadian L. K. Verma D. Lohani N. Yadav R. Ranga S. Gulshan G. (2024). Long non-coding RNAs in cancer: multifaceted roles and potential targets for immunotherapy. Mol. Cell Biochem. 479, 3229–3254. 10.1007/s11010-024-04933-1 38413478

[B82] Kathuria-Prakash N. Dave P. Garcia L. Brown P. Drakaki A. (2024). MicroRNAs in genitourinary malignancies: an exciting frontier of cancer diagnostics and therapeutics. Int. J. Mol. Sci. 25, 9499. 10.3390/ijms25179499 39273446 PMC11394927

[B83] Kaya M. Tuna G. Yilmaz S. (2024). “Relevance of small molecule inhibitors in advancing cancer therapeutics,” in Small Molecules for Cancer Treatment. Editors Pathak S. Banerjee A. Duttaroy A. K. (Singapore: Springer Nature), 103–117. 10.1007/978-981-96-0301-5_5

[B84] Keoh L. Q. Chiu C.-F. Ramasamy T. S. (2025). Metabolic plasticity and cancer stem cell metabolism: exploring the Glycolysis-OXPHOS switch as a mechanism for resistance and tumorigenesis. Stem Cell Rev. Rep. 21, 2446–2468. 10.1007/s12015-025-10956-y 40880049 PMC12504402

[B85] Khan B. D. Nhi N. T. Y. Nhan T. N. T. Khuong P. D. (2025). Cancer cell dormancy: an update to 2025. Biomed. Res. Ther. 12, 7559–7575. 10.15419/ttm97s19

[B86] Kim H. I. Park J. Zhu Y. Wang X. Han Y. Zhang D. (2024). Recent advances in extracellular vesicles for therapeutic cargo delivery. Exp. Mol. Med. 56, 836–849. 10.1038/s12276-024-01201-6 38556545 PMC11059217

[B87] Kreso A. Dick J. E. (2014). Evolution of the cancer stem cell model. Cell Stem Cell 14, 275–291. 10.1016/j.stem.2014.02.006 24607403

[B88] Krützfeldt J. Kuwajima S. Braich R. Rajeev K. G. Pena J. Tuschl T. (2007). Specificity, duplex degradation and subcellular localization of antagomirs. Nucleic Acids Res. 35, 2885–2892. 10.1093/nar/gkm024 17439965 PMC1888827

[B89] Lamouille S. Xu J. Derynck R. (2014). Molecular mechanisms of epithelial–mesenchymal transition. Nat. Rev. Mol. Cell Biol. 15, 178–196. 10.1038/nrm3758 24556840 PMC4240281

[B90] Lee H. Kim B. Park J. Park S. Yoo G. Yum S. (2025). Cancer stem cells: landscape, challenges and emerging therapeutic innovations. Sig Transduct. Target Ther. 10, 248. 10.1038/s41392-025-02360-2 PMC12322150 40759634

[B91] Leucci E. (2026). lncRNAs as targetable nodes of oncogenic networks underlying cancer progression and therapy resistance. Annu. Rev. Cancer Biol. 10, 297–311. 10.1146/annurev-cancerbio-071124-012245

[B92] Li X. Mertens-Talcott S. U. Zhang S. Kim K. Ball J. Safe S. (2010). MicroRNA-27a indirectly regulates estrogen receptor α expression and hormone responsiveness in MCF-7 breast cancer cells. Endocrinology 151, 2462–2473. 10.1210/en.2009-1150 20382698 PMC2875816

[B93] Li W. J. Wang Y. Liu R. Kasinski A. L. Shen H. Slack F. J. (2021). MicroRNA-34a: potent tumor suppressor, cancer stem cell inhibitor, and potential anticancer therapeutic. Front. Cell Dev. Biol. 9, 640587. 10.3389/fcell.2021.640587 33763422 PMC7982597

[B94] Li Y. He Y. Chen Y. He Z. Yang F. Xing C. (2023). Contribution of microRNA-30d to the prevention of the thyroid cancer occurrence and progression: mechanism and implications. Apoptosis 28, 576–593. 10.1007/s10495-023-01809-5 36695983

[B95] Li J. Hu J. Yang Y. Zhang H. Liu Y. Fang Y. (2025a). Drug resistance in cancer: molecular mechanisms and emerging treatment strategies. Mol. Biomed. 6, 111. 10.1186/s43556-025-00352-w 41247642 PMC12623568

[B96] Li J. Peng J. Wang J. Chen Z. (2025b). The dual role of exosomes in the tumor microenvironment: from pro-tumorigenic signaling to immune modulation. Med Res. 1, 257–284. 10.1002/mdr2.70022

[B97] Li S. Zhang Y. Tong H. Sun H. Liao H. Li Q. (2025c). Metabolic regulation of immunity in the tumor microenvironment. Cell Rep. 44, 116463. 10.1016/j.celrep.2025.116463 41129316

[B98] Liang H. Zhou B. Li P. Zhang X. Zhang S. Zhang Y. (2025). Stemness regulation in prostate cancer: prostate cancer stem cells and targeted therapy. Ann. Med. 57, 2442067. 10.1080/07853890.2024.2442067 39711287 PMC11703425

[B99] Liao J. Chen B. Zhu Z. Du C. Gao S. Zhao G. (2023). Long noncoding RNA (lncRNA) H19: an essential developmental regulator with expanding roles in cancer, stem cell differentiation, and metabolic diseases. Genes Dis. 10, 1351–1366. 10.1016/j.gendis.2023.02.008 37397543 PMC10311118

[B100] Liu C. Chen Z. Fang J. Xu A. Zhang W. Wang Z. (2015). H19-derived miR-675 contributes to bladder cancer cell proliferation by regulating p53 activation. Tumour Biol. 37, 263–270. 10.1007/s13277-015-3779-2 26198047 PMC4841850

[B101] Liu C. Luo Y. Zhou H. Lin M. Zang D. Chen J. (2025a). Immune cell-derived exosomal non-coding RNAs in tumor microenvironment: biological functions and potential clinical applications. Chin. J. Cancer Res. 37, 250–267. 10.21147/j.issn.1000-9604.2025.02.10 40353080 PMC12062983

[B102] Liu G. Liu Q. Jia L. Chai Z. Jing L. Xu F. (2025b). Exosomal circRNAs: key modulators in breast cancer progression. Cell Death Discov. 11, 196. 10.1038/s41420-025-02494-w 40274787 PMC12022065

[B103] Liu T. M. Tew W. Yang Z. Lim B. Hui J. H. P. Lee E. H. (2025c). Understanding the molecular basis of mesenchymal stem cell stemness: implications for clinical applications. Cell Death Dis. 16, 778. 10.1038/s41419-025-08094-x 41184233 PMC12583528

[B104] Liu F. Beck S. Yang L. Luo H. Zhang K. (2026). Advancing AI for multi-omics and clinical data integration in basic and translational cancer research. Nat. Rev. Cancer 26, 497–512. 10.1038/s41568-026-00922-2 42014628

[B105] Loh J.-J. Ma S. (2024). Hallmarks of cancer stemness. Cell Stem Cell 31, 617–639. 10.1016/j.stem.2024.04.004 38701757

[B106] Lou L. Cao Y. Ma Z. Ji B. Liu S. Mizuno K. (2025). The key role of cellular plasticity in the development of colorectal cancer. Transl. Res. 285–286, 40–47. 10.1016/j.trsl.2025.12.002 41352528

[B107] Lu L.-F. Boldin M. P. Chaudhry A. Lin L.-L. Taganov K. D. Hanada T. (2010). Function of miR-146a in controlling Treg cell-mediated regulation of Th1 responses. Cell 142, 914–929. 10.1016/j.cell.2010.08.012 20850013 PMC3049116

[B108] Lu J. Guo J. Liu J. Mao X. Xu K. (2022). Long non-coding RNA MALAT1: a key player in liver diseases. Front. Med. 8, 734643. 10.3389/fmed.2021.734643 35145971 PMC8821149

[B109] Luo W. Liu X. Han Y. Duan Y. Yu C. Kong N. (2025). Engineered RNA devices for In Vivo targeted therapeutics via advanced delivery systems. Aggregate 6, e70191. 10.1002/agt2.70191

[B110] Ma B. Wang S. Wu W. Shan P. Chen Y. Meng J. (2023). Mechanisms of circRNA/lncRNA-miRNA interactions and applications in disease and drug research. Biomed. and Pharmacother. 162, 114672. 10.1016/j.biopha.2023.114672 37060662

[B111] Mahamed R. Monchusi B. Penny C. Mirza S. (2025). Cancer-derived exosomes: mediators of immune crosstalk and emerging targets for immunotherapy. Front. Immunol. 16, 1679934. 10.3389/fimmu.2025.1679934 41142789 PMC12546359

[B112] Mai Y. Su J. Yang C. Xia C. Fu L. (2023). The strategies to cure cancer patients by eradicating cancer stem-like cells. Mol. Cancer 22, 171. 10.1186/s12943-023-01867-y 37853413 PMC10583358

[B113] Mamun M. A. Mannoor K. Cao J. Qadri F. Song X. (2020). SOX2 in cancer stemness: tumor malignancy and therapeutic potentials. J. Mol. Cell Biol. 12, 85–98. 10.1093/jmcb/mjy080 30517668 PMC7109607

[B114] Mangraviti N. Castelli S. (2025). Non-coding RNAs at the intersection of epigenetics and cancer metabolism. Front. Epigenet. Epigenom. 3, 1699969. 10.3389/freae.2025.1699969

[B115] Mariner P. D. Walters R. D. Espinoza C. A. Drullinger L. F. Wagner S. D. Kugel J. F. (2008). Human alu RNA is a modular transacting repressor of mRNA transcription during heat shock. Mol. Cell 29, 499–509. 10.1016/j.molcel.2007.12.013 18313387

[B116] Masoudi M. Moti D. Masoudi R. Auwal A. Hossain M. M. Pronoy T. U. H. (2024). Metabolic adaptations in cancer stem cells: a key to therapy resistance. Biochimica Biophysica Acta Mol. Basis Dis. 1870, 167164. 10.1016/j.bbadis.2024.167164 38599259

[B117] Mattick J. S. Amaral P. P. Carninci P. Carpenter S. Chang H. Y. Chen L.-L. (2023). Long non-coding RNAs: definitions, functions, challenges and recommendations. Nat. Rev. Mol. Cell Biol. 24, 430–447. 10.1038/s41580-022-00566-8 36596869 PMC10213152

[B118] Meng F. Henson R. Wehbe-Janek H. Ghoshal K. Jacob S. T. Patel T. (2007). MicroRNA-21 regulates expression of the PTEN tumor suppressor gene in human hepatocellular cancer. Gastroenterology 133, 647–658. 10.1053/j.gastro.2007.05.022 17681183 PMC4285346

[B119] Mir R. Jan U. Barnawi J. Algehainy N. A. Jalal M. M. Altayar M. A. (2025). Extracellular derived-exosomal micrornas in pancreatic cancer: investigating their diagnostic importance and potential targets for the prevention and treatment in pancreatic cancer. Front. Oncol. 15, 1669213. 10.3389/fonc.2025.1669213 41347080 PMC12672277

[B120] Murakami Y. Yasuda T. Saigo K. Urashima T. Toyoda H. Okanoue T. (2006). Comprehensive analysis of microRNA expression patterns in hepatocellular carcinoma and non-tumorous tissues. Oncogene 25, 2537–2545. 10.1038/sj.onc.1209283 16331254

[B121] Nademi N. Ozfiliz-Kilbas P. (2026). The CeRNA role of HOTAIR: sponging MiRs to promote chemoresistance. Mol. Biol. Rep. 53, 319. 10.1007/s11033-026-11490-x 41569464

[B122] Nazari M. Babakhanzadeh E. Mollazadeh A. Ahmadzade M. Mohammadi Soleimani E. Hajimaqsoudi E. (2024). HOTAIR in cancer: diagnostic, prognostic, and therapeutic perspectives. Cancer Cell Int. 24, 415. 10.1186/s12935-024-03612-x 39702144 PMC11660992

[B123] Nemeth K. Bayraktar R. Ferracin M. Calin G. A. (2024). Non-coding RNAs in disease: from mechanisms to therapeutics. Nat. Rev. Genet. 25, 211–232. 10.1038/s41576-023-00662-1 37968332

[B124] Nestor M. S. Hetzel J. Awad N. Bhupalam V. Lu P. Molyneaux M. (2024). Novel injectable polypeptide nanoparticle encapsulated siRNA targeting TGF-β1 and COX-2 for localized fat reduction I: preclinical in vitro and animal models. J. Cosmet. Dermatology 23, 3133–3143. 10.1111/jocd.16535 PMC13649109 39166716

[B125] Ng C. X. Lee S. Y. Yap X. Y. Wong Y. H. Loh J. S. Ang K. P. (2025). Epigenetic reprogramming as the nexus of cancer stemness and therapy resistance: implications for biomarker discovery. Discov. Onc 16, 2220. 10.1007/s12672-025-04085-8 PMC12738525 41258574

[B126] Nguyen H.-M. Alexander K. E. Collinge M. Hickey J. C. Lanz T. A. Li J. (2025). mRNA-LNPs induce immune activation and cytokine release in human whole blood assays across diverse health conditions. Mol. Ther. 33, 2872–2885. 10.1016/j.ymthe.2024.12.019 39673130 PMC12172169

[B127] Nwokolo G. C. Ganesan T. S. Pors K. Falconer R. A. Smarakan S. (2025). Cancer stem cells in focus: deciphering the dynamic functional landscape of stemness in cancer. Biochimica Biophysica Acta Rev. Cancer 1880, 189440. 10.1016/j.bbcan.2025.189440 40912376

[B128] Ohanian M. Ashizawa A. T. Garcia-Manero G. Pemmaraju N. Kadia T. Jabbour E. (2018). Liposomal Grb2 antisense oligodeoxynucleotide (BP1001) in patients with refractory or relapsed haematological malignancies: a single-centre, open-label, dose-escalation, phase 1/1b trial. Lancet Haematol. 5, e136–e146. 10.1016/S2352-3026(18)30021-8 29550383 PMC11840760

[B129] Ou R. Zhu L. Zhao L. Li W. Tao F. Lu Y. (2019). HPV16 E7-induced upregulation of KDM2A promotes cervical cancer progression by regulating miR-132-radixin pathway. J. Cell Physiol. 234, 2659–2671. 10.1002/jcp.27080 30132864

[B130] Ouyang Q. Cui Y. Yang S. Wei W. Zhang M. Zeng J. (2020). lncRNA MT1JP suppresses biological activities of breast cancer cells in vitro and in vivo by regulating the miRNA-214/RUNX3 axis. Onco Targets Ther. 13, 5033–5046. 10.2147/OTT.S241503 32581560 PMC7280253

[B131] O’Brien J. Hayder H. Zayed Y. Peng C. (2018). Overview of MicroRNA biogenesis, mechanisms of actions, and circulation. Front. Endocrinol. 9, 402. 10.3389/fendo.2018.00402 PMC6085463 30123182

[B132] Paasch T. P. Olesen M. T. J. García-Rodríguez J. L. Assmus A. M. Fenton R. A. Kjems J. (2025). ciRS-7 expression is epigenetically regulated in cancer cells across human adenocarcinomas. PLOS Genet. 21, e1011726. 10.1371/journal.pgen.1011726 40455824 PMC12162099

[B133] Pająk W. Kleinrok J. Pec J. Michno K. Wojtas J. Badach M. (2025). Micro RNA in colorectal cancer—potential diagnostic and prognostic markers—an updated review. Int. J. Mol. Sci. 26, 8615. 10.3390/ijms26178615 40943532 PMC12429582

[B134] Pallante P. Visone R. Ferracin M. Ferraro A. Berlingieri M. T. Troncone G. (2006). MicroRNA deregulation in human thyroid papillary carcinomas. Endocrine-Related Cancer 13, 497–508. 10.1677/erc.1.01209 16728577

[B135] Palm W. (2021). Metabolic plasticity allows cancer cells to thrive under nutrient starvation. Proc. Natl. Acad. Sci. U. S. A. 118, e2102057118. 10.1073/pnas.2102057118 33722932 PMC8043109

[B136] Pan T. Yang C.-H. Zhao K. Sun Y.-L. Rao Z.-Y. Qu W.-Q. (2025). Biomimetic artificial enveloped viral vectors: overcoming immune barriers for re-administration and long-term gene therapy. Cell Biomater. 1, 100143. 10.1016/j.celbio.2025.100143

[B137] Pandey S. Yadav P. (2025). Exploring the therapeutic potential of microRNAs: targeted gene regulation strategies for enhanced cancer therapy. J. Genet. Eng. Biotechnol. 23, 100556. 10.1016/j.jgeb.2025.100556 41386822 PMC12405681

[B138] Pandey K. Jha S. Chaliha L. B. Gosai H. Patel R. (2026). Nanomaterial-enabled RNA therapeutics: bridging delivery barriers to clinical translation. Front. Nanotechnol. 8, 1860221. 10.3389/fnano.2026.1860221

[B139] Parkins E. V. Brager D. H. Rymer J. K. Burwinkel J. M. Rojas D. Tiwari D. (2023). Mir324 knockout regulates the structure of dendritic spines and impairs hippocampal long-term potentiation. Sci. Rep. 13, 21919. 10.1038/s41598-023-49134-w 38082035 PMC10713680

[B140] Parvizi M. Vaezi M. Jeddi F. Bakhshandeh M. Eghdam-Zamiri R. Mobaraki-Asl N. (2025). The role and diagnostic value of deregulated miRNAs in cervical cancer. Discov. Onc 16, 922. 10.1007/s12672-025-02744-4 PMC12104134 40413660

[B141] Paul R. Dorsey J. F. Fan Y. (2022). Cell plasticity, senescence, and quiescence in cancer stem cells: biological and therapeutic implications. Pharmacol. Ther. 231, 107985. 10.1016/j.pharmthera.2021.107985 34480963 PMC8844041

[B142] Peng J. Liu W. Tian J. Shu Y. Zhao R. Wang Y. (2025). Non-coding RNAs as key regulators of epithelial-mesenchymal transition in breast cancer. Front. Cell Dev. Biol. 13, 1544310. 10.3389/fcell.2025.1544310 40201201 PMC11975958

[B143] Peter M. E. (2009). Let-7 and miR-200 microRNAs. Cell Cycle 8, 843–852. 10.4161/cc.8.6.7907 19221491 PMC2688687

[B144] Pisignano G. Michael D. C. Visal T. H. Pirlog R. Ladomery M. Calin G. A. (2023). Going circular: history, present, and future of circRNAs in cancer. Oncogene 42, 2783–2800. 10.1038/s41388-023-02780-w 37587333 PMC10504067

[B145] Proia T. A. Singh M. Woessner R. Carnevalli L. Bommakanti G. Magiera L. (2020). STAT3 antisense oligonucleotide remodels the suppressive tumor microenvironment to enhance immune activation in combination with Anti–PD-L1. Clin. Cancer Res. 26, 6335–6349. 10.1158/1078-0432.CCR-20-1066 32943458

[B146] Qazi R. e. M. Rehman F. U. Rehman A. Mian A. A. (2026). Engineered exosome based immunomodulation therapies: advancements and clinical applications. Nano Biomed. Eng. 18, 100005. 10.1016/j.nbe.2025.100005

[B147] Querfeld C. Foss F. M. Kim Y. H. Pinter-Brown L. William B. M. Porcu P. (2018). Phase 1 trial of Cobomarsen, an inhibitor of Mir-155, in cutaneous T cell lymphoma. Blood 132, 2903. 10.1182/blood-2018-99-119861

[B148] Rashed W. M. Hammad A. M. Saad A. M. Shohdy K. S. (2019). MicroRNA as a diagnostic biomarker in childhood acute lymphoblastic leukemia; *systematic* review, meta-analysis and recommendations. Crit. Rev. Oncology/Hematology 136, 70–78. 10.1016/j.critrevonc.2019.02.008 30878131

[B149] Ratti M. Lampis A. Ghidini M. Salati M. Mirchev M. B. Valeri N. (2020). MicroRNAs (miRNAs) and long non-coding RNAs (lncRNAs) as new tools for cancer therapy: first steps from bench to bedside. Target Oncol. 15, 261–278. 10.1007/s11523-020-00717-x 32451752 PMC7283209

[B150] Ravindran F. Koroth J. Manjunath M. Narayan S. Choudhary B. (2021). Curcumin derivative ST09 modulates the miR-199a-5p/DDR1 axis and regulates proliferation and migration in ovarian cancer cells. Sci. Rep. 11, 23025. 10.1038/s41598-021-02454-1 34837026 PMC8626492

[B151] Reddy R. A. Naing A. Lopez-Berestein G. Rodriguez-Aguayo C. Momin H. A. Madhyannapu A. (2025). EphA2 siRNA in DOPC nanoliposomes (EPHARNA): a phase I clinical trial in patients with solid tumors. J. Clin. Oncol. 43, 3086. 10.1200/JCO.2025.43.16_suppl.3086

[B152] Reis E. M. M. Bassères D. S. S. (2026). Long noncoding RNAs in tumor stemness: emerging mechanisms and therapeutic opportunities. Front. Genet. 17, 1772938. 10.3389/fgene.2026.1772938 41822755 PMC12975461

[B153] Rinkenbaugh A. L. Baldwin A. S. (2016). The NF-κB pathway and cancer stem cells. Cells 5, 16. 10.3390/cells5020016 27058560 PMC4931665

[B154] Rouault C. D. Charafe-Jauffret E. Ginestier C. (2025). The interplay of DNA damage, epigenetics and tumour heterogeneity in driving cancer cell fitness. Nat. Commun. 16, 8733. 10.1038/s41467-025-64445-4 41028732 PMC12484684

[B155] Saba R. Gushue S. Huzarewich R. L. C. H. Manguiat K. Medina S. Robertson C. (2012). MicroRNA 146a (miR-146a) is over-expressed during prion disease and modulates the innate immune response and the microglial activation state. PLoS One 7, e30832. 10.1371/journal.pone.0030832 22363497 PMC3281888

[B156] Sadeghi Z. Malekzadeh M. Sharifi M. Hashemibeni B. (2025). The role of miR-16 and miR-34a family in the regulation of cancers: a review. Heliyon 11, e42733. 10.1016/j.heliyon.2025.e42733 40061926 PMC11889592

[B157] Saiselet M. Pita J. M. Augenlicht A. Dom G. Tarabichi M. Fimereli D. (2016). miRNA expression and function in thyroid carcinomas: a comparative and critical analysis and a model for other cancers. Oncotarget 7, 52475–52492. 10.18632/oncotarget.9655 27248468 PMC5239568

[B158] Schultheis B. Strumberg D. Kuhlmann J. Wolf M. Link K. Seufferlein T. (2020). Safety, efficacy and pharcacokinetics of targeted therapy with the liposomal RNA interference therapeutic Atu027 combined with gemcitabine in patients with pancreatic adenocarcinoma. A randomized phase Ib/IIa study. Cancers 12, 3130. 10.3390/cancers12113130 33114652 PMC7693593

[B159] Sergeeva A. Davydova K. Perenkov A. Vedunova M. (2023). Mechanisms of human DNA methylation, alteration of methylation patterns in physiological processes and oncology. Gene 875, 147487. 10.1016/j.gene.2023.147487 37211289

[B160] Shang T. Jia Z. Li J. Cao H. Xu H. Cong L. (2025). Unraveling the triad of hypoxia, cancer cell stemness, and drug resistance. J. Hematol. Oncol. 18, 32. 10.1186/s13045-025-01684-4 40102937 PMC11921735

[B161] Shi J. Liu Y. Xu X. Zhang W. Yu T. Jia J. (2015). Deubiquitinase USP47/UBP64E regulates β-Catenin ubiquitination and degradation and plays a positive role in wnt signaling. Mol. Cell Biol. 35, 3301–3311. 10.1128/MCB.00373-15 26169834 PMC4561727

[B162] Shi Q. Xue C. Zeng Y. Yuan X. Chu Q. Jiang S. (2024). Notch signaling pathway in cancer: from mechanistic insights to targeted therapies. Sig Transduct. Target Ther. 9, 128. 10.1038/s41392-024-01828-x PMC11128457 38797752

[B163] Shinde P. P. Chitkara D. Mittal A. (2024). Downregulation of microRNA-29b in cancer and fibrosis: molecular insights and clinical implications. Drug Discov. Today 29, 104190. 10.1016/j.drudis.2024.104190 39322175

[B164] Singh V. K. Rajak N. Singh Y. Singh A. K. Giri R. Garg N. (2024). Role of MicroRNA-21 in prostate cancer progression and metastasis: molecular mechanisms to therapeutic targets. Ann. Surg. Oncol. 31, 4795–4808. 10.1245/s10434-024-15453-z 38758485

[B165] Singh K. Jain D. Sethi P. Gupta J. K. Dubey A. Al Noman A. (2025). Advances in viral vector-based delivery systems for gene therapy: a comprehensive review. 3 Biotech. 15, 196. 10.1007/s13205-025-04366-7 40454374 PMC12125461

[B166] Singh M. Priya K. Nagar A. Nalavade R. (2025). Emerging landscape of lncRNA-miRNA interactions as architects of gene expression patterns. Mol. Biol. Rep. 52, 810. 10.1007/s11033-025-10906-4 40778999

[B167] Singh A. Tekade M. Nagaraja S. Bharti A. Tekade R. K. (2026). Advancements in RNA-based therapies from bench to bedside. Npj Drug Discov. 3, 4. 10.1038/s44386-025-00037-y 42380588 PMC13252867

[B168] Siomi M. C. Sato K. Pezic D. Aravin A. A. (2011). PIWI-interacting small RNAs: the vanguard of genome defence. Nat. Rev. Mol. Cell Biol. 12, 246–258. 10.1038/nrm3089 21427766

[B169] Song Y. (2026). Challenges and opportunities in RNA-centered therapeutics. Nat. Chem. Biol. 22, 847–854. 10.1038/s41589-026-02227-9 42225945

[B170] Song J. Yang P. Chen C. Ding W. Tillement O. Bai H. (2025). Targeting epigenetic regulators as a promising avenue to overcome cancer therapy resistance. Sig Transduct. Target Ther. 10, 219. 10.1038/s41392-025-02266-z PMC12271501 40675967

[B171] Soragni A. Knudsen E. S. O’Connor T. N. Tognon C. E. Tyner J. W. Gini B. (2025). Acquired resistance in cancer: towards targeted therapeutic strategies. Nat. Rev. Cancer 25, 613–633. 10.1038/s41568-025-00824-9 40461793 PMC12307123

[B172] Sorokin L. (2026). Biochemical signals from the extracellular matrix in inflammation and tumour immunology. Nat. Rev. Immunol. 26, 350–366. 10.1038/s41577-025-01248-0 41501478

[B173] Statello L. Guo C.-J. Chen L.-L. Huarte M. (2021). Gene regulation by long non-coding RNAs and its biological functions. Nat. Rev. Mol. Cell Biol. 22, 96–118. 10.1038/s41580-020-00315-9 33353982 PMC7754182

[B174] Sultana Z. Ahamed A. Hoda M. Ali S. Hoque M. (2026). Prospects and challenges in intracellular delivery of therapeutic proteins. Pharmacol. Rep. 78, 679–706. 10.1007/s43440-026-00855-5 42084823

[B175] Tachiwana H. Saitoh N. (2021). Nuclear long non-coding RNAs as epigenetic regulators in cancer. Curr. Med. Chem. 28, 5098–5109. 10.2174/0929867328666210215114506 33588720

[B176] Tan Y. Qin S. Zhang Z. Liu Y. Zhou L. Li B. (2025). Unraveling the underlying mechanisms of cancer stem cells in therapeutic resistance for optimizing treatment strategies. MedComm Oncol. 4, e70009. 10.1002/mog2.70009

[B177] Tripathi V. Shen Z. Chakraborty A. Giri S. Freier S. M. Wu X. (2013). Long noncoding RNA MALAT1 controls cell cycle progression by regulating the expression of oncogenic transcription factor B-MYB. PLoS Genet. 9, e1003368. 10.1371/journal.pgen.1003368 23555285 PMC3605280

[B178] Valinezhad Orang A. Safaralizadeh R. Kazemzadeh-Bavili M. (2014). Mechanisms of miRNA-Mediated gene regulation from common downregulation to mRNA-Specific upregulation. Int. J. Genomics 2014, 970607. 10.1155/2014/970607 25180174 PMC4142390

[B179] van Zandwijk N. Pavlakis N. Kao S. Clarke S. Lee A. Brahmbhatt H. (2015). P1.02 - MesomiR 1: a phase I study of TargomiRs in patients with refractory malignant pleural mesothelioma (MPM) and lung cancer (NSCLC). Ann. Oncol. 26, ii16. 10.1093/annonc/mdv090.2

[B180] Visvader J. E. Lindeman G. J. (2012). Cancer stem cells: current status and evolving complexities. Cell Stem Cell 10, 717–728. 10.1016/j.stem.2012.05.007 22704512

[B181] Viteri S. Rosell R. (2018). An innovative mesothelioma treatment based on miR-16 mimic loaded EGFR targeted minicells (TargomiRs). Transl. Lung Cancer Res. 7, S1–S4. 10.21037/tlcr.2017.12.01 29531894 PMC5835633

[B182] Wahab A. Siddique H. R. (2025). An update understanding of stemness and chemoresistance of prostate cancer. Expert Rev. Anticancer Ther. 25, 215–228. 10.1080/14737140.2025.2466680 39935028

[B183] Wang D. Ding L. Wang L. Zhao Y. Sun Z. Karnes R. J. (2015). LncRNA MALAT1 enhances oncogenic activities of EZH2 in castration-resistant prostate cancer. Oncotarget 6, 41045–41055. 10.18632/oncotarget.5728 26516927 PMC4747388

[B184] Wang M. Yu F. Wu W. Zhang Y. Chang W. Ponnusamy M. (2017). Circular RNAs: a novel type of non-coding RNA and their potential implications in antiviral immunity. Int. J. Biol. Sci. 13, 1497–1506. 10.7150/ijbs.22531 29230098 PMC5723916

[B185] Wang Z. Wang H. Zhou S. Mao J. Zhan Z. Duan S. (2024). miRNA interplay: mechanisms and therapeutic interventions in cancer. MedComm Oncol. 3, e93. 10.1002/mog2.93

[B186] Wang C. Bai M. Liu X. Li Z. Wang H. Guo S. (2025a). Molecular roles of microRNA-21 and exosomal miR-21 in gastrointestinal cancers: diagnostic, therapeutic, and drug resistance insights. Front. Mol. Biosci. 12, 1697875. 10.3389/fmolb.2025.1697875 41476448 PMC12747839

[B187] Wang H. Li J. Du F. Deng H. (2025b). Cancer stem cells: bridging microenvironmental interactions and clinical therapy. Clin. Transl. Med. 15, e70406. 10.1002/ctm2.70406 40665579 PMC12264091

[B188] Wang S. Shu J. Wang N. He Z. (2025c). Exosomal non-coding RNAs: mediators of crosstalk between cancer and cancer stem cells. Cell Death Discov. 11, 434. 10.1038/s41420-025-02726-z 41053039 PMC12501049

[B189] Wang S. Weissman D. Dong Y. (2025d). RNA chemistry and therapeutics. Nat. Rev. Drug Discov. 24, 828–851. 10.1038/s41573-025-01237-x 40659813 PMC12514552

[B190] Wang Y. Fu M. Zheng Z. Feng J. Zhang C. (2025e). Small nucleolar RNAs: biological functions and diseases. MedComm 6 (2020), e70257. 10.1002/mco2.70257 40584410 PMC12205218

[B191] Wang Q. ten Dijke P. Fan C. (2026). Deciphering the regulatory landscape of enhancer RNAs in health and disease. Sig Transduct. Target Ther. 11, 29. 10.1038/s41392-025-02436-z PMC12852191 41605892

[B192] Wang Y. Bao G. Li H. Li X. Lv L. (2026). The role of non-coding RNA-mediated autophagy in Alzheimer’s disease. Brain Res. Bull. 239, 111860. 10.1016/j.brainresbull.2026.111860 41933660

[B193] Watrowski R. Kostov S. Palumbo M. Rosati A. Sparić R. Alkatout I. (2025). Non-coding RNAs (microRNAs, lncRNAs, circRNAs) in adenomyosis: a systematic review of mechanistic and translational evidence. Int. J. Mol. Sci. 26, 10713. 10.3390/ijms262110713 41226749 PMC12611009

[B194] Winkle M. El-Daly S. M. Fabbri M. Calin G. A. (2021). Noncoding RNA therapeutics — challenges and potential solutions. Nat. Rev. Drug Discov. 20, 629–651. 10.1038/s41573-021-00219-z 34145432 PMC8212082

[B195] Włodarczyk M. Maryńczak K. Burzyński J. Włodarczyk J. Basak J. Fichna J. (2025). The role of miRNAs in the pathogenesis, diagnosis, and treatment of colorectal cancer and colitis-associated cancer. Clin. Exp. Med. 25, 86. 10.1007/s10238-025-01582-6 40091000 PMC11911275

[B196] Wu X. Que H. Li Q. Wei X. (2025). Wnt/β-catenin mediated signaling pathways in cancer: recent advances, and applications in cancer therapy. Mol. Cancer 24, 171. 10.1186/s12943-025-02363-1 40495229 PMC12150590

[B197] Xia Y. Pei T. Zhao J. Wang Z. Shen Y. Yang Y. (2024). Long noncoding RNA H19: functions and mechanisms in regulating programmed cell death in cancer. Cell Death Discov. 10, 76. 10.1038/s41420-024-01832-8 38355574 PMC10866971

[B198] Xiao J. Zhang L. Su R. Zhao B. Dang Y. Zhao C. (2025). MicroRNAs in breast cancer—new frontiers in diagnosis, targeted therapy, and prognosis assessment. Front. Oncol. 15, 1529907. 10.3389/fonc.2025.1529907 40958878 PMC12433965

[B199] Xie N. Cui H. Banerjee S. Tan Z. Salomao R. Fu M. (2014). miR-27a regulates inflammatory response of macrophages by targeting IL-10. J. Immunol. 193, 327–334. 10.4049/jimmunol.1400203 24835395 PMC4065847

[B200] Xue C. Chu Q. Shi Q. Zeng Y. Lu J. Li L. (2025). Wnt signaling pathways in biology and disease: mechanisms and therapeutic advances. Sig Transduct. Target Ther. 10, 106. 10.1038/s41392-025-02142-w PMC11968978 40180907

[B201] Yamakuchi M. Ferlito M. Lowenstein C. J. (2008). miR-34a repression of SIRT1 regulates apoptosis. Proc. Natl. Acad. Sci. U. S. A. 105, 13421–13426. 10.1073/pnas.0801613105 18755897 PMC2533205

[B202] Yan Y. Liu S. Wen J. He Y. Duan C. Nabavi N. (2025). Advances in RNA-based cancer therapeutics: pre-clinical and clinical implications. Mol. Cancer 24, 251. 10.1186/s12943-025-02463-y 41068915 PMC12512572

[B203] Yang F. Huo X. Yuan S. Zhang L. Zhou W. Wang F. (2013). Repression of the long noncoding RNA-LET by histone deacetylase 3 contributes to hypoxia-mediated metastasis. Mol. Cell 49, 1083–1096. 10.1016/j.molcel.2013.01.010 23395002

[B204] Yang Y. Razak S. R. A. Ismail I. S. Ma Y. Yunus M. A. (2025). Molecular mechanisms of miR-192 in cancer: a biomarker and therapeutic target. Cancer Cell Int. 25, 94. 10.1186/s12935-025-03666-5 40087755 PMC11908092

[B205] Yang L. Li C. Jiang X. Yuan Y. Li C. Zhou Q. (2026). Functional roles and mechanisms of circRNA-protein interactions in cancer progression and tumor immune regulation. Front. Immunol. 17, 1771949. 10.3389/fimmu.2026.1771949 41756268 PMC12932422

[B206] Yin Y. Shen X. (2023). Noncoding RNA-chromatin association: functions and mechanisms. Fundam. Res. 3, 665–675. 10.1016/j.fmre.2023.03.006 38933302 PMC11197541

[B207] Ying S.-Y. Chang D. C. Lin S.-L. (2008). The MicroRNA (miRNA): overview of the RNA genes that modulate gene function. Mol. Biotechnol. 38, 257–268. 10.1007/s12033-007-9013-8 17999201 PMC7091389

[B208] Yu Z. Li Y. Fan H. Liu Z. Pestell R. G. (2012). miRNAs regulate stem cell self-renewal and differentiation. Front. Genet. 3, 191. 10.3389/fgene.2012.00191 23056008 PMC3457037

[B209] Yu X. Zhao H. Wang R. Chen Y. Ouyang X. Li W. (2024). Cancer epigenetics: from laboratory studies and clinical trials to precision medicine. Cell Death Discov. 10, 28. 10.1038/s41420-024-01803-z 38225241 PMC10789753

[B210] Yuan J. Zhang P. Cui Y. Wang J. Skogerbø G. Huang D.-W. (2016). Computational identification of piRNA targets on mouse mRNAs. Bioinformatics 32, 1170–1177. 10.1093/bioinformatics/btv729 26677964

[B211] Zafar S. Hafeez A. Shah H. Mutiullah I. Ali A. Khan K. (2025). Emerging biomarkers for early cancer detection and diagnosis: challenges, innovations, and clinical perspectives. Eur. J. Med. Res. 30, 760. 10.1186/s40001-025-03003-6 40826140 PMC12359849

[B212] Zarlashat Y. Halász J. Dósa E. (2025). Dysregulation of MicroRNAs in hepatocellular carcinoma: targeting oncogenic signaling pathways for innovative therapies. Int. J. Mol. Sci. 26, 8365. 10.3390/ijms26178365 40943288 PMC12428066

[B213] Zeng Z. Fu M. Hu Y. Wei Y. Wei X. Luo M. (2023). Regulation and signaling pathways in cancer stem cells: implications for targeted therapy for cancer. Mol. Cancer 22, 172. 10.1186/s12943-023-01877-w 37853437 PMC10583419

[B214] Zhang X. Ng W.-L. Wang P. Tian L. Werner E. Wang H. (2012). MicroRNA-21 modulates the levels of reactive oxygen species levels by targeting SOD3 and TNFα. Cancer Res. 72, 4707–4713. 10.1158/0008-5472.CAN-12-0639 22836756 PMC3445705

[B215] Zhang D. Zhou J. Gao J. Wu R.-Y. Huang Y.-L. Jin Q.-W. (2019). Targeting snoRNAs as an emerging method of therapeutic development for cancer. Am. J. Cancer Res. 9, 1504–1516. Available online at: https://pmc.ncbi.nlm.nih.gov/articles/PMC6726984/. 31497339 PMC6726984

[B216] Zhang L. Liao Y. Tang L. (2019). MicroRNA-34 family: a potential tumor suppressor and therapeutic candidate in cancer. J. Exp. Clin. Cancer Res. 38, 53. 10.1186/s13046-019-1059-5 30717802 PMC6360685

[B217] Zhang Q. Zhu Y. Cao X. Tan W. Yu J. Lu Y. (2023). The epigenetic regulatory mechanism of PIWI/piRNAs in human cancers. Mol. Cancer 22, 45. 10.1186/s12943-023-01749-3 36882835 PMC9990219

[B218] Zhang J. Wang X. Feng X. Chen Y. Yu K. Jiang Z. (2025). Roles and mechanisms of piRNAs in self-renewal and differentiation of germline stem cells. Differentiation 145, 100897. 10.1016/j.diff.2025.100897 40753855

[B219] Zhang N. Wang X. Li Y. Lu Y. Sheng C. Sun Y. (2025). Mechanisms and therapeutic implications of gene expression regulation by circRNA-protein interactions in cancer. Commun. Biol. 8, 77. 10.1038/s42003-024-07383-z 39825074 PMC11748638

[B220] Zhang T. Liu Z. Wei Y. Lu J. He Z. Wu Z. (2025). Extracellular vesicles as natural nanocarriers: from in vitro engineering to in situ generation in cancer therapy. Chem. Eng. J. 510, 161653. 10.1016/j.cej.2025.161653

[B221] Zhang X. Zhang X. Deng X. Cao J. Bao Q. Wang H. (2025). Prostate cancer stem cell dynamics in the evolution of drug resistance. Int. J. Surg. 111, 5403–5419. 10.1097/JS9.0000000000002611 40465775

[B222] Zhang Z. Chen B. Liu Y. Zhang K. Wei Z. Dai Y. (2025). Advances in nanomedicine for targeting cancer stem cells and overcoming therapeutic resistance. ACS Nano 19, 30720–30757. 10.1021/acsnano.5c02321 40838668

[B223] Zhang B. Ma X. Zhou Y. Zhu B. Yu J. Liu H. (2026). Diagnostic value of circulating microRNAs for hepatocellular carcinoma: results of a meta-analysis and validation. Biochem. Genet. 64, 42–64. 10.1007/s10528-024-11001-2 39751721 PMC12882954

[B224] Zhang C. Yang K. Zhao Z. Feng M. Song L. Xu Z. (2026). piRNA: molecular mechanisms from germline silencing to somatic regulation and roles in disease. Int. J. Mol. Sci. 27, 2685. 10.3390/ijms27062685 41898544 PMC13026993

[B225] Zhong Y. He J.-W. Huang C.-X. Lai H.-Z. Li X.-K. Zheng C. (2025). The NcRNA/Wnt axis in lung cancer: oncogenic mechanisms, remarkable indicators and therapeutic targets. J. Transl. Med. 23, 326. 10.1186/s12967-025-06326-4 40087753 PMC11907837

[B226] Zhou Y.-L. Yao W.-L. Chen S.-H. Wang P. Fu J.-W. Zhao J.-Q. (2025). Global research landscape and emerging trends of non-coding RNAs in prostate cancer: a bibliometric analysis. Front. Pharmacol. 15, 1483186. 10.3389/fphar.2024.1483186 39845793 PMC11753231

